# Delayed Puberty—Phenotypic Diversity, Molecular Genetic Mechanisms, and Recent Discoveries

**DOI:** 10.1210/er.2018-00248

**Published:** 2019-04-09

**Authors:** Sasha R Howard, Leo Dunkel

**Affiliations:** Centre for Endocrinology, William Harvey Research Institute, Barts and the London School of Medicine and Dentistry, Queen Mary University of London, London, United Kingdom

## Abstract

This review presents a comprehensive discussion of the clinical condition of delayed puberty, a common presentation to the pediatric endocrinologist, which may present both diagnostic and prognostic challenges. Our understanding of the genetic control of pubertal timing has advanced thanks to active investigation in this field over the last two decades, but it remains in large part a fascinating and mysterious conundrum. The phenotype of delayed puberty is associated with adult health risks and common etiologies, and there is evidence for polygenic control of pubertal timing in the general population, sex-specificity, and epigenetic modulation. Moreover, much has been learned from comprehension of monogenic and digenic etiologies of pubertal delay and associated disorders and, in recent years, knowledge of oligogenic inheritance in conditions of GnRH deficiency. Recently there have been several novel discoveries in the field of self-limited delayed puberty, encompassing exciting developments linking this condition to both GnRH neuronal biology and metabolism and body mass. These data together highlight the fascinating heterogeneity of disorders underlying this phenotype and point to areas of future research where impactful developments can be made.

**Essential Points**

- The timing of puberty has a near-normal distribution in the general population, with the definitions of significantly early or delayed puberty being statistically delineated
- Pubertal timing is strongly determined by genetics, but it also depends on environmental factors such as body mass, nutrition, psychosocial factors, and, potentially, endocrine-disrupting chemicals
- Self-limited delayed puberty is the commonest cause of delayed puberty in both sexes, but only a small number of genetic causes of self-limited delayed puberty are known
- Other genetic causes of delayed puberty include mutations in GnRH deficiency genes and primary hypogonadism
- Gene discovery in delayed puberty is expanding rapidly through both next-generation sequencing and genome-wide association approaches
- The importance of the epigenetic control of pubertal timing and how epigenetic mechanisms mediate the influence of environmental factors on the timing of puberty represent a recent and fascinating area of discovery within puberty research

## Delayed Puberty: Definition and Morbidity

### Definition

In girls the first physical marker for the onset of puberty is most often the transition from Tanner breast stage B1 to B2, which includes early growth of the breast tissue. In boys the respective marker is the change from Tanner genital stage G1 to stage G2, including enlargement of the testes (*i.e.*, achievement of volume >3 mL or testicular length ≥25 mm) (1, 2). Development of pubic hair (pubarche) is usually not regarded as a sign for pubertal onset because pubarche may result from maturation of the adrenal glands (adrenarche), and the appearance of pubic hair can be independent of hypothalamic–pituitary–gonadal (HPG) axis activation.

Adrenarche refers to the maturation of the zona reticularis of the adrenal gland, resulting in increased production of adrenal androgens associated with secondary sexual characteristics such as the development of pubic and axillary hair, body odor, and acne. Adrenarche typically begins at the age of 8 years, but it can occur as early as 6 years. Similar to gonadarche (puberty), the onset of adrenarche appears to be a gradual, progressive maturational process that begins in early childhood and is marked by the further increases of production of adrenal androgens (3). Adrenarche may precede true puberty by 1 to 2 years in boys and girls, but the timing of clinical signs can vary. Although adrenarche and puberty often overlap, they are separate processes that are independently regulated (4, 5).

Originally, Marshall and Tanner reported the mean (±SD) onset of puberty to be 11.15 (±1.10) years in girls and 11.64 (±1.07) years in boys. These pubertal stages were based on photographic observation of genital development of a longitudinal, but still relatively small, sample of 192 girls and 228 boys living in a children’s home. Despite the probably poorly representative nature of this sample, comparable studies in Switzerland (6, 7), the United States (8), and Denmark (9) reported roughly similar mean ages of puberty onset. Although the mean age of onset may be fairly uniform, the onset of puberty takes place across a wide range of ages in normal, healthy adolescents. Several pathological states influence the timing of puberty either directly or indirectly and contribute to this disparity, but the great majority of the variation in pubertal timing cannot be attributed to any clinical disorder. In most populations 95% of girls experience onset of pubertal development between 8.5 and 13 years of age and the same percentage of boys between 9 and 13.5 years of age (9–14). These data have led to the traditional definition of delayed puberty as lack of development of secondary sexual characteristics by the age of 13 years in girls and 14 years in boys. However, these limits do not apply to all ethnic groups.

Because of the downward trend in pubertal timing, further discussed below, in some but not all reports from the United States (12, 15–18) and other countries (13, 14), some advocate for younger age cutoffs also for the general population. However, the secular change reported in the general population in the onset of puberty has not been consistently seen in late developing adolescents (14) and hence the need to readjust age definitions for delayed puberty in males may not be necessary.

### External factors and secular trends in the timing of puberty

The mean age of menarche in mid-19th century Europe was likely between 17 and 18 years of age (19). Starting from the late 19th century to the mid-20th century, a gradual decline in age at puberty has been reported, more convincingly in girls than in boys (13–15, 19–21), after which this trend may have leveled off. The change in the timing in puberty has likely been the result of better hygiene and nutrition as well as increased stability in socioeconomic conditions.

In recent decades secular changes toward earlier pubertal timing has reemerged, particularly in girls, probably reflecting changes in lifestyle and/or environmental factors, which can either be independent regulators or mediate their effects through genes by environment interactions [for reviews, see Refs. (19, 22–25)]. Variables such as increased adiposity, insulin resistance, physical inactivity, psychological factors, and changed dietary habits have all been implicated as possible mediators of the observed change in pubertal timing.

The extent to which age at puberty has declined in males during the past few decades is controversial. In the mid-1990s, data from the Third National Health and Nutrition Examination Survey (NHANES III), where genital ratings were performed by visual inspection, reported earlier age at puberty in both boys and girls (12, 18) than what previously had been reported from the United States (26). However, owing to lack of data on pubertal onset in the previous population-based study (Third National Health Examination Survey) (10), some controversy remained as how to interpret the NHANES III findings (12, 18). Furthermore, questions have been raised regarding the criteria used for genital staging in NHANES III (27, 28). A subsequent secular trend analysis between the Third National Health Examination Survey (which lacked data from the early pubertal stages) and NHANES III did not find clear evidence supporting earlier age at puberty. These data were also reviewed by an expert panel, which concluded that the available data are insufficient in quality and quantity to confirm a recent change in pubertal timing in US boys (15). Conversely, at the same time in Europe, in comparison with NHANES III studies, markedly higher ages at pubertal onset in boys have been reported (6, 7, 14, 29). Only a few studies contained data to assess secular trend in the timing of puberty in Europe. Of these, earlier studies did not support change in the age at pubertal onset in boys from the mid-1960s to the late 1990s (9, 29), whereas more recent studies report some evidence (14). The possibility that the increasing rates of obesity contributed to the secular trend toward early puberty onset was originally proposed in the report by Herman-Giddens *et al.* (16) in 1997. However, research to date highlights inconsistencies in how obesity has been found to affect pubertal timing, especially in boys, and emphasizes the need for future research in this area.

### Associations between delayed puberty and adult health risks

Menarche, the onset of first menstruation in girls, represents a distinct event, which is reasonably well recalled into adulthood. Therefore, this marker of puberty timing has often been included in epidemiological studies on the association of puberty timing and adverse health outcomes in the general population (19). Such studies report evidence that early age at menarche (AAM) is associated with higher risk of obesity in adulthood (30), type 2 diabetes (30), and cardiovascular disease (31). Other reported associations with early AAM include higher risk for breast cancer (32) and all-cause mortality (33). Furthermore, inverse genetic correlations are observed with polycystic ovary syndrome, fasting insulin levels, triglyceride levels, and bone mineral density (34). In men, owing to the lack of similar, convenient, and frequently recorded markers of puberty timing, reported associations with adverse health outcomes are less well described. Similar to menarche in girls, voice breaking represents a distinct marker of late stage of puberty in boys.

In both women and men, significant genetic correlations are observed between puberty timing and body mass index (BMI) (35). This strong interrelationship limits the assessment of their distinct influences on disease risks in traditional observational studies. For instance, large studies using historical growth records (estimating the age at pubertal growth spurt) have identified lower adolescent BMI and earlier puberty timing as predictors of higher breast cancer risk in women (36). Conversely, BMI is positively associated with breast cancer risk in postmenopausal women (37). Recently, Mendelian randomization analyses including adjustment for genetically predicted BMI have been used to assess the BMI-independent effects of AAM on the risks for various sex steroid–sensitive cancers (38). In such BMI-adjusted models, increasing AAM (i.e., later pubertal timing) is associated in particular with lower risk for estrogen receptor–positive breast cancer (38). Similarly, later AAM adjusted for genetically predicted BMI is associated with lower risks for endometrial and ovarian cancers. A protective effect of later puberty timing was also reported on risk for prostate cancer in men, independent of BMI (38).

In women, but not in men, late pubertal timing has been associated with higher risk for osteoporosis (39). The UK Biobank study, comprising ∼500,000 UK individuals, has provided an opportunity to study large-scale disease correlates. A recent study reports 19 adverse health outcomes for late menarche, and following adjustment for potential confounding and mediation by socioeconomic position and adiposity/body composition, six adverse associations remained significant for late menarche (34). These included notable novel associations for late menarche with higher risks for early natural menopause, malabsorption/celiac disease, low intelligence, asthma, poor sleep, and poor overall health.

In men older voice breaking was associated with anxiety/panic attacks, asthma, eczema, depression, and poor overall heath (35). However, these associations must be approached with some caution because of health selection bias and the possibility of reverse causality. Given that some pathological processes can have their origin long before the diagnosis or presentation of the disease, it remains possible that some factors originating in childhood may have influenced pubertal timing even though the specific condition is not diagnosed until later in life. This may be particularly relevant to the associations between late puberty timing and celiac disease and asthma.

Delayed puberty is often concerning to patients and families. It can affect psychosocial well-being and peer relationships, and these issues are common reasons for initiating sex steroid therapy. However, further studies are still needed to assess fully the psychosocial distress experienced by individuals with delayed puberty, whether this distress has long-term sequelae, and what impact sex steroid supplementation has on these outcomes (40). Patients, families, and practitioners are also often worried that delayed puberty may affect adult stature, and many patients present with relative familial short stature along with delayed puberty, which accentuates concerns about adult stature. Adult height can indeed be affected by delayed puberty, but on average it is only slightly below the genetic target (41). It remains also unclear whether the reduced adult bone mass and density represent a medical reason to initiate sex steroid therapy for the advancement of puberty (42).

## Differential Diagnosis of Delayed Puberty

### Common causes of delayed puberty and the prevalence of different etiologies

The pathogenesis of delayed puberty encompasses several conditions, but it is most commonly due to self-limited delayed puberty (also known as constitutional delay of growth and puberty). There are three main groups of differential diagnoses of self-limited delayed puberty (Table 1): functional hypogonadism, disorders causing primary hypogonadism, and GnRH deficiency leading to hypogonadotropic hypogonadism (HH), although up to 30 different etiologies underlying delayed puberty have been identified.

**Table 1.** Differential Diagnoses of Self-Limited Delayed Puberty

Common Causes of:		
Hypergonadotropic Hypogonadism	HH	Functional HH
Male	Isolated HH	Inflammatory bowel disease
Klinefelter syndrome	Klinefelter syndrome	Celiac disease
Congenital anorchia/testicular regression	Combined pituitary hormone deficiency	Anorexia nervosa
Mumps orchitis, coxsackie virus		Hypothyroidism
Female	CNS tumors/infiltrative diseases	Excessive exercise
Turner syndrome	Chemotherapy/radiation therapy	
Premature ovarian insufficiency		
Both		
Disorders of sexual development		
Gonadal dysgenesis		
Chemotherapy/radiation therapy		
Galactosemia		

[Table modified and reprinted with permission from Palmert MR, Dunkel L. Clinical practice. Delayed puberty. N Engl J Med. 2012;366:443–453.]

The absence of pathological medical history, signs and symptoms, and a positive family history of pubertal delay in one or both of the parents suggest a diagnosis of self-limited delayed puberty; however, before making the diagnosis, significant pathological conditions must be excluded. These include the aforementioned differential diagnoses of delayed puberty (Table 1): functional HH, where late pubertal development is due to maturational delay in the HPG axis secondary to chronic disease (found in ∼20% of subjects with delayed puberty), malnourishment, excessive exercise, and psychological or emotional stress; hypergonadotropic hypogonadism, with primary gonadal failure leading to elevated gonadotropin levels due to lack of negative feedback (found in ∼7% of male patients and 26% of female patients with delayed puberty); and permanent HH, characterized by low LH and FSH levels (9% of boys and up to 20% of girls) (43).

#### Self-limited delayed puberty

Self-limited delayed puberty represents the commonest cause of delayed puberty in both sexes. The term “self-limited” has become popular, as in the absence of an identifiable underlying cause pubertal onset usually occurs by the age of 18 years. Moreover, not all patients with such “simple” delayed puberty have constitutional features such as growth delay. Up to 83% of boys and 30% of girls with pubertal delay have self-limited delayed puberty. Individuals with self-limited delayed puberty lie at the extreme end of normal pubertal timing, with the absence of testicular enlargement in boys or breast development in girls at an age that is 2 to 2.5 SD later than the population mean. Additionally, self-limited delayed puberty may also encompass older children with slow pubertal progression, a diagnosis that is aided by the use of puberty normograms (44). Self-limited delayed puberty is no longer considered to be a benign developmental variant with no long-term consequences (see above).

Genetic influence on the timing of puberty is of fundamental importance, with epidemiological studies and genetic approaches estimating that 50% to 80% of the variation in pubertal onset is under genetic control (45). Although the timing of pubertal onset varies within and between different populations, it is a highly heritable trait, as shown by the high correlation of the timing of sexual maturation within families and in twin studies. Despite this strong heritability, little has been known about the mechanisms that control the timing of pubertal onset or its progression. Attempts to identify key genetic regulators have ranged from genome-wide association studies (GWASs) of AAM examining pubertal timing in healthy women to next-generation sequencing approaches to identify causal mutations in disease cohorts with delayed, absent, or precocious puberty. Although a predominance of males presenting with the condition has been noted, this may be a consequence of referral bias. For the specific genetic causes of self-limited delayed puberty, please see “Single-Gene Disorders Informing the Genetics of Pubertal Timing” below.

#### Congenital HH

Congenital HH (CHH) is defined by the diagnosis of gonadotropic deficiency during the infant mini-puberty or in adolescence when puberty is absent or arrested (46). More rarely, CHH may be suspected in adulthood due to infertility. A picture of “idiopathic” HH with no associated anatomical or functional defect in the HPG axis occurs in 1 to 10 cases per 100,000 births. Kallmann syndrome (KS; HH associated with anosmia) is the most common form of isolated HH (47–49).

The prevalence of CHH is higher in males, estimated at between 1 out of 4000 and 1 out of 10,000 males, and is reported to be twofold to fivefold less frequent in females (46, 50, 51). The molecular determinism of this sex difference is not well understood, and it probably reflects the sexual dimorphism of the gonadotropic axis. CHH may be sporadic or familial. CHH was initially considered a monogenic disorder. The understanding of the genetic basis of CHH has greatly advanced during the last 20 years (Table 2). Several modes of transmission have been described: X-linked recessive transmission, autosomal recessive transmission, autosomal dominant transmission, or transmission linked to an imprinting locus. Because of different causes and incomplete penetrance, there is a wide spectrum of phenotypes, ranging from complete HH, with lack of pubertal development, to partial hypogonadism with an arrest of pubertal development, and even reversible HH in some patients after treatment (52–55). For the various disease mechanisms and specific genetic causes of CHH, please see “CHH” under “Single-Gene Disorders Informing the Genetics of Pubertal Timing” below.

**Table 2.** Genetic Basis of GnRH Deficiency and Associated Features

	OMIM ID	Isolated	KS	Syndromic	Pathogenic Variants	Associated Variants
Primary/congenital						
*GNRHR/GNRH1*	138850/152760	X			x	
*KISS1R/KISS1*	604161/603286	X			x	
*TACR3/TAC3*	162332/162330	X			x	x
*FGFR1/FGF8*	136350/600483	X	x	Hartsfield	x	x
*FGF17*	603725	X	x			x
*ANOS1 (KAL1)*	300836		x		x	
*HS6ST1*	604846	X	x			x
*IL17RD*	606807		x			x
*DUSP6*	602748	X	x			x
*SPRY4*	607984	X	x			x
*FLRT3*	604808		x			x
*PROKR2/PROK2*	607123/607002		x		x	
*SEMA3A/SEMA3E/SEMA7A*	603961/608166/607961		x			x
*WDR11*	606417	X	x		x	
*CCDC141*	616031	X	x			x
*LEPR/LEP*	601007/164160			Severe obesity	x	
*PCSK1*	162150			Obesity, ACTH deficiency, diabetes	x	
*DMXL2*	616113			Polyendocrinopathy-polyneuropathy syndrome	x	
*RNF216/OTUD4*	609948/611744		x	Gordon Holmes	x	
*PNPLA6*	603197		x	Gordon Holmes, Oliver–Mcfarlane, Laurence–Moon	x	
*SOX10*	602229		x	Waardenburg	x	
*FEZF1*	613301		x			x
*CHD7*	608892	X	x	CHARGE	x	
*POLR3A/POLR3B*	614258/614366			4H	x	
*LHB*	152780	X			x	
*FSHB*	136530	X			x	
*NR0B1*	300473			Adrenal hypoplasia	x	
Associated with other pituitary hormone deficiencies						
Congenital		With or without midline defects; with or without developmental defects				
Secondary		Tumors: craniopharyngioma, germinoma, astrocytoma, glioma				
		Rathke pouch cyst				
		Brain (pituitary) irradiation				
		Head trauma				
		Infiltrative diseases: hemochromatosis, histiocytosis, sarcoidosis				
Functional, secondary to		Chronic diseases: gastrointestinal (celiac disease, inflammatory bowel disease) Endocrinopathies: hypothyroidism, hyperprolactinemia, GH deficiency Psychiatric illness: anorexia nervosa Excessive exercise, undernutrition				

The clinical presentation of CHH is related to the severity of GnRH deficiency and to associated biological features (46, 54, 56, 57). The severity of gonadotropic axis deficiency determines the phenotype at birth but also at adolescence. At least in boys, at birth a suspicion of hypogonadism may be raised by the assessment of genital appearance. In conditions of congenital GnRH deficiency, both fetal and postnatal pituitary gonadotropin secretion is low. Consequently, boys with CHH may have micropenis and cryptorchidism at birth, with prevalence ranging from 7% to 25% (58). The incidence of CHH in isolated congenital undescended testes has been reported to be as high as 70% (46, 59). Additionally, although puberty is recognized as the maturational process of the reproductive endocrine system that results in achievement of adult body proportion and the capacity to reproduce, mini-puberty has also been increasingly recognized as vital for normal fertility development (60–63). Mini-puberty provides a window of opportunity for evaluation of the functionality of the HPG axis before puberty (64–66).

During childhood, the gonadotropic axis is dormant, and LH is only detectable by ultrasensitive assays, whereas FSH plasma concentrations are variable (67, 68). The diagnosis of CHH is difficult to establish during this period (69–71). CHH is frequently diagnosed in adolescence due to a lack of initiation of puberty. The diagnosis of CHH in boys at puberty or in adulthood without clinical signs at birth suggests a partial form of gonadotropic deficiency (46). Owing to the absence of a phenotype at birth in females, this correlation is of course not true.

In addition to the endocrine phenotype, CHH may be suspected by the presence of associated clinical features. This association helps to classify CHH into three categories and points toward the underlying pathogenic mechanism (46). The first group is composed of isolated CHH. The association of CHH with anosmia defines KS as a second group. Hearing impairment and skeletal abnormalities such as ectrodactyly, synkinesia (upper limb mirror movements), cleft lip/palate, and hypodontia may also be observed in KS. In addition to these relatively common clinical features, CHH may also be a component of a more complex syndrome (syndromic CHH). Obesity, abnormal behavior, ataxia, mental disability, neuropathy, or white matter disorder may be observed in these syndromes. In few cases, CHH may be associated with a neurodegenerative process starting in adolescence (72).

Patients with CHARGE syndrome may be associated with central hypogonadism (73, 74). CHARGE stands for coloboma, heart malformations, atresia of the choanae, retardation of growth and development, genital anomalies, and ear anomalies (auditory and vestibular) (75). Additionally, CHH may be present, and most patients with CHARGE syndrome have olfactory bulb aplasia. CHARGE syndrome has an estimated birth incidence of 1 in 8 out of 500 to 12,000 (76). Other infrequently occurring features include characteristic face and hand dysmorphia, hypotonia, arhinencephaly, semicircular canal agenesis or hypoplasia, hearing impairment, urinary tract anomalies, orofacial clefting, dysphagia, and tracheoesophageal anomalies. Multiple sets of diagnostic criteria for CHARGE syndrome have been proposed (77). The causative chromodomain helicase DNA binding protein 7 (*CHD7*) gene encodes a chromodomain (chromatin organization modifier domain) helicase DNA–binding protein expressed in the olfactory placode, which gives rise to GnRH neurons, spinal cord, nasopharynx, and eye. This protein may explain some of the organ involvement. Most patients are heterozygous for loss-of-function mutations in *CHD7* (75). Loss-of-function mutations in *CHD7* may also be found in patients with CHH without associated syndromic features.

#### Central nervous system tumors

Tumors of the central nervous system (CNS) causing delayed puberty most commonly interfere with GnRH synthesis or secretion. These include craniopharyngioma (78, 79), Langerhans cell histiocytosis (80, 81), germinomas, and prolactinomas. Germinomas are the most common extrasellar tumors to cause delayed puberty, although these tumors are a rarity among primary CNS tumors (82). Deficiency of other pituitary hormones is commonly associated with these tumors. Associated posterior pituitary hormone deficiencies are often manifested by diabetes insipidus. Treatment of CNS tumors, leukemia, or neoplasms with cranial irradiation may result in gradual development of hypothalamic–pituitary failure (83). GH deficiency is the most common component of the radiation-induced hormone disorder, but gonadotropin deficiency also occurs when the radiation dose is high enough. Development of radiation-induced hypothalamic–pituitary failure may take from 1 year to several years to ensue (84). The estimated prevalence of gonadotropin deficiency in childhood cancer survivors is 10.8%. The most recent guidelines to be published recommend screening for gonadotropin deficiency in childhood cancer survivors exposed to hypothalamic–pituitary axis radiation at doses ≥30 Gy and in those with a history of tumors or surgery affecting the hypothalamic–pituitary axis region (85).

#### Developmental defects of the CNS

Various malformations affecting the development of the prosencephalon may cause delayed puberty combined with deficiency of any or all other pituitary hormones (86). Midline malformations are often associated with optic dysplasia, and an absent septum pellucidum is often found by imaging techniques (septo-optic dysplasia). Other congenital midline defects, which may range from holoprosencephaly to cleft lip and palate, may also be associated with variable hypothalamic–pituitary dysfunction (86).

Genetic defects affecting development of the anterior pituitary cause hypopituitarism, including CHH, in some cases. The pituitary transcription factors *HESX1*, *LHX3*, and *SOX2* are vital for early patterning of the forebrain and pituitary, and mutations in these developmental genes result in syndromic hypopituitarism with gonadotropin deficiency in humans. *PROP1* is important for the development of gonadotropin-secreting cells, and autosomal recessive mutations in this gene are the most common cause of combined pituitary hormone deficiency in humans (87). *PITX2* is also vital for survival of gonadotrope cell lineage and is required for expression of the gonadotrope-specific transcription factors *GATA2*, *EGR1*, and nuclear receptor subfamily 5 group A member 1 (*NR5A1*).

The nuclear receptor subfamily 0 group B member 1 (*NR0B1*) gene, alternatively known as DAX-1 orphan nuclear receptor (*DAX1*) gene, and *NR5A1*, alternatively known as steroidogenic factor-1 (SF1), are important for the development of the adrenal gland, gonads, ventromedial hypothalamus, and pituitary gonadotrope cells (88). Mutations in *NR0B1* cause X-linked adrenal hypoplasia congenita, with associated HH, whereas mutations in *NR5A1* are associated with 46,XY sex reversal or gonadal dysgenesis, and 46,XX is associated with premature ovarian insufficiency (88). Leptin and prohormone convertase-1 may also influence GnRH release and processing of the GnRH receptor, with mutations resulting in a phenotype of HH (89).

#### Functional HH

##### Chronic disease.

A wide variety of childhood diseases (many with chronic inflammation) such as Crohn disease (90), celiac disease (91), chronic kidney disease (92), cystic fibrosis (93), sickle cell disease, and juvenile idiopathic arthritis are associated with an increased likelihood of delayed puberty. This is a result of several factors related to the disease itself, such as malnutrition, hypercortisolemia, and elevated levels of proinflammatory cytokines. In malnutrition and chronic diseases, weight loss below the level of 80% of ideal body weight can cause delayed or arrested pubertal development (94). Nutrition plays an important yet uncharacterized role in the control of GnRH secretion. For example, in regional enteritis, gonadotropin secretion remains normal when nutrition is optimally balanced, but a nonoptimal nutritional status will result in a hypogonadotropic state and arrested pubertal maturation (90). Poor nutrition also contributes to the decrease in height velocity usually observed in these patients, decreased bone mineral density, and low mood. Chronic renal insufficiency delays pubertal development, but after successful renal transplantation, gonadotropin secretion is usually restored (95).

Most endocrinopathies can cause functional HH with delayed puberty, arrested puberty, or functional amenorrhea (96). The most common scenario is hypogonadism due to a prolactinoma, and here the pathophysiology is mediated by hyperprolactinemia itself or via interference with the inhibitory effect of dopamine on prolactin secretion, by the suppression of GnRH by excess cortisol, and/or by hyperandrogenemia. Additionally, GH-secreting adenomas, especially macroadenomas, can also compromise the gonadotrophin cells via mass effect, leading to acquired HH in patients with gigantism or acromegaly (97). Treating the underlying endocrinopathy usually results in normalization of the HH axis, although this may not recover following treatment of a significant macroadenoma.

##### Anorexia.

Anorexia nervosa is usually associated with severe or even fatal weight loss, which is due to distorted body image, obsessive fear of obesity, and avoidance of food. Virtually all patients have primary or secondary amenorrhea (98). Functional HH is at least partly due to severe weight loss, but amenorrhea may also precede the onset of weight loss (99). The underlying pathophysiology of amenorrhea is due to GnRH deficiency because the LH secretory pattern in pubertal-aged girls with anorexia is similar to that seen in girls during prepuberty: low or absent LH pulses and a blunted LH response to exogenous GnRH (100). This may be mediated, at least in part, by leptin, as women with anorexia have also been demonstrated to have lower leptin concentration than do controls (101, 102). Long-term pulsatile administration of GnRH has been shown to restore a pubertal pattern of LH secretion, confirming the hypothalamic location of the defect. Administration of leptin can also reverse hypogonadism in women with hypothalamic amenorrhea, suggesting a potential role for leptin in the treatment of anorexia (102, 103). Recovery of normal weight will normalize most endocrine and metabolic functions, but amenorrhea may persist for years (104).

##### Athletic training.

Overly intensive exercise may suppress the HPG axis by inhibiting hypothalamic pulsatile secretion of GnRH, arrest pubertal development, and cause amenorrhea in females (105). These disorders often include compulsive endurance training and are common especially among long-distance runners, gymnasts, and ballerinas. HH may develop even when athletes have normal weight but have less fat and more muscle compared with nonathletic individuals. In female athletes with delayed or arrested pubertal development, adrenarche usually takes place at the normal age. The mechanism of delayed puberty is unclear, but interruption of intensive training advances puberty and menarche before any change in body composition or weight, suggesting a direct effect of physical activity on GnRH secretion. However, certain genetic mutations may predispose to the development of all types of functional HH, and there is evidence for overlap between the genetic bases of functional HH and GnRH deficiency (106).

#### Hypergonadotropic hypogonadism

Conditions of primary gonadal failure are listed in Table 1. Elevated serum gonadotropins occur usually by the time of the physiological age of puberty. During middle childhood, serum gonadotropins may be similar or mildly higher than those from normal controls (107). In boys, low serum inhibin B reflects primary germ cell failure.

In gonadal dysgenesis in both males and females, delayed or absent pubertal development may be the presenting complaint, although associated features usually predominate. Turner syndrome is the most common form of hypergonadotropic hypogonadism in females, occurring in 1 in 2000 to 2500 live births (108). In Turner syndrome, puberty is usually absent, or otherwise delayed, and is followed by progressive ovarian failure (109). Importantly, however, up to 30% of girls will undergo spontaneous pubertal development and 2% to 5% will have spontaneous menses (110). About half of girls with Turner syndrome have the 45,X karyotype. Other causes of ovarian dysgenesis include X isochromosome, where abnormal chromosome division results in duplication of identical chromosome arms, most commonly of the long (q) arm. Various deletions and duplications of the short and long arm of the X chromosome are also found in women with primary ovarian insufficiency, with several genes implicated, including fragile X mental retardation 1 (*FMR1*), premature ovarian failure 1B (*POF1B*), diaphanous related formin 2 (*DIAPH2*), forkhead box L2 (*FOXL2*), and bone morphogenetic protein 15 (*BMP15*) (111). Point mutations in the extracellular domain of the FSH receptor are mostly restricted to the Finnish population and result in inactivation of the receptor function with primary or secondary amenorrhea (112).

In males, testicular abnormalities are characterized by elevated gonadotropin and low inhibin B concentrations, and may present as pubertal delay. The commonest condition underlying hypergonadotropic hypogonadism in males is Klinefelter syndrome (47,XXY), with a prevalence of 1 in 667 live births. Most of those affected will enter puberty spontaneously at a normal age (113), but testosterone levels become increasingly deficient by Tanner stages 4 to 5, possibly as a result of secondary regression (114). Delayed puberty may be seen in those with a more complex karyotype (48,XXYY, 48,XXXY, 49,XXXXY). Bilateral anorchia may also be due to vanishing testis syndrome.

Many other causes of disorders of sex development are associated with gonadal failure, but discussion of these is beyond the scope of this review (115).

Several complex syndromes may have been associated with hypergonadotropic hypogonadism, including Down syndrome, hypogonadism associated with myopathies (myotonic dystrophy and progressive muscular dystrophy), and Prader–Willi (116), Werner (117, 118), and Alström (119) syndromes. In Noonan syndromes and related disorders, testicular abnormalities are less severe (120).

##### Gonadotropin receptor mutations.

Several homozygous or compound heterozygous loss-of-function mutations in the *LHCGR* gene have been described in males and females (121). The presentation in females is usually primary amenorrhea rather than delayed puberty. In XY males, lack of virilization during the fetal development results in a female phenotype with absence of Müllerian structures and absence of Leydig cells in the testis. Serum levels of LH are elevated and FSH levels are normal. Homozygous mutations in the follicle-stimulating hormone receptor (*FSHR*) are extremely rare, affecting mostly females with variable degree of pubertal development and complete ovarian failure. Discovered first in the Finnish population, point mutations in the extracellular domain of the *FSHR* lead to subsequent inactivation of the receptor function, resulting in raised FSH levels (112). Whereas up to 40% of Finnish patients with premature ovarian insufficiency have such a mutation, these appear to be rare in other populations. Histological examinations of ovarian biopsies show the presence of follicles in all female patients with FSH receptor defects, whereas only one in four of those with unknown etiology have follicles. Hence, whereas the receptor defect causes a specific arrest in follicular maturation, many patients with hypergonadotropic ovarian failure have true ovarian dysgenesis. The ovarian phenotype in patients with inactivating FSH receptor mutation is informative with regard to the role of FSH in the regulation of follicular development: the early phases of follicular maturation (up to the preantral stage) are independent of FSH, but for the final maturation of the follicle, this gonadotropin is absolutely necessary. Patients with FSH resistance generally have low to normal anti-Müllerian hormone (AMH) values, in contrast to women with primary ovarian insufficiency due to follicular depletion who have very low to undetectable AMH. In patients with FSH receptor mutations, small growing follicles will continue to secrete AMH, depending on the severity of mutation and the resultant stage of follicular arrest (122, 123). In males, serum LH and testosterone levels are normal, FSH levels are elevated, and there is variable suppression of spermatogenesis (124).

## Evaluation of a Patient With Delayed Puberty

### Complete clinical evaluation

**Figure 1.**
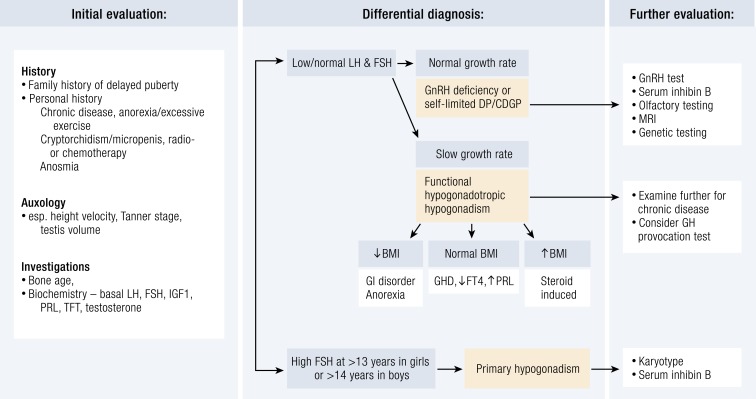
Flowchart for the evaluation of a patient with delayed puberty. CDGP, constitutional delay of growth and puberty; DP, delayed puberty; FT4, free T4; GHD, GH deficiency; PRL, prolactin; TFT, thyroid function test.

A temporary delay in sexual maturation is not uncommon and may resolve with time, leading to normal development, optimum adult height, and fertility. However, in patients with an underlying organic pathology, early diagnosis and treatment are essential to ensure normal pubertal progress and adequate adult height. A complete personal medical history must be taken in those presenting with delayed puberty, including height and weight charts, nutritional status, medications, history and/or symptoms of chronic disease, and psychosocial functioning (40, 125). A thorough history should also note evidence of anorexia and the intensity of athletic training (Fig. 1). A history of chronic illnesses, such as celiac disease and inflammatory bowel disease, may suggest a temporary or secondary delay of puberty. A complete family history, including childhood growth patterns, age at pubertal onset of both parents and siblings, and any history of infertility, anosmia, and midline abnormalities of parents and siblings, is required, as a positive familial history is common.

*“There is some evidence that the female HPG axis may be more sensitive than the male HPG axis to environmental factors….”*

Physical examination must include pubertal stage assessment in both sexes with penile size as well as location, size, and consistency of the testis in boys. Assessment of Tanner stages can help to identify early signs of puberty that have not been previously noticed. Children who have a low weight for height have an increased likelihood of having an underlying condition delaying HPG axis activation. Bilateral cryptorchidism or a small penis at birth and hyposmia or anosmia due to hypoplasia of the olfactory bulbs may suggest CHH. Lack of smell associated with KS can be assessed by means of detailed questioning or objectively by formal olfactory test, such as the Pennsylvania smell test (126). Certain other physical signs will increase suspicion of underlying CHH, such as cleft lip or palate, bimanual synkinesia, congenital ptosis and abnormal visual spatial attention, eye movement abnormalities, sensorineural hearing impairment, agenesis of one or several teeth (hypodontia), obesity, and features suggesting the CHARGE syndrome, as well as digital and other skeletal abnormalities (46). Delayed cognitive development associated with obesity or dysmorphic features may suggest an underlying genetic syndrome. A history of chemotherapy or radiotherapy may indicate primary gonadal failure or gonadotropin deficiency depending on the specific treatment received.

In CHH the diagnosis is typically made during the second or third decade of life. Common presenting signs are delayed onset of puberty, poorly developed secondary sexual characteristics, eunuchoid body proportions, or infertility (50). In some cases, the diagnosis can be suspected before the age of pubertal onset, as discussed above, during the mini-puberty. The presence or absence of “red flag” features remains the strongest discriminator between isolated delayed puberty and HH. The primary red flags are cryptorchidism or micropenis, indicating a lack of prior mini-puberty, but the presence of the other components of KS (*e.g.*, anosmia, cleft lip and palate, unilateral renal agenesis) increases the likelihood of the diagnosis (46).

Differential diagnosis between self-limited delayed puberty and CHH in boys who present with delayed puberty is often difficult at the time of referral, as both conditions may present with effectively the same clinical and hormonal features. Only the demonstration of a complete and effective puberty can distinguish isolated delayed puberty and CHH (partial or complete). Analysis of height velocity is very important in the assessment of individuals with delayed puberty (127). In most subjects with constitutional delay there is delayed maturation during early childhood, and consequently they may be shorter than their peers. Those delayed puberty subjects who also have poor growth in childhood may not fully exploit their genetic height potential, resulting in an adult height below their midparental target height (128–130), with an average loss of 4.2 cm when untreated (127). However, other studies showed only a negligible difference in adult height, even in delayed puberty subjects who have received no intervention (41, 131–137). This may imply a pathophysiological mechanism additional to lack of sex steroids contributing to the growth phenotype in some patients with delayed puberty, but not in others.

In contrast, patients with CHH have steady linear growth during childhood and only become short for their age with absence of the pubertal growth spurt (138). However, hypogonadotropic states cannot be ruled out by short stature and slow growth rate. In delayed puberty adrenarche may also occur later than usual, in contrast to the normal age of adrenarche in patients with isolated HH. Bone age in delayed puberty (X-ray film of nondominant hand and wrist with bone age assessed according to defined standards) is usually behind chronological age, but the developmental milestones are achieved at a normal bone age; that is, onset of signs of pubertal development by the bone age of 13 years in girls and 13.5 years in boys. Gonadotropin and testosterone concentrations increase in concert with the development of the bone age. Thus, all stages of pubertal development occur at an age later than usual.

Thus, initial screening in delayed puberty should include bone age, basal LH and FSH (to look for hypergonadotropic hypogonadism), early-morning testosterone (139), and biochemical analysis to search for asymptomatic systemic illness [full blood count, erythrocyte sedimentation rate (or C-reactive protein), renal function, celiac screen, liver function, electrolytes], and thyroid function test, IGF-I, and prolactin to assess other pituitary hormonal function (40). A karyotype is important especially in females with primary hypogonadism. Brain MRI (to examine olfactory bulbs and sulci) is used to exclude olfactory aplasia or hypoplasia or other hypothalamic–pituitary lesions.

Basal gonadotropin levels are often increased in primary hypogonadism due to, for example, Turner or Klinefelter syndrome, but the basal gonadotropin values are not useful in the differential diagnosis of self-limited delay and CHH. Investigation of the differential diagnosis of these latter two conditions may involve a number of physiological and stimulation tests, including assessment of LH pulsatility by frequent sampling, prolactin response to provocation, gonadotropin response to GnRH, testosterone response to hCG, and first morning-voided urine FSH and LH levels (140, 141). More recently, a single measurement of inhibin B <35 pg/mL in prepubertal boys has been shown to discriminate CHH from self-limited delay with high sensitivity (142), but the finding has not been replicated in other studies (143) and has not been conclusively demonstrated in girls (144). Collectively, testicular volume (cutoff of 1.1 mL), GnRH-induced maximal LH (cutoff of 4.3 IU/L), and basal inhibin B level have been proposed as the most effective discriminators of CHH from delayed puberty in adolescent males (138). However, follow-up is often warranted before a definitive diagnosis can be made. Other investigations may be required, such as pelvic ultrasound for gonad and uterine assessment and renal ultrasound in X-linked CHH, owing to suspected anosmin 1 (*ANOS1*) mutations that are associated with renal malformation or unilateral agenesis (46, 53).

### Assessment of the newborn

Boys with CHH may present with micropenis and/or cryptorchidism at birth (65). Primary hypogonadism may also present at birth with underdeveloped genitalia in male infants when the condition is gonadotropin-dependent, or alternatively as ambiguous or female genitalia when the defect is of early fetal onset resulting in disorders of sex development.

If a suspicion of congenital hypogonadism arises in the first 3 to 6 months of life it can be investigated on the basis of sex steroid and gonadotropin levels without the need for stimulation tests (65). Gonadotropin levels in healthy infants start to increase during the first week of life and then decrease toward the age of 6 months, except for FSH levels in girls that remain elevated until 3 to 4 years of age (60–63, 66). Testosterone levels in boys increase in response to LH levels and peak at 1 to 3 months of age, but in girls estradiol levels fluctuate, probably reflecting ovarian follicular growth and atrophy. Estradiol levels in girls decline in the second year of life. Postnatal HPG axis activation during mini-puberty has important roles in both sexes: in males, for penile and testicular growth, and in girls, for maturation of ovarian follicles and an increase in estradiol levels. However, most studies on hormone levels during mini-puberty have had cross-sectional design, and hence the interindividual differences in timing, duration, and magnitude of mini-puberty have remained largely unexplored. Serial blood sampling from healthy infants is problematic because of its invasiveness, and noninvasive urine or salivary sampling is a way around this problem; however, urine and saliva assays are not widely used in clinical routine. Recently, longitudinal data have provided new information about the hormonal patterns, including the timing of the peak hormone levels and the decrease in hormonal activity according to developmental age (60–63, 66)

In primary hypogonadism with gonadal dysgenesis, anorchia, or testicular regression, gonadotropin levels in mini-puberty are generally raised, but they may fall to normal levels in later childhood. However, in Turner syndrome, infant girls with the 45,X karyotype have higher FSH levels than do healthy girls, and the levels remain elevated for several years (107, 145). In contrast, girls with Turner syndrome with other karyotypes than 45,X often have close to normal FSH levels, suggesting some ovarian feedback effects on pituitary FSH secretion in these patients. Often, infant boys with Klinefelter syndrome (47,XXY karyotype) have normal levels of inhibin B, AMH, and INSL3, suggesting normal Sertoli and Leydig cell function in infancy, although they have elevated LH and FSH levels (146–150).

Newer markers of gonadal function are useful, particularly in males, for diagnosis of hypogonadism, both soon after birth and after mini-puberty is completed (151). Inhibin B is a useful marker of Sertoli cell function from the neonatal period into early childhood and can be used to assess male infants with micropenis and/or cryptorchidism, both due to central and primary hypogonadism (152). Its use in female infants is less clear (153). AMH is strongly expressed by Sertoli cells from the time of testicular differentiation to puberty and at much lower levels in females by the granulosa cells from birth until menopause (151). Undetectable AMH and inhibin B are considered diagnostic of anorchia, but low, close to undetectable levels are also seen in severe forms of CHH (151). In infant girls, a similar pattern in AMH levels during the first months of life has also been reported, but the levels in girls are significantly lower (61). Thus, low sex steroid and gonadotropin levels in an infant <3 to 6 months of age indicate central hypogonadism with an absence of the normal mini-puberty (66). In contrast, high gonadotropins associated with low/undetectable basal testosterone and INSL3 (in boys) are diagnostic of primary hypogonadism (115). Outside of the mini-puberty period, useful tests for the investigation of hypogonadism include inhibin B and AMH (64).

## Genetics of Pubertal Timing in the General Population

### GWASs in women

The existence of genetic heterogeneity determining the timing of puberty in the general population is supported by several large GWASs. Most of these studies have been based on self-recall of the timing of menarche, and have thus been carried out in women. The first of many loci associated with age of menarche was the gene *LIN28B*, which is a human ortholog of the gene that controls developmental timing in the *Caenorhabditis elegans* through miRNAs. The lin-28 family regulates the biogenesis of let-7 miRNA family members controlling the timing of developmental events and in turn let-7 miRNA controls lin-28 translation. The major allele of the single-nucleotide polymorphism rs314276 (located in intron 2 of *LIN28B*) was associated with earlier AAM and earlier breast development in girls (154). However, mutations in *LIN28B* have not yet been identified in human patients with delayed puberty (155) or in early puberty (156).

**Figure 2.**
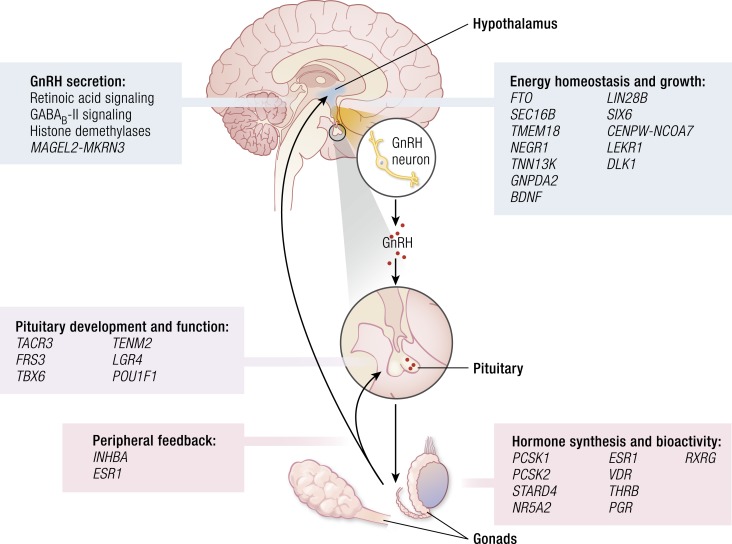
Possible roles in the HPG axis of several of the implicated genes from GWAS and biological mechanisms for menarche timing [adapted from Perry *et al.* (158)].

In 2010, a large meta-analysis identified 42 (30 new, 2 previously confirmed, and 10 possible) loci for AAM (157). In 2014, this was extended to encompass data from genome-wide and custom-genotyping arrays in up to 182,416 women of European descent from 57 studies (Fig. 2) (158). Evidence (*P* < 5 × 10^−8^) for 123 signals at 106 genomic loci was identified. Many of these loci were associated with Tanner staging in both sexes, suggesting that these data are applicable to both men and women. The largest GWAS to date comprises 1000 Genomes Project–imputed genotype data in up to ∼370,000 women and identifies 389 independent signals (*P* < 5 × 10^−8^) for AAM. Per-allele effect sizes ranged from 1 week to 5 months. These signals explain ∼7.4% of the population variance in AAM, corresponding to ∼25% of the estimated heritability, suggesting that many of these genetic variants have a low impact in the general population (38).

Importantly, genes already identified in rare disorders of puberty were identified from these GWASs. These included the imprinted gene makorin ring finger protein 3 (*MKRN3*), paternally inherited mutations that have been identified as causal in pedigrees of central precocious puberty (CPP), and delta like non-canonical Notch ligand 1 (*DLK1*). *MKRN3* and *DLK1* are to date only the third and fourth genes with mutations identified as causal in pedigrees of CPP, the others being kisspeptin 1 (*KISS1*) and its receptor *KISS1R* (also known as *GPR54*).

Signals in multiple genes already associated with the HPG axis were identified: near the leptin receptor (*LEPR–LEPROT*), which is also immediately upstream of tacykinin receptor 3 (*TACR3*), which encodes the neurokinin B receptor. A further variant ∼10 kb from *GNRH1* approached genome-wide significance. Two signals were found near prohormone convertase 1 and 2 (*PCSK1* and *PCSK2*), indicating a common function of these prohormone convertases in pubertal regulation. Signals in or near several further genes with relevance to pituitary development and function including POU class 1 homeobox 1 (*POU1F1*), teneurin transmembrane protein 2 (*TENM2*), and fibroblast growth factor (FGF) receptor substrate 3 (*FRS3*) and signals representing *cis*-expression quantitative trait loci for leucine-rich repeat–containing G protein–coupled receptor 4 (*LGR4*) and T-box 6 (*TBX6*), which both encode enhancers for the pituitary development factor *SOX2*, were identified.

In addition to leptin signaling, the authors found overlap with several genes implicated in BMI, including fat mass and obesity-associated protein (*FTO*), SEC16 homolog B (*SEC16B*), transmembrane protein 18 (*TMEM18*), and neuronal growth regulator 1 (*NEGR1*). The onset of puberty requires a minimum level of energy availability, whereas increased BMI has been shown to be associated with precocious onset of puberty. However, the molecular mechanisms for this are still unclear. Whether such genes may regulate pubertal timing exclusively via impact on body mass or via other BMI-independent mechanisms is as yet unknown.

Pathway analyses implicated nuclear hormone receptors, particularly those involved in retinoic acid (RA) and *γ*-aminobutyric acid (GABA)–B2 receptor signaling. The active metabolites of vitamin A, all-*trans* RA and 9-*cis* RA, have differential effects on GnRH expression and secretion. Other possible mechanisms linking RA signaling to pubertal timing include inhibition of embryonic GnRH neuron migration and enhancement of steroidogenesis and gonadotropin secretion.

The authors of these GWASs on age of menarche hypothesize that the genetic architecture of the timing of puberty in healthy subjects involves hundreds of common variants. These studies do rely on self-recall of the AAM, which may result in imprecise data.

### GWASs in men

The major allele of the single-nucleotide polymorphism rs314276 (located in intron 2 of *LIN28B*) was also found to be associated with earlier voice breaking and more advanced pubic hair development in boys and faster tempo of height growth and shorter adult height in both sexes (154). More recently, GWASs of pubertal timing have specifically addressed the timing of voice break in males (38, 154, 157, 158). Many of these signals have concordant effects on the age at voice breaking, a corresponding milestone in males. However, in women the signals identified had stronger effects on early than on late age of menarche, but in contrast they had larger effect estimates for relatively late than relatively early voice breaking in males (38). This would suggest a greater contribution of “normal” genetic variation to early maturation in girls, but to later maturation in boys.

### Epigenetic regulation of pubertal timing

Several different epigenetic mechanisms have been implicated in regulation of the timing of puberty. Experimental data from rats and goats give evidence for changes in histone acetylation and gene methylation leading to altered gene expression during puberty (159, 160). Such epigenetic regulators are potential mediators of the effects of the environment on the hypothalamic regulation of puberty. However, the link between environmental factors and epigenetic control of puberty via the hypothalamus has not been fully explained.

Imprinting is a further epigenetic phenomenon implicated in pubertal timing. Imprinted genes influence the timing of human weaning and adrenarche, with paternally-expressed genes promoting delays in childhood maturation and maternally-expressed genes promoting accelerated maturation (161). Paternally inherited variants in *MKRN3* and *DLK1* have been found to correlate with AAM in girls and voice breaking in boys from GWASs (38), as above. These two imprinted genes have been reported in familial disordered pubertal timing, both of which with paternally inherited mutations identified in pedigrees of CPP (162, 163). *MKRN3* is thought to contribute to the puberty “brake” restraining the HPG axis via inhibition of GnRH release. However, neither *MKRN3* nor *DLK1* mutations have been described in the pathogenesis of delayed puberty.

Prader–Willi syndrome (PWS), another syndrome frequently caused by imprinting disorders, is associated with absent or delayed puberty (164). Most cases of PWS are caused by deletion of a cluster of imprinted genes (which include *MKRN3*) on the paternally inherited copy of chromosome 15 (paternal deletion) or by inheritance of both copies of this cluster from the mother (maternal uniparental disomy) (165). The minority of individuals with PWS undergo precocious puberty (166), but most undergo incomplete puberty, expressed as lack of a pubertal growth spurt, HH, cryptorchidism, underdeveloped genitalia, or incomplete menarche (116). The rarity of precocious puberty in PWS, despite the absence of expression of *MKRN3*, is probably explained by the effects of other imprinted genes that are inactivated in typical cases of PWS such as MAGE family member L2 (*MAGEL2*) (167, 168). This evidence suggests a complex role for imprinted genes in the timing of puberty, and one in which a given gene’s activation can be specific both to tissue type and developmental stage (161, 165).

Recent evidence highlights the importance in mice of miRNAs (particularly the miR-200/429 family and miR-155) in the epigenetic upregulation of GnRH transcription during the critical period (murine equivalent of mini-puberty) (169). Moreover, miR-7a2 has been demonstrated to be essential for normal murine pituitary development and HPG function, with deletion in mice leading to hypogonadotropic infertility (170). This is discussed further in “Upstream control of GnRH neuronal function” under “Single-Gene Disorders Informing the Genetics of Pubertal Timing” below.

The effects of environmental changes on the hypothalamic regulation of puberty may be mediated in part via epigenetic mechanisms, and several studies have shown that the pubertal brain epigenome is affected by environmental perturbations (159). The effect of possible endocrine-disrupting chemicals (EDCs) on the timing of puberty has been an ongoing concern (171). Numerous substances, including polybrominated biphenyls, bisphenol A, atrazine (herbicides), and phthalates, as well as other more common medicines such as paracetamol and betamethasone, have been suggested as possible EDCs responsible for contributing to disruption of pubertal biology. For example, children migrating for international adoption and formerly exposed to the estrogenic insecticide DDT in their country of origin displayed early or precocious pubertal timing.

Whereas the window of opportunity for the effects of EDC exposure was historically considered to be in the late prepubertal period, evidence of fetal and neonatal origin of changes in pubertal timing counters this theory. Prenatal exposure in boys to EDCs such as phthalates is associated with reduced masculinization of genital structures (172). Moreover, maternal exposure to EDCs in rodents has been shown to cause epigenetic modifications in testis and other systemic effects, and thus epigenetic changes during fetal life are also a potential mechanism for the hypothalamic effects of EDCs *in utero* (22). The effects of EDCs may persist in pregnant rats in not only their unborn fetus but into the next generation as well.

However, a clear mechanism of action for EDCs through the early initiation of the pulsatility of GnRH from the hypothalamus has not been conclusively demonstrated. Studies are complicated by the likely differing, and possibly divergent, influence of different doses and combination of EDCs and differing effects depending on age and length of exposure (173). One recent study has demonstrated alteration in the hypothalamic expression of GnRH, LH, and upstream transcriptional regulators of GnRH including organic cation transporter 2 (Oct-2) and thyroid transcription factor-1 (Ttf-1) in female mice where their mothers were exposed to arsenic during pregnancy (174). These changes were associated with earlier vaginal opening, a marker of puberty onset in rodents.

### Sexual dimorphism in pubertal timing

There are fundamental differences between males and females in the dynamics of the reactivation of the gonadotropic axis at puberty onset. This biological reactivation of the HPG axis occurs earlier in girls than in boys. In females, estradiol increases together with increasing LH and FSH. In males, the secretion of testosterone increases shortly after the increase in the plasma concentration of LH and FSH.

In boys during puberty, plasma testosterone concentrations increase dramatically (175). The pubertal increase in testicular size results primarily from more proliferating and differentiating germ cells and, to a lesser extent, an increase in Sertoli cells. In early and mid-puberty there is a pronounced diurnal rhythm with a morning peak in measurable testosterone, but this is less pronounced in later puberty and declines gradually with age, probably due to decreased day/night ratios of gonadotropins (68, 176). In girls, a hormonal dialogue between gonads, hypothalamus, and pituitary contributes to the progressive activation of the gonadotropic axis until the end of puberty, with a gradual increase in GnRH stimulation resulting first in nocturnal LH secretion, with a cyclical pulsatile LH pattern including an LH surge establishing even before menarche (177).

These sex differences may be related to differing hormonal status, but they could also be a feature of the sexual dimorphism of the brain. In mice the expression of Kiss1 in the anteroventral periventricular nucleus is much more pronounced in females, whereas its promoter methylation levels are significantly higher (178). However, the latter may act as a block to repressive transcription factors, thus accounting for the increase in kisspeptin expression. Many genetic variants associated with AAM are also associated with age at voice breaking in males with the same direction of effects, as discussed above (38). As a consequence, early-maturing girls tend to have early-maturing brothers, and late‐maturing boys tend to have late‐maturing sisters, but the extent to which these variants have an effect may differ between the sexes.

There is some evidence that the female HPG axis may be more sensitive than the male HPG axis to environmental factors such as changes in fat mass, such as in conditions of functional hypogonadism due to weight loss or excessive exercise, and in central precious puberty due to increased BMI, where women tend to be affected more than men (179). Sex-specific differences have also been identified in a rodent model of the first gene identified by GWASs of pubertal timing, *Lin28*. Male *Lin28b* loss-of-function and male *let-7* gain-of-function, but not female, mice displayed alteration of pubertal timing, with later preputial separation (a marker of pubertal onset in male rodents) than in controls. In contrast, both male and female *Lin28a* gain-of-function mice displayed late onset of puberty. Taken together, these data point toward a complex system of regulation by *Lin28a*, *Lin28b*, and *let-7* in mice, in which *Lin28b* and *let-7* can impact both puberty and growth in a sex-specific manner, raising the possibility that this pathway may contribute to differential regulation of male and female growth and puberty in humans (180).

### Role of body mass

There has been a large body of research into the observed secular trend toward an earlier age of pubertal onset in the developed world. It is clear that nutrition plays an important role, with a positive correlation repeatedly demonstrated between age at puberty onset and childhood body size, particularly in girls. Lower age of both B2 development and menarche has been consistently associated with increased body mass. Higher BMI values were seen in early maturers and lower average BMI in late maturers in both white and Afro-Caribbean girls. This was quantified in a large cohort of children (n = 3650), with 1 BMI unit increase between the ages of 2 and 8 years being associated with a 0.11 year advancement in the timing of puberty in both sexes, as measured by peak height velocity (181). In contrast, undernutrition in females, for example in chronic disease or anorexia nervosa, can result in a delay in both the onset and tempo of puberty.

In boys the data are less consistent, with some studies having noted an earlier onset of puberty with greater adiposity and some with a later onset. In particular, more European studies have noted the former trend, whereas North American studies have more often shown the latter (14). More recent data from the United States have shown a far more complex relationship between fat mass and pubertal timing, with overweight status being associated with earlier pubertal onset but obesity being associated with later onset (182). These effects also varied between ethnic groups. It is hypothesized that greater BMI in boys leads to earlier pubertal timing up to the threshold at which obesity occurs. Obesity may lead to later pubertal timing due to suppression of the HPG axis or via adiposity leading to excess aromatase activity and increased conversion of testosterone to estrogen in boys.

The relationship between fat mass and pubertal timing is mediated, at least in part, through the permissive actions of the metabolic hormone leptin, a key regulator of body mass, produced from white adipose tissue. Serum leptin concentrations rise in early female puberty and are required for normal reproduction (183). Humans and mice lacking leptin (*Lep*^*ob/ob*^) or the leptin receptor (*LepR*^*db/db*^) fail to complete puberty and are infertile (184). The action of leptin in influencing GnRH secretion is not clear-cut. In males, leptin concentrations decrease during puberty. However, leptin does not act directly on GnRH neurons, as they do not express the LepR, but it appears to regulate GnRH neurons indirectly by its action on the hypothalamus via cells that are afferent to GnRH neurons (185). Such cells may include LEPR-expressing GABA neurons from the arcuate nucleus (ARC), or via cells that interact morphologically with them, at least in part via the action of nitric oxide (which is required for its action) and via kisspeptin/neuropeptide Y (NPY) neurons (186). Leptin administration has also been shown to increase mean LH levels and LH pulse frequency, as well as ameliorate the phenotypic features, in women with hypothalamic amenorrhea (103).

Additionally, NPY is involved in many CNS functions, including appetite control and reproduction. NPY modulates GnRH binding to anterior pituitary GnRH receptors and acts at the level of the median eminence to stimulate GnRH secretion from GnRH axon terminals, thus potentiating LH secretion in response to GnRH. NPY plays an important role in the metabolic control of fertility. A chronic increase in NPY tone inhibits LH and FSH, delays sexual maturation, and suppresses estrous cyclicity in rodents, but acute changes in NPY may have variable effects depending on the levels of sex steroid production. Evidence from primate studies suggests that NPY may have a contributory role in the break restraining the onset of puberty in primates (187).

*“Genetic mutations in >30 separate genes resulting in severely delayed or absent puberty have now been described….”*

Ghrelin and other gut-derived peptides may also form part of the mechanism by which energy homeostasis regulates reproductive development. Ghrelin is the endogenous ligand for the GH secretagogue receptor and is produced primarily by gastric mucosa. Ghrelin circulates in the blood and stimulates the secretion of GH, prolactin, and adrenocorticotropic hormone from the pituitary as well as hypothalamic control of food intake (188–190). Animal studies demonstrate that centrally or peripherally administered ghrelin reduces LH pulse frequency in ovariectomized rats and rhesus monkeys and decreases basal LH concentrations in intact rats and sheep (191). Both low birth weight and prematurity are associated with earlier onset of puberty, particularly in those children with a rapid increase in length or weight in the first 2 years of life. It remains unclear, however, whether childhood obesity, insulin resistance, excess androgens, or other factors may explain this association.

Despite this evidence, additional data point to a downward trend in the age of puberty onset that is independent of BMI (192). Moreover, although an ongoing strong secular trend toward earlier attainment of B2 has been recognized, the age of menarche in recent years, at least in Northern European studies, has not declined to the same extent. Indeed, as detailed above, some studies suggest that during the last decade the age of menarche and of completion of puberty in males in some populations has become skewed toward later ages. These data may imply that the increase in fat mass alone cannot explain this secular trend and suggest a role for factors that have an estrogen-like effect, without central activation of the HPG axis.

## Single-Gene Disorders Informing the Genetics of Pubertal Timing

Deletion mapping, homozygosity mapping in consanguineous kindreds with multiple affected members, targeted sequencing projects, and, more recently, next-generation sequencing approaches in patients with CHH and familial precocious puberty have led to the identification of some of the key regulators of the HPG axis. Genetic mutations in >30 separate genes resulting in severely delayed or absent puberty have now been described, and several important gene discoveries have been made recently in patients with CPP. These genes include *ANOS1* (OMIM 300836), FGF receptor 1 (*FGFR1*, OMIM 136350), *FGF8* (OMIM 600483), prokineticin 2 (*PROK2*, OMIM 607002), prokineticin 2 receptor (*PROK2R*, OMIM 607123), *CHD7* (OMIM 608892), NMDA receptor synaptonuclear signaling and neuronal migration factor (*NSMF*, OMIM 608137), *GNRH1* (OMIM 152760), GnRH receptor (GNRHR, OMIM 138850), *KISS1* (OMIM 603286), *KISS1R* (OMIM 604161), tacykinin 3 (*TAC3*, OMIM 162330), *TACR3* (OMIM 162332), semaphorin 3A (*SEMA3A*, OMIM 603961), SRY-box 10 (*SOX10*, OMIM 602229), IL-17 receptor D (*IL17RD*, OMIM 606807), FEZ family zinc finger 1 (*FEZF1*, OMIM 613301), WD repeat domain 11 (*WDR11*, OMIM 606417), AXL receptor tyrosine kinase (*AXL*, OMIM 109135), heparin sulfate 6-*O*-sulfotransferase 1 (*HS6ST1*, OMIM 604846), and *FGF17* (OMIM 603725) in HH (49, 51, 57, 193–205), and *MKRN3* (OMIM 603856), *KISS1R* (OMIM 604161), and *DLK1* (OMIM 176290) in CPP (162, 163, 206–208). These genes are involved in the control of GnRH neuronal migration and differentiation, GnRH secretion, or its upstream or downstream pathways.

### CHH

As described above in “Congenital HH” under “Differential Diagnosis of Delayed Puberty,” CHH may be sporadic or familial, although sporadic forms are found far more commonly. Familial CHH was initially considered a monogenic disorder with several modes of transmission described: X-linked recessive transmission, autosomal recessive transmission, autosomal dominant transmission, or transmission linked to an imprinting locus (Table 2). Although CHH cases are mainly sporadic, a detailed analysis of the pedigree may be informative when making the diagnosis.

#### Recessive transmission

Isolated CHH is transmitted as a recessive autosomal trait due to mutations in three pairs of neuropeptides/receptors. Loss-of-function mutations in *KISS1* (OMIM 603286) and its receptor *KISS1R* (OMIM 604161), as well as neurokinin B encoded by *TAC3* (OMIM 162330) and *TACR3* (OMIM 162332) have been described in informative families by linkage analysis. Mutations in *GNRH1* (OMIM 152760) and *GNRHR* (OMIM 138850) have been characterized by a candidate gene approach. An identical variant in both alleles suggests that parents are consanguineous but heterozygote composite variants are more frequent than homozygous variants in isolated CHH. The most frequent pathogenic variant in *GNRHR* (p.Gln106Arg) causes only a partial inactivation of the receptor activity that explains the relatively high frequency of this variant in the general population, with a minor allele frequency of 0.3%.

#### X-linked transmission

This mode of transmission has been observed only in KS (CHH with anosmia). *ANOS1* (OMIM 300836) is the only KS gene located on the X-chromosome reported to date. Boys with the *ANOS1* mutation are affected whereas females are unaffected but carriers.

#### Autosomal dominant transmission

Very rare variants in several autosomal genes have been reported in the dominant form of KS. For some of these genes, the transmission is caused by a monoallelic pathogenic mutation, whereas in others an additional genetic event is awaited to fully explain the phenotype. Dominant transmission of CHH is more frequent in KS than in isolated CHH. This mode of transmission is relatively surprising for a reproductive disorder causing infertility in adulthood. Such mutations may give rise to a partial phenotype or one with variable severity of gonadotropin deficiency. In the same family, isolated gonadotropic deficiency, KS, or isolated anosmia may be observed.

The molecular genetic understanding of CHH/KS has advanced tremendously in the past 20 years since the first KS gene, *ANOS1* (formerly *KAL1*), was identified by a positional cloning strategy in 1991. Most recent advances have stemmed from the screening of large cohorts of patients using next-generation sequencing techniques. These studies have led to the definition of two groups of genes with monoallelic variants: the first consists of genes in which rare monoallelic pathogenic variants have been confirmed by several independent studies; the second comprises genes in which monoallelic variants of unknown significance are more frequent in the patient group as compared with the control population (75). Associated clinical features such an ectrodactyly may be highly predictive of a pathogenic variant (209) [Table 3 (209–218)].

**Table 3.** Syndromes Associated With Pubertal Delay

	Phenotype	Genetic Defect
Prader–Willi syndrome (210)	Mental retardation, morbid obesity, hypotonia	Deletions within paternally imprinted 15q 11.2–12 region
Bardet–Biedl syndrome (211)	Mental retardation, obesity, retinitis pigmentosa, postaxial polydactyly	*BBS 1-11* (multiple loci) 20p12, 16q21, 15q22.3–23, 14q32.1
Biemond syndrome (212)	Iris coloboma, polydactyly, short stature	
CHARGE anomaly (213)	Coloboma, heart malformations, choanal atresia, growth retardation, genital anomalies and ear anomalies, HH, olfactory bulb aplasia, hypoplasia	*CHD7*
Adrenohypoplasia congenita (214)	Primary adrenal deficiency	*NR0B1*
Septo-optic dysplasia (215)	Small, dysplastic pale optic discs, pendular nystagmus, midline hypothalamic defect with diabetes insipidus, GH, ACTH, TSH, and LH/FSH deficiency, absent septum pellucidum	*HESX1*
Solitary median maxillary incisor syndrome (216)	Prominent midpalatal ridge	*SHH 7q3*
Börjeson–Forssman–Lehmann syndrome (217)	Mental retardation, gynecomastia, moderate short stature, truncal obesity	*PHF6*
Gordon Holmes syndrome (218)	Cerebellar ataxia, dementia, chorioretinopathy, anterior hypopituitarism	*RNF216/OTUD4*
		*PNPLA6*

For another group of patients, it is not possible to affirm the link between genotype and phenotype. The incomplete penetrance observed for these variants of unknown significance has led investigators to propose an oligogenic model of transmission. In this model, the probability of developing pubertal failure, and also the severity of disease, is due to the association of several rare variants in candidate genes. The frequency of this oligogenic transmission remains unknown. The fact that AAM and age at puberty are polygenic traits with hundreds of loci involved strongly supports the hypothesis that some cases of delayed puberty and even absent puberty could be transmitted as an oligogenic trait or even could be considered as part of a polygenic disorder. That CHH may be reversible in adulthood in a significant proportion of patients is probably related to this polygenic effect. Up to 20% of cases exhibit a spontaneous recovery of reproductive function (55, 201, 219), challenging the dogma that the CHH is (i) a life-long condition and (ii) a distinct entity from self-limited delayed puberty. We can hypothesize that there is a spectrum of pathology spanning between absolute GnRH deficiency and delayed puberty, with severe delayed puberty, partial CHH, and reversible CHH potentially lying along this spectrum. Genetic diagnosis in these patients may well help to unpick this clinical complexity. The greater mutational burden seen in patients with more severe disease, with those with KS or CHH carrying homozygous pathogenic mutations or multiple disease-causing mutations (digenicity or oligogenicity), is in support of this concept, although it is likely that some aspects of the genetic profiles of CHH and delayed puberty will be distinct (220).

**Figure 3.**
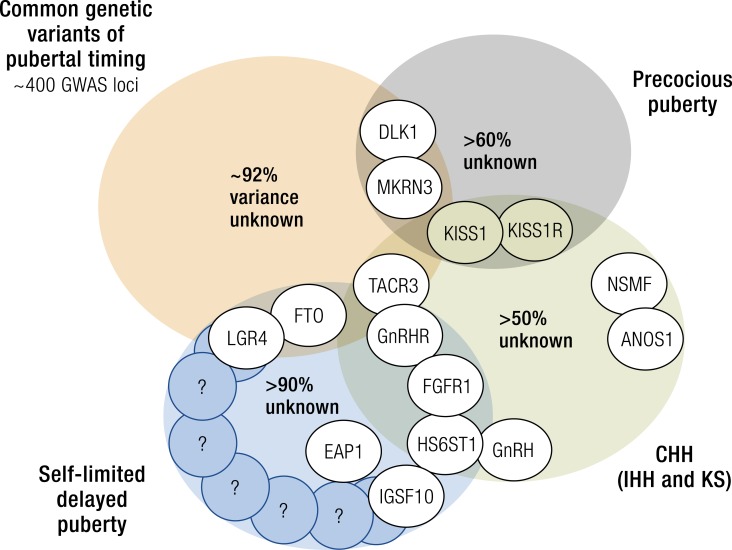
Established genetic basis of common genetic variants of pubertal timing, conditions of CHH (IHH and KS), precocious puberty, and delayed puberty and their overlap. Activating and inactivating mutations in Kiss1 and Kiss1R cause the opposite phenotypes, that is, precocious puberty and CHH, respectively. IHH, idiopathic HH.

These discoveries are critical in enhancing the understanding of the regulation of the HPG axis at puberty. Despite recent advances, with >40 genes linked to this disorder identified, the pathophysiological basis of CHH in ∼50% of individuals remains unclear (Fig. 3) (194). There is a greater or lesser degree of phenotypic variability depending on the gene involved, with nearly all mutations in *ANOS1* (OMIM 300836) leading to HH with anosmia (KS), whereas loss-of-function mutations in *FGFR1* (OMIM 136350) have been identified in pedigrees with KS, normosmic HH, and delayed puberty. Environmental factors may partially explain these variations, but it is increasingly clear that gene–gene interactions in CHH are an important phenomenon, and strategies to identify such digenic and even oligogenic inheritance in families are developing (201). In such kindreds, the pattern of those affected by disease may not conform to classic Mendelian inheritance dogma and bioinformatic filtering pipelines, and statistical modeling techniques will require modification to identify novel candidates for such gene–gene interactions.

### Development of the GnRH neuronal network

**Figure 4.**
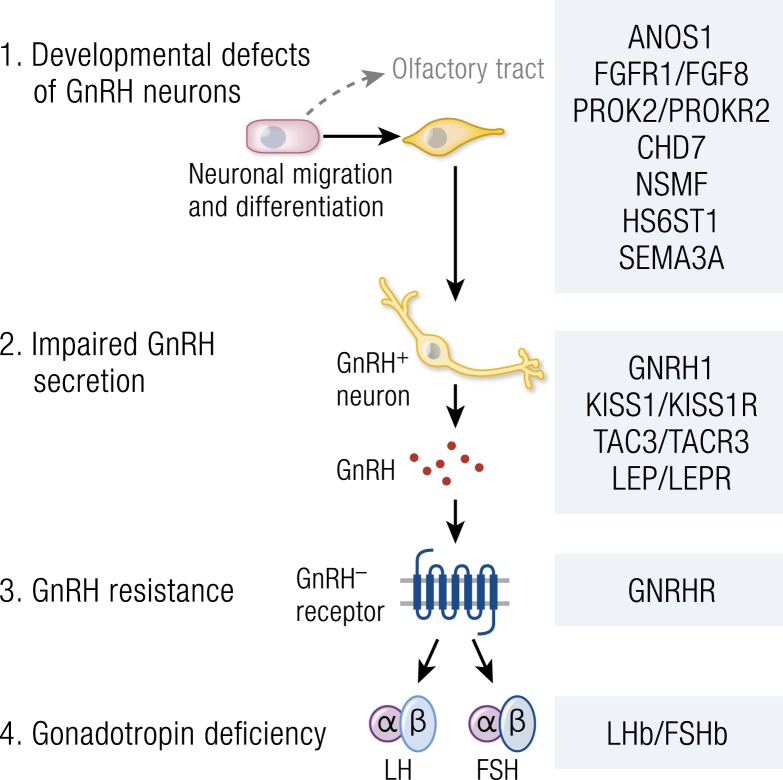
Mutations in single genes at many levels of the HPG axis can cause HH. [Copyright © 2012 Beate K, Joseph N, Nicolas de R, Wolfram K. Genetics of isolated hypogonadotropic hypogonadism: role of GnRH receptor and other genes. Int J Endocrinol. 2012;2012:147893. This is an open access article distributed under the Creative Commons Attribution License, which permits unrestricted use, distribution, and reproduction in any medium, provided the original work is properly cited.]

Different defects of the GnRH system have been described as resulting in pubertal failure (Fig. 4): (i) defects in GnRH synthesis, which mainly result from an abnormal migration of GnRH neurons from the olfactory placode toward the hypothalamus during the first trimester of fetal life; (ii) low GnRH secretion due to a defect of GnRH secretagogue bioactivity such as kisspeptin or neurokinin B; (iii) poor maturation of the GnRH neuronal network; and (iv) loss of function of GnRH itself, also known as a defect of the bioactivity of GnRH or its receptor (46).

The development of the HPG axis is exceptional in that GnRH neurons develop in metazoan embryos outside the CNS (221). The embryonic migration of GnRH neurons from nose to hypothalamus is key for the development of the neuroendocrine pathways that allow normal pubertal development (222). At the end of their journey, GnRH neurons dissociate from guiding axons to disperse into their final positions of the septohypothalamic region, including the medial septum, the diagonal of Broca, and the preoptic area of the hypothalamus (223). GnRH neurons extend their neurites to the median eminence under the control of mostly unknown factors (224). FGFR1 signaling has been shown to be important for this process of axon extension, with reduced projections to the median eminence in transgenic mice expressing a dominant-negative FGF receptor in GNRH1 neurons (225).

**Figure 5.**
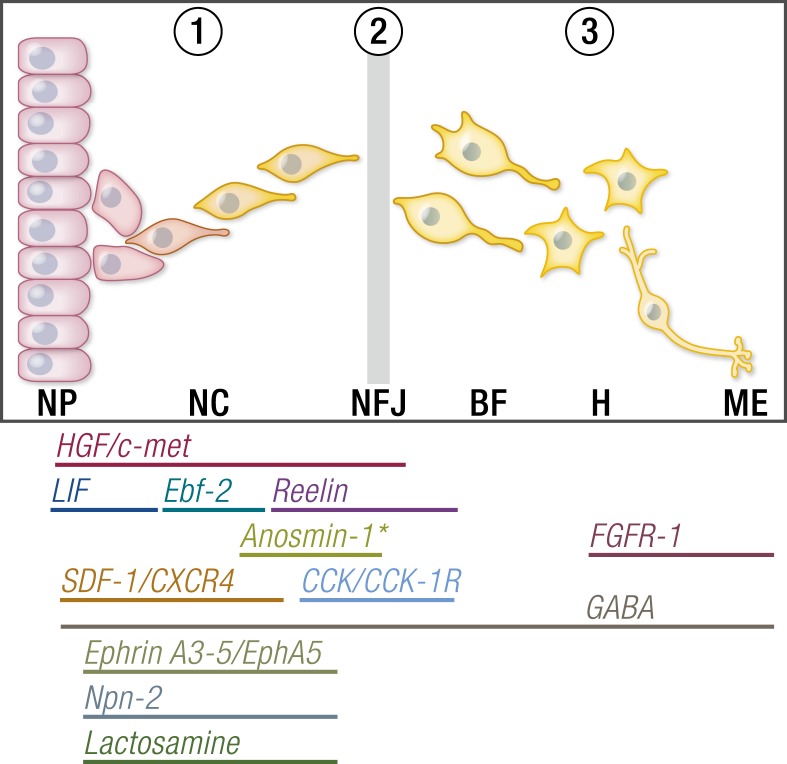
Factors that affect the migration of GnRH neurons through the three compartments. The illustration shows the movement of GnRH neurons from their origin in the nasal placode (NP), through the nasal compartment (NC), and their deflection at the level of the nasal–forebrain junction (NFJ) as they progress toward the basal forebrain (BF). Their migration terminates in the hypothalamus (H) from where they project to the median eminence (ME). Factors that have been shown to affect GnRH neurons at different stages of their journey are shown below. CCK, cholecystokinin; CXCR4, CXC chemokine receptor type 4; Ebf-2, early B-cell factor 2; HGF, hepatocyte growth factor, LIF, leukemia inhibitory factor; Npn-2, neoplastic progression 2; SDF1, stromal cell–derived factor 1. [Republished with permission of Oxford University Press, from Cariboni A, Maggi R, Parnavelas JG. From nose to fertility: the long migratory journey of gonadotropin-releasing hormone neurons. Trends Neurosci. 2007;30(12):638–644. Permission conveyed through Copyright Clearance Center, Inc.]

Migratory GnRH neurons receive a plethora of guidance and movement-inducing messages during this journey, which are likely to be distinct depending on the stage of their migration (Fig. 5) (222). Signals may act directly or indirectly through the extension of olfactory axons, as disruption of the nerve tract “scaffolds” themselves can disrupt GnRH migration (226). The molecular signals involved include those controlling cell–cell interactions [membrane receptors (*e.g.*, neuropilin-2), adhesion molecules (*e.g.*, NCAM), extracellular matrix molecules (*e.g.*, heparin sulfotransferases), cytokines (*e.g.*, leukemia inhibitory factor, hepatocyte growth factor), and transcription factors (*e.g.*, Ebf-2), as well as both chemoattractants and chemorepellents (*e.g.*, Reelin) (227–231]. Gradients of chemokines (*e.g.*, SDF1, also known as CXCL12) may be particularly important for promoting movement of GnRH neurons (226, 232). This combination of factors has a high degree of redundancy, which is necessary given the crucial role that this GnRH neural network plays in reproductive function.

The whole process of migration involves a few hundred neurons per hemisphere in mice (several thousand in primates or humans) (222). The absolute number of GnRH neurons required for pubertal development is not known but there appears to be a degree of redundancy in the system (233). Rodent studies suggest that ∼12% of the GnRH neuron population is sufficient for pulsatile gonadotropin secretion and puberty onset, whereas between 12% and 34% is required for cyclical control in adult female mice. Additionally, adult Reeler mice have significantly fewer GnRH neurons in the hypothalamus and display a phenotype of delayed pubertal maturation and low fertility (228).

*ANOS1* (OMIM 300836) encodes anosmin-1, an extracellular matrix protein that regulates axonal path finding and cellular adhesion. Anosmin-1 promotes branching of olfactory bulb neurons. Subjects with *ANOS1* loss-of-function mutations have arrest of both GnRH neurons and olfactory bulb neurons at the cribriform plate (234). It is not yet clear whether the effects of anosmin-1 are limited to the development of olfactory neurons, or whether it has an additional chemotactic influence on GnRH neurons (235). Although no mouse model is available, fish and nematode studies and *in vitro* work have further elucidated the role of *ANOS1* (236).

### Upstream control of GnRH neuronal function

The master controller of the GnRH neuronal pulse generator controlling the onset of puberty is still a subject of active research (237). GnRH is secreted in a pulsatile fashion into the hypothalamic–pituitary–portal system by nerve terminals located in the median eminence to reach the anterior pituitary where it stimulates the gonadotropins LH and FSH secretion by pituitary gonadotrope cells. GnRH is released in episodic boluses, and the secretion of GnRH pulses is synchronized between GnRH neurons, such that they integrate their firing rates to generate an appropriate burst of GnRH release into the portal system (238). This synchrony is a complex process involving spontaneous electrical activity of the neurons, calcium and cAMP signaling, autocrine regulation through the GnRH receptor, and regulation through other cell membrane receptors on these neurons.

**Figure 6.**
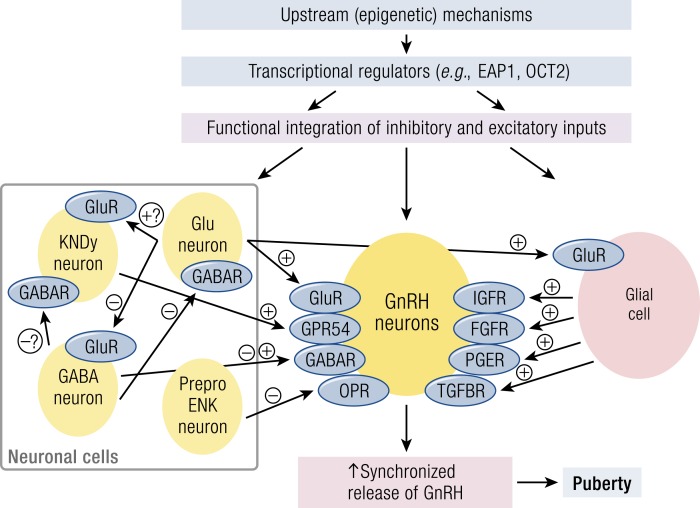
Genetic regulators in the trans-synaptic and glial control of GnRH neurons during puberty. + Represents an activating signal, whereas − represents a repressing signal. [Adapted with permission from Ojeda SR, Lomniczi A, Mastronardi C, *et al.* Minireview: the neuroendocrine regulation of puberty: is the time ripe for a systems biology approach? Endocrinology. 2006;147(3):1166–1174.]

The theory that the pulse generator is an intrinsic property of GnRH neurons has been mostly rejected, given evidence derived from models such as retrochiasmatic rat hypothalamic explants, which contain few if any GnRH cell bodies, but continue to exhibit pulsatile GnRH release in culture (239). Retrograde tracing studies in mice have shown that GnRH neurons are subject to a complex neuronal network of inputs from many regions of the brain, including hypothalamic nuclei, the brainstem, limbic system, basal ganglia, and motor and sensory circuits (240). GnRH release is coordinated through a balance of inhibitory and excitatory neuronal and glial inputs (241) (Fig. 6).

Among various regulators of GnRH neurons, kisspeptins and neurokinin B are essential inputs. This “KNDy” model of pulse generation has key neurons in the ARC as responsible for coordinating pulse generation through the peptides kisspeptin, neurokinin B, and dynorphin (187). Kisspeptin, an excitatory neuropeptide, was identified as a vital permissive factor in puberty onset by the discovery of patients with GnRH deficiency with loss-of-function mutations in *KISS1R* (OMIM 604161), previously known as *GPR54* (242, 243). Mice with knockout of *Kiss1r* were simultaneously discovered to be infertile despite anatomically normal GnRH neurons and normal hypothalamic GnRH levels (243). Their phenotype can be rescued by exogenous delivery of GnRH. *Kiss1* (OMIM 603286) knockout mice also have a phenotype consistent with normosmic GnRH deficiency.

Kisspeptins are synthesized by hypothalamic neurons that are in close contact with GnRH neurons. Most GnRH neurons express the kisspeptin receptor, and kisspeptin neurons express steroid receptors including estrogen receptor *α*, the progesterone receptor, and the androgen receptor (244). These neurons are the main relay for the negative and positive feedback of steroid hormones on the gonadotropic axis (245). Axonal ends of kisspeptin neurons project to the GnRH neuron cell body in the *organum vasculosum laminae terminalis*, but also to the median eminence where they are in apposition with the extremities of GnRH neurons (246). Kisspeptin neurons are located outside the blood–brain barrier and are therefore directly in contact with peripheral hormones.

Kisspeptin thus signals directly to GnRH neurons to control pulsatile GnRH release. It is upregulated in both primates and mice in the peripubertal period and its administration in prepubertal rodents advances the onset of puberty (247). Kisspeptin also appears to be downregulated in functional amenorrhea, suggesting its role as a mediator of the action of environmental factors such as nutritional status and emotional well-being on puberty and reproductive capacity. Additionally, kisspeptin has been shown to be an important neuroendocrine regulator of ovulation (248). Kisspeptin signaling is an important element of both positive and negative feedback loops in the HPG axis. Although kisspeptin has been identified as a pivotal upstream regulator of GnRH neurons, whether it is the key factor in triggering the onset of puberty remains unclear (248, 249).

An additional excitatory neuropeptide, neurokinin B, has been implicated in the upstream control of GnRH secretion (250). Identification of this pathway was also via discovery of loss-of-function mutations in *TAC3* (OMIM 162330), encoding neurokinin B, and its receptor *TACR3* (OMIM 162332) in patients with normosmic HH and pubertal failure (198, 251). Kisspeptin neurons located in the ARC, which synthesize neurokinin B and dynorphin A, are known as KNDy neurons. Both *KISS1* and *TAC3* expression in the ARC is downregulated by estrogen, and these neurons are considered as the relay of the negative feedback of steroid hormones on the gonadotropic axis (252). KNDy neurons express the neurokinin receptor, NK3R, suggesting that autocrine and paracrine loops control GnRH release (253). Dynorphin inhibits the release of GnRH and together these peptides are thought to play a fundamental role in the GnRH pulse generator.

Another RF-amide related peptide (RFRP, OMIM 616984), the mammalian ortholog of the avian peptide gonadotropin-inhibiting hormone (*GnIH*), has emerged as a further inhibitory regulator of the gonadotropic axis by directly controlling GnRH neurons (254) and via a subset of kisspeptin neurons (255). GnIH plays a crucial role in the inhibitory regulation of the HPG axis in several species. In addition to neuropeptides, several neurotransmitters participate in the control of the GnRH network. In the ARC, GABA and glutamate control GnRH neuronal excitability (256). In female rats, glutamine synthase is downregulated and glutamate dehydrogenase becomes more abundant in the hypothalamus at puberty, both leading to increased availability of glutamate (257). Glutamate antagonists are potent stimulators of GnRH secretion, and administration to prepubertal primates can stimulate the onset of puberty. The GABA neural network is quite complex because some of these neurons will have a direct effect on GnRH neurons and others will act on interneurons. The inhibitory role of GABAergic neurotransmission in restraining the initiation of puberty has been clearly shown in primates but is more ambiguous in rodents (258). GABAergic signaling pathways are likely to be important in the stress-induced suppression of LH.

The synchronous pulsatile secretion of GnRH is also controlled through the activation of neuronal–glial signaling pathways (259, 260). Glial inputs appear to be predominantly facilitatory and consist of growth factors and small diffusible molecules, including TGF*β*1, IGF-1, and neuregulins, that directly or indirectly stimulate GnRH secretion (261). First, glial cells in the median eminence regulate GnRH secretion by production of growth factors acting via receptors with tyrosine kinase activity. FGF signaling is required for GnRH neurons to reach their final destination in the hypothalamus (225), as well as for GnRH neuronal differentiation and survival (262). Additionally, GnRH neuron secretory activity is facilitated by IGF-1 and by members of the epidermal growth factor family such as neuregulin 1*β* (261, 263). Second, plastic rearrangements of glia–GnRH neuron adhesiveness mediated by soluble molecules such as neuronal cell adhesion molecule and synaptic cell adhesion molecule coordinate the controlled delivery of GnRH to the portal vasculature, a process that is also subject to sex steroid regulation (264). These neural and neuroendocrine regulators are ultimately responsible for fine-tuning the pulsatile secretion of GnRH into the hypophysial portal circulation (265) to control the next level of this hierarchical regulatory cascade, that is, the gonadotrope cells of the anterior pituitary gland.

Puberty is marked by the change of the balance of GABA–glutamate signaling in the brain (256). This is associated with a higher dendritic spine density and a simplification of the dendritic architecture of GnRH neurons. The timing of puberty is also correlated to an increase of the kisspeptin signaling in the hypothalamus that is due to an increase of kisspeptin synthesis as well as an increased responsiveness of GnRH neurons to kisspeptin stimulation. Although mainly described in mice, this paradigm is probably true in monkeys as well and relatively well conserved during evolution (266).

The mechanisms responsible for the increased biosynthesis of kisspeptins at the end of the juvenile period in the hypothalamus remain unknown. Data pointing to hypothalamic regulation via a hierarchical network of genes (Fig. 6) have come mainly from a systems biology approach (267) and animal models (187) with little data from human subjects. Candidate transcriptional regulators identified by these approaches include *Oct-2* (OMIM 164176), *Ttf-1* (OMIM 600635), and enhanced at puberty 1 (*Eap1*, OMIM 611720). *Oct-2* is a transcriptional regulator of the POU-domain family of homeobox-containing genes. *Oct-2* mRNA is upregulated in the hypothalamus in juvenile rodents; blockage of Oct-2 synthesis delays age at first ovulation, and hypothalamic lesions that induce precocious puberty (*e.g.*, hamartomas) activate Oct-2 expression (268). *Ttf-1* is another homeobox gene that enhances GnRH expression (269). *Ttf-1* expression is increased in pubertal rhesus monkeys (270). *Eap1* mRNA levels also increase in the hypothalamus of primates and rodents during puberty, *Eap1* transactivates the *GnRH* promoter, and Eap1 knockdown with small interfering RNA caused delayed puberty and disrupted estrous cyclicity in a rodent model (271–275). *Eap1* gene expression itself seems to be under dual transcriptional regulation by *Ttf-1* activation and *Yy1* (OMIM 600013) and *Cux1* (OMIM 116896) repression (276).

Recent data have highlighted the importance of a transcriptional repressive program that controls the expression of *Kiss1.* The intervention of the polycomb complex proteins EED (OMIM 605984) and Cbx7 (OMIM 608457) in the transcriptional repression of *Kiss1* (OMIM 603286) is thought to be an important mechanism preventing the premature initiation of puberty (277). The expression of these genes in the prepubertal period decreases with increasing methylation of their promoters. The binding of EED on the *Kiss1* promoter decreases at puberty. The inhibition of the repression of *Kiss1* is also correlated to a decrease in the expression of transcription factors with zinc finger motifs. Importantly, the loss of these polycomb complex proteins from the promoter is accompanied by a reorganization of the chromatin status and changes in histone methylation (278).

Additionally, the role of noncoding RNAs as epigenetic modulators of pubertal timing has been illustrated in a mouse model of GnRH-specific impaired miRNA synthesis. A key pair of miRNAs [miR-200 (OMIM 612090-2) and mIR-155 (OMIM 609337)] has been proposed to control the expression of Gnrh (OMIM 152760), Zeb-1 (OMIM 189909), and Cebpb (OMIM 189965), with the latter two being both potent transcriptional repressors of Gnrh. At puberty there is a switch in activity with a decrease in the transcriptional activation of the GnRH gene and an increase in Zeb-1 and Cebpb, leading to the rise of *GNRH1* synthesis, which occurs during the juvenile period in GnRH neurons (169). The increase of kisspeptin expression in the hypothalamus therefore results from a complex network of transcription factors acting as repressors and activators of *Kiss1* and *GNRH1* transcription, with these in turn being under the influence of a number of different epigenetic mechanisms, including DNA methylation, histone modification, and noncoding RNAs (169, 278–280). The plasticity of GnRH neurons to kisspeptin stimulation is more obscure. The kisspeptin receptor is a G-protein–coupled receptor coupled to G_q/11_ protein. The regulation of G-protein–coupled receptor activity is multifactorial (281). G-protein–coupled receptors can undergo acute but also chronic desensitization through multiple intracellular signaling pathways and regulatory proteins. This regulation is thought to be associated with the maturation status of GnRH neurons.

The concept that puberty results from the disappearance of gonadotropic axis repression is also supported by the description of loss of function mutations of *MKRN3* (OMIM 603856) in familial CPP (162, 282, 283). This gene encodes makorin ring finger protein 3, a zinc finger protein containing a C3HC4 motif termed a RING domain associated with E3 ubiquitin ligase activity. MKRN3 is likely to have an inhibitory effect on the GnRH network because its expression in the ARC decreases in mice between birth and weaning and circulating levels in humans decline at puberty onset (284, 285). Where *MKRN3* might be placed in the hierarchical network of genes controlling kisspeptin has yet to be determined.

Puberty must thus be considered as the output of a neurodevelopmental program, which shares several features with the postnatal development of other neuronal functions. The specificity of this program resides in its timing, which is determined by genetic factors dependent on the hormonal status and modulated by environmental factors. It is not surprising that delayed puberty and even absent pubertal development are not infrequent pathologies observed in humans.

### Downstream pathways of GnRH action

Loss-of-function mutations within the GnRH receptor are the most frequent cause of autosomal recessive CHH, accounting for 16% to 40% of patients. Mutations have been found within the extracellular, transmembrane, and intracellular domains of the receptor leading to impaired GnRH action (286). LH and FSH are glycoprotein hormones encoded by a common *α*-subunit gene and a specific *β*-subunit gene. Mutations of the *β*-subunits of LH or FSH are rare causes of HH (287, 288). Females with inactivating mutations of *LHβ* (OMIM 152780) present with onset of normal puberty and normal or late menarche followed by infertility due to lack of ovulation. Males with inactivating mutations of the *LHβ*-subunit have absent pubertal development with Leydig cell hypoplasia leading to testosterone deficiency and azoospermia. Individuals with inactivating *FSHβ* (OMIM 136530) mutations present with incomplete pubertal development and primary amenorrhea in females and azoospermia in males (289).

## Genetics of Self-Limited Delayed Puberty

Self-limited delayed puberty segregates within families with complex patterns of inheritance, including autosomal dominant, autosomal recessive, bilineal, and X-linked, although sporadic cases are also observed. Most families display an autosomal dominant pattern of inheritance (with or without complete penetrance) (290–292). Fifty percent to 75% of subjects with self-limited delayed puberty have a family history of delayed pubertal onset (291). Self-limited delayed puberty is not sex specific, as near equal sex ratios among family members are seen (292). Although a predominance of males presenting with the condition has been noted, this may be a consequence of referral bias.

The neuroendocrine pathophysiology and its genetic regulation remain unclear in most patients with delayed puberty (293, 294). Analysis of self-limited delayed puberty families is complicated by the fact that this phenotype represents the tail of a normally distributed trait within the population, so it is expected that variants that govern the inheritance of this condition may also be present in the general population at a low level. Thus, the absence of these variants in population databases cannot be used as an exclusion criterion during filtering of sequencing data. Instead, a comparison of prevalence of such variants must be made to identify those that are enriched in patients compared with the general population.

### Overlap with CHH

In view of the possible overlap between the pathophysiology of delayed puberty and conditions of GnRH deficiency, a few groups have specifically examined the contribution of mutations in CHH genes to the phenotype of self-limited delayed puberty. Studies in cohorts of kindreds with CHH have previously described mutations in *HS6ST1*, *FGFR1*, and more recently in klotho beta (*KLB*) in a small number of CHH individuals and their relatives with delayed puberty (56, 204, 295). Most recently, a comparative study of the frequency of mutations in 24 GnRH deficiency genes between probands with CHH and those with self-limited delayed puberty found a significantly higher proportion of mutations in the CHH group (51% of CHH probands vs 7% of delayed puberty probands, *P* = 7.6 × 10^−11^), with a higher proportion of oligogenicity in the CHH group, suggesting mostly distinct genetic profiles in these two conditions (220). Mutations in KS genes such as *ANOS1* and *NSMF* have not to date been identified in pedigrees with delayed puberty.

In studies specially examining delayed puberty cohorts, variants in several CHH genes, including *GNRHR*, *TAC3*, *TACR3*, *IL17RD*, and *SEMA3A*, have been identified by whole-exome sequencing (296). However, these variants have not been tested *in vitro* or *in vivo* for pathogenicity, or investigated for segregation with trait within pedigrees, and thus they may represent an overestimation.

Using whole-exome and targeted resequencing methods, a deleterious mutation in the CHH gene *HS6ST1* was recently found as the likely causal factor for self-limited delayed puberty in one extended pedigree from the same large cohort of patients with familial delayed puberty (297). The mutation was carried by six family members from three generations, all with typical features of self-limited delayed puberty. The proband had spontaneous onset of puberty at 14.3 years. Parallel studies in a murine model corroborated heterozygous *Hs6st1* deficiency as a cause of delayed pubertal timing without compromised fertility. Thus, *Hs6st1*^+/−^ mice were born at normal Mendelian ratios without obvious defects in GnRH neuron or testes development, but females showed delayed vaginal opening, a marker of pubertal onset in female rodents. GnRH deficiency was excluded both in male and female *Hs6st1*^*+/−*^ mice by reproductive competence and by normal spermatogenesis in males.

No abnormalities in olfactory bulb morphology, GnRH neuron number in the medial preoptic area, or in GnRH neuron innervation of the median eminence were detected in *Hs6st1*^*+/−*^ mice. Instead, *Hs6st1* expression in the ARC and paraventricular nucleus, where kisspeptin neurons and tanycytes modulate GnRH secretion and function (298, 299), raises the possibility that HS6ST1 haploinsufficiency affects the regulation of GnRH neuron activity or other relevant downstream pathways. These findings suggest that perturbations in a single allele of a gene regulating the HPG axis are sufficient to cause self-limited delayed puberty. In contrast, available evidence supports that more deleterious alterations in the same gene, or in combination with additional genes, are required to cause more severe CHH phenotypes (201).

**Figure 7.**
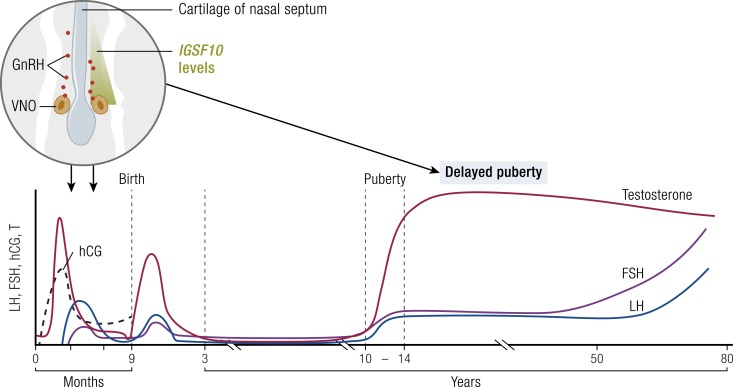
Schematic of the mechanism by which *IGSF10* mutations lead to delayed puberty. Reduced levels of *IGSF10* expression during embryogenesis in the corridor of nasal mesenchyme from the vomeronasal organ (VNO) to the olfactory bulbs result in delayed migration of GnRH neurons to the hypothalamus. This presents for the first time in adolescence as a phenotype of delayed puberty due to abnormalities of the GnRH neuronal network.

In support of this, using a similar approach with whole-exome and targeted resequencing methods, two pathogenic mutations in immunoglobulin superfamily member 10 (*IGSF10*) have been implicated as the causal factor for late puberty in six unrelated families from a large Finnish cohort with familial self-limited delayed puberty (300). A further two rare variants of unknown significance were identified in four additional families from the cohort. Mutations in *IGSF10* appear to cause a dysregulation of GnRH neuronal migration during embryonic development (Fig. 7), which presents in adolescence as delayed puberty without previous constitutional delay in growth. An intact GnRH neurosecretory network is necessary for the correct temporal pacing of puberty. Pathogenic *IGSF10* mutations leading to disrupted IGSF10 signaling potentially result in reduced numbers, or mis-timed arrival, of GnRH neurons at the hypothalamus, producing a functional defect in the GnRH neuroendocrine network. With this impaired GnRH system there would follow an increased “threshold” for the onset of puberty, with an ensuing delay in pubertal timing. *IGSF10* loss-of-function mutations were also discovered in patients with a hypothalamic amenorrhea-like phenotype. Although loss-of-function mutations in *IGSF10* were enriched in patients with CHH, these mutations did not alone appear sufficient to cause the phenotype of full GnRH deficiency, in view of lack of complete segregation with trait. These findings represent a fetal origin of self-limited delayed puberty, and they suggest a potential shared pathophysiology between delayed puberty and other forms of functional hypogonadism such as hypothalamic amenorrhea.

*“The mystery of what induces the dormancy of the HPG axis after mini-puberty and what triggers the release of this “puberty brake” remains unanswered.”*

As loss-of-function mutations within the GnRH receptor are one of the most frequent causes of CHH, the *GNRHR* gene has been repeatedly sequenced in patients with self-limited delayed puberty (286). However, only a small number of pathogenic mutations in this gene have been identified in this cohort of patients. A homozygous partial loss-of-function mutation in *GNRHR* was found in two brothers, one with self-limited delayed puberty and one with CHH (301), and a further heterozygous mutation was found in one male with self-limited delayed puberty (302). Overall, the current picture indicates that the genetic background of CHH and delayed puberty may be largely different, or shared by as yet undiscovered genes (302).

### Overlap with common genetic variants of pubertal timing

Rare heterozygous variants in *FTO* have been identified in pedigrees with self-limited delayed puberty associated with extreme low BMI and maturational delay in growth in early childhood (303). Notably, mice that were heterozygous for *FTO* gene knockout displayed significantly delayed timing of puberty without marked reduction in body mass. *FTO* was the first obesity-susceptibility gene identified through GWASs and continues to be the locus with the largest effect on BMI and obesity risk (304). *FTO* appears to exert this effect via influence on food intake regulation rather than physical exertion (305), although its actions may be complex (306). The finding of the importance of Iroquois homeobox 3 (*IRX3*), a second gene found at the same GWAS locus, in influencing BMI threw into doubt the primary role of *FTO* (307). However, the body of evidence behind *FTO*, in particular the results from *FTO*-knockout mice (308) and *in vitro* studies showing that FTO expression is regulated by essential amino acids and that it couples amino acid levels to mTORC1 signaling (309), have reinforced *FTO* as a major player in the regulation of body mass, although it may also act in concert with other genes in the nearby region to exert effects on body weight. A novel concept in the analysis of GWAS data is that a number of genes in any one identified region may play an important role in a particular phenotype. There is evidence that mTOR plays a central role in the coupling of energy balance and HPG axis activation via modulation of hypothalamic expression of Kiss1 (310). Blockade of mTOR caused delayed vaginal opening in rodents with blunting of the positive effects of leptin on puberty onset in food-restricted females. It remains to be determined whether the effect of *FTO* on pubertal timing in self-limited delayed puberty is mediated via effects on body mass, via mTOR signaling, or both.

Roles for other genes connected with regulation of body mass have not been clearly demonstrated in delayed puberty. *α*-MSH signaling via MC3/4 receptors, acting to increase Kiss1 expression and mediate the permissive effects of leptin on puberty, has also been implicated recently as an important element in the metabolic control of puberty (311). As mentioned above, ghrelin and other gut-derived peptides may also form part of the mechanism by which energy homeostasis regulates reproductive development (190, 312). A small cohort of 31 patients was analyzed for mutations in the ghrelin receptor, or GH secretagogue receptor (*GHSR*), and 5 patients were found to have point mutations in this gene (313).

### Gene defects relevant to self-limited delayed puberty but not to CHH

Loss-of-function mutations in a member of the immunoglobulin superfamily, immunoglobulin superfamily member 1 (*IGSF1*), have been identified in patients with X-linked central hypothyroidism (314). Male patients with *IGSF1* mutations have a late increase in testosterone levels with a delayed pubertal growth spurt. However, pathogenic mutations in *IGSF1* have not been conclusively found in patients with isolated delayed puberty (315).

A very recent discovery is of the first human *EAP1* mutations that appear to be causal for self-limited delayed puberty in two families (316). The two affected probands had classic clinical and biochemical features of self-limited delayed puberty presenting at more than 15.5 years but spontaneous pubertal development by the age of 18 years without testosterone therapy excluding CHH. Two highly conserved variants in *EAP1* were identified via whole-exome sequencing—one in-frame deletion and one rare missense variant. Using a luciferase reporter assay, *EAP1* mutants showed a reduced ability to trans-activate the GnRH promoter compared to wild-type *EAP1*, owing to reduced protein levels caused by the in-frame deletion and subcellular mislocation caused by the missense mutation. This study also demonstrated by chromatin immunoprecipitation that EAP1 binding to the GnRH1 promoter increases in monkey hypothalamus at the onset of puberty.

## Perspectives

Puberty is the period of sexual maturation when the transition to adult reproductive capacity, body composition, and adult height occurs. Its biological control is complex and involves multiple endocrine systems interacting in an ordered and progressive pattern. The origins of these biological processes begin early in fetal life, and fetal and neonatal developments are important to allow puberty to occur in an ordered and timely fashion in adolescence. The mystery of what induces the dormancy of the HPG axis after mini-puberty and what triggers the release of this “puberty brake” remains unanswered. There are multiple influences on the timing of puberty in the general population, and a wide variety of genetic, epigenetic, and environmental factors affecting different aspects of the HPG axis at different time periods in fetal and postnatal life may result in delayed and disordered puberty.

With respect to clinical practice, delayed puberty is a frequent problem presenting to the pediatric endocrinologist. Whereas the most common underlying condition in delayed onset of puberty is self-limited (or constitutional) delayed puberty, other pathological causes may underlie these conditions and must be excluded. In particular, distinguishing between self-limited delayed puberty and permanent HH in adolescence remains difficult, but the latter can be diagnosed in infancy when the suspicion arises. Management of adolescents with pubertal disorders is dependent on the underlying cause. Expectant observation is appropriate in benign variants of puberty and in those with milder forms of delayed puberty who are not predicted to have negative outcomes from their condition. Treatment of more significant precocious or delayed puberty involves medication to block or induce activity of the HPG axis, although more complex and involved management is required in patients with permanent hypogonadism. Achievement of fertility in patients with central hypogonadism requires therapy with gonadotropins.

Therefore, although genetic testing may inform diagnosis of associated syndromic features, natural history of the condition, and inheritance in family members, it may also represent a future diagnostic tool for the differentiation of the conditions of self-limited delayed puberty and GnRH deficiency. Rapid and efficient diagnosis of patients in the clinic would represent a huge leap forward in patient care and a likely significant economic advantage.

## Conclusions

This review serves to highlight the fascinating heterogeneity of genetic defects resulting in delayed and disordered puberty, with a special emphasis on a condition called self-limited delayed puberty. Investigation of the genetic control of delayed puberty is complicated by the facts that this trait is not rare and that the phenotype is likely to represent a final common pathway, with a variety of different pathogenic mechanisms affecting the release of the puberty “brake.” Although our understanding of the highly complex mechanisms underlying this biological network remains imperfect, results to date have demonstrated the importance of defects in GnRH neuronal development and function, including GnRH receptor and LH/FSH abnormalities, transcriptional regulation of the HPG axis, and metabolic and energy homeostatic derangements, in the pathogenesis of delayed puberty. Most recently the complex network of transcription factors acting as repressors and activators of the kisspeptin and GnRH system, as well as their higher control by a number of different epigenetic mechanisms, including DNA methylation, histone modification, and noncoding RNAs, has sparked great interest. Thus, genetic regulators of pubertal timing can exert their influence from early fetal life via development of the GnRH network, through prenatal and postnatal development and onwards to middle childhood, and potentially mediate the effects of environmental influences on pubertal timing.

In summary, the considerable progress in understanding the genetic control of puberty during the last 10 years has been a success story of basic research in neuroendocrinology but has also been translated into clinical practice to allow a better understanding of the etiopathogenic mechanisms of disordered pubertal development and, in some cases, to enable diagnostic genetic testing and counseling.

## Abbreviations:

AAM: age at menarche
AMH: anti-Müllerian hormone
ANOS1: anosmin 1
ARC: arcuate nucleus
BMI: body mass index
CHARGE: coloboma, heart malformations, atresia of the choanae, retardation of growth and development, genital anomalies, and ear anomalies (auditory and vestibular)
CHD7: chromodomain helicase DNA binding protein 7
CHH: congenital HH
CNS: central nervous system
CPP: central precocious puberty
DLK1: delta-like noncanonical Notch ligand 1
Eap1: enhanced at puberty 1
EDC: endocrine-disrupting chemical
FGF: fibroblast growth factor
FGFR1: FGF receptor 1
FTO: fat mass and obesity-associated protein
GABA: *γ*-aminobutyric acid
GWAS: genome-wide association study
GNRHR: GnRH receptor
HH: hypogonadotropic hypogonadism
HPG: hypothalamic–pituitary–gonadal
HS6ST1: heparin sulfate 6-*O*-sulfotransferase 1
IGSF1: immunoglobulin superfamily member 1
IGSF10: immunoglobulin superfamily member 10
IL17RD: IL-17 receptor D
KISS1: kisspeptin 1
KISS1R: KISS1 receptor
KS: Kallman syndrome
LEPR: leptin receptor
MKRN3: makorin ring finger protein 3
NHANES III: Third National Health and Nutrition Examination Survey
NPY: neuropeptide Y
NR0B1: nuclear receptor subfamily 0 group B member 1
NR5A1: nuclear receptor subfamily 5 group A member 1
Oct-2: organic cation transporter 2
PWS: Prader–Willi syndrome
RA: retinoic acid
SEMA3A: semaphorin 3A
TAC3: tachykinin 3
TACR3: tachykinin receptor 3
Ttf-1: thyroid transcription factor-1

## References

[1] Marshall WA, Tanner JM Variations in the pattern of pubertal changes in boys. Arch Dis Child. 1970;45(239):13–23. 5440182 10.1136/adc.45.239.13 PMC2020414

[2] Marshall WA, Tanner JM Variations in pattern of pubertal changes in girls. Arch Dis Child. 1969;44(235):291–303. 5785179 10.1136/adc.44.235.291 PMC2020314

[3] Palmert MR, Boepple PA Variation in the timing of puberty: clinical spectrum and genetic investigation. J Clin Endocrinol Metab. 2001;86(6):2364–2368. 11397824 10.1210/jcem.86.6.7603

[4] Sklar CA, Kaplan SL, Grumbach MM Evidence for dissociation between adrenarche and gonadarche: studies in patients with idiopathic precocious puberty, gonadal dysgenesis, isolated gonadotropin deficiency, and constitutionally delayed growth and adolescence. J Clin Endocrinol Metab. 1980;51(3):548–556. 6447708 10.1210/jcem-51-3-548

[5] Wierman ME, Beardsworth DE, Crawford JD, Crigler JF Jr, Mansfield MJ, Bode HH, Boepple PA, Kushner DC, Crowley WF Jr Adrenarche and skeletal maturation during luteinizing hormone releasing hormone analogue suppression of gonadarche. J Clin Invest. 1986;77(1):121–126. 2935557 10.1172/JCI112265 PMC423317

[6] Largo RH, Prader A Pubertal development in Swiss girls. Helv Paediatr Acta. 1983;38(3):229–243. 6618891

[7] Largo RH, Prader A Pubertal development in Swiss boys. Helv Paediatr Acta. 1983;38(3):211–228. 6618890

[8] Roche AF, Wellens R, Attie KM, Siervogel RM The timing of sexual maturation in a group of US white youths. J Pediatr Endocrinol Metab. 1995;8(1):11–18. 7584691 10.1515/jpem.1995.8.1.11

[9] Juul A, Teilmann G, Scheike T, Hertel NT, Holm K, Laursen EM, Main KM, Skakkebaek NE Pubertal development in Danish children: comparison of recent European and US data. Int J Androl. 2006;29(1):247–255. 16466546 10.1111/j.1365-2605.2005.00556.x

[10] Harlan WR, Grillo GP, Cornoni-Huntley J, Leaverton PE Secondary sex characteristics of boys 12 to 17 years of age: the U.S. Health Examination Survey. J Pediatr. 1979;95(2):293–297. 448573 10.1016/s0022-3476(79)80677-0

[11] Harlan WR, Harlan EA, Grillo GP Secondary sex characteristics of girls 12 to 17 years of age: the U.S. Health Examination Survey. J Pediatr. 1980;96(6):1074–1078. 7373473 10.1016/s0022-3476(80)80647-0

[12] Sun SS, Schubert CM, Chumlea WC, Roche AF, Kulin HE, Lee PA, Himes JH, Ryan AS National estimates of the timing of sexual maturation and racial differences among US children. Pediatrics. 2002;110(5):911–919. 12415029 10.1542/peds.110.5.911

[13] Aksglaede L, Sørensen K, Petersen JH, Skakkebaek NE, Juul A Recent decline in age at breast development: the Copenhagen Puberty Study. Pediatrics. 2009;123(5):e932–e939. 19403485 10.1542/peds.2008-2491

[14] Sørensen K, Aksglaede L, Petersen JH, Juul A Recent changes in pubertal timing in healthy Danish boys: associations with body mass index. J Clin Endocrinol Metab. 2010;95(1):263–270. 19926714 10.1210/jc.2009-1478

[15] Buck Louis GM, Gray LE Jr, Marcus M, Ojeda SR, Pescovitz OH, Witchel SF, Sippell W, Abbott DH, Soto A, Tyl RW, Bourguignon JP, Skakkebaek NE, Swan SH, Golub MS, Wabitsch M, Toppari J, Euling SY Environmental factors and puberty timing: expert panel research needs. Pediatrics. 2008;121(Suppl 3):S192–S207. 18245512 10.1542/peds.1813E

[16] Herman-Giddens ME, Slora EJ, Wasserman RC, Bourdony CJ, Bhapkar MV, Koch GG, Hasemeier CM Secondary sexual characteristics and menses in young girls seen in office practice: a study from the Pediatric Research in Office Settings network. Pediatrics. 1997;99(4):505–512. 9093289 10.1542/peds.99.4.505

[17] Herman-Giddens ME, Steffes J, Harris D, Slora E, Hussey M, Dowshen SA, Wasserman R, Serwint JR, Smitherman L, Reiter EO Secondary sexual characteristics in boys: data from the Pediatric Research in Office Settings Network. Pediatrics. 2012;130(5):e1058–e1068. 23085608 10.1542/peds.2011-3291

[18] Karpati AM, Rubin CH, Kieszak SM, Marcus M, Troiano RP Stature and pubertal stage assessment in American boys: the 1988–1994 Third National Health and Nutrition Examination Survey. J Adolesc Health. 2002;30(3):205–212. 11869928 10.1016/s1054-139x(01)00320-2

[19] Parent AS, Teilmann G, Juul A, Skakkebaek NE, Toppari J, Bourguignon JP The timing of normal puberty and the age limits of sexual precocity: variations around the world, secular trends, and changes after migration. Endocr Rev. 2003;24(5):668–693. 14570750 10.1210/er.2002-0019

[20] Mouritsen A, Aksglaede L, Soerensen K, Hagen CP, Petersen JH, Main KM, Juul A The pubertal transition in 179 healthy Danish children: associations between pubarche, adrenarche, gonadarche, and body composition. Eur J Endocrinol. 2012;168(2):129–136. 23093700 10.1530/EJE-12-0191

[21] Sørensen K, Mouritsen A, Aksglaede L, Hagen CP, Mogensen SS, Juul A Recent secular trends in pubertal timing: implications for evaluation and diagnosis of precocious puberty. Horm Res Paediatr. 2012;77(3):137–145. 22508036 10.1159/000336325

[22] Parent AS, Franssen D, Fudvoye J, Gérard A, Bourguignon JP Developmental variations in environmental influences including endocrine disruptors on pubertal timing and neuroendocrine control: revision of human observations and mechanistic insight from rodents. Front Neuroendocrinol. 2015;38:12–36. 25592640 10.1016/j.yfrne.2014.12.004

[23] Ellis BJ, Essex MJ Family environments, adrenarche, and sexual maturation: a longitudinal test of a life history model. Child Dev. 2007;78(6):1799–1817. 17988322 10.1111/j.1467-8624.2007.01092.x

[24] Parent AS, Rasier G, Gerard A, Heger S, Roth C, Mastronardi C, Jung H, Ojeda SR, Bourguignon JP Early onset of puberty: tracking genetic and environmental factors. Horm Res. 2005;64(Suppl 2):41–47. 16286770 10.1159/000087753

[25] Biro FM, Khoury P, Morrison JA Influence of obesity on timing of puberty. Int J Androl. 2006;29(1):272–277. 16371114 10.1111/j.1365-2605.2005.00602.x

[26] Biro FM, Lucky AW, Huster GA, Morrison JA Pubertal staging in boys. J Pediatr. 1995;127(1):100–102. 7608791 10.1016/s0022-3476(95)70265-2

[27] Lee PA, Guo SS, Kulin HE Age of puberty: data from the United States of America. APMIS. 2001;109(2):81–88. 11398998 10.1034/j.1600-0463.2001.d01-107.x

[28] Lee PA, Kulin HE, Guo SS Age of puberty among girls and the diagnosis of precocious puberty. Pediatrics. 2001;107(6):1493. 11403073 10.1542/peds.107.6.1493

[29] Mul D, Fredriks AM, van Buuren S, Oostdijk W, Verloove-Vanhorick SP, Wit JM Pubertal development in The Netherlands 1965–1997. Pediatr Res. 2001;50(4):479–486. 11568291 10.1203/00006450-200110000-00010

[30] Elks CE, Ong KK, Scott RA, van der Schouw YT, Brand JS, Wark PA, Amiano P, Balkau B, Barricarte A, Boeing H, Fonseca-Nunes A, Franks PW, Grioni S, Halkjaer J, Kaaks R, Key TJ, Khaw KT, Mattiello A, Nilsson PM, Overvad K, Palli D, Quirós JR, Rinaldi S, Rolandsson O, Romieu I, Sacerdote C, Sánchez MJ, Spijkerman AM, Tjonneland A, Tormo MJ, Tumino R, van der A DL, Forouhi NG, Sharp SJ, Langenberg C, Riboli E, Wareham NJ; InterAct Consortium. Age at menarche and type 2 diabetes risk: the EPIC-InterAct study. Diabetes Care. 2013;36(11):3526–3534. 24159179 10.2337/dc13-0446 PMC3816901

[31] Prentice P, Viner RM Pubertal timing and adult obesity and cardiometabolic risk in women and men: a systematic review and meta-analysis. Int J Obes. 2013;37(8):1036–1043. 10.1038/ijo.2012.177 23164700

[32] Bodicoat DH, Schoemaker MJ, Jones ME, McFadden E, Griffin J, Ashworth A, Swerdlow AJ Timing of pubertal stages and breast cancer risk: the Breakthrough Generations Study. Breast Cancer Res. 2014;16(1):R18. 24495528 10.1186/bcr3613 PMC3978643

[33] Charalampopoulos D, McLoughlin A, Elks CE, Ong KK Age at menarche and risks of all-cause and cardiovascular death: a systematic review and meta-analysis. Am J Epidemiol. 2014;180(1):29–40. 24920784 10.1093/aje/kwu113 PMC4070937

[34] Day FR, Elks CE, Murray A, Ong KK, Perry JR Puberty timing associated with diabetes, cardiovascular disease and also diverse health outcomes in men and women: the UK Biobank study. Sci Rep. 2015;5(1):11208. 26084728 10.1038/srep11208 PMC4471670

[35] Day FR, Bulik-Sullivan B, Hinds DA, Finucane HK, Murabito JM, Tung JY, Ong KK, Perry JR Shared genetic aetiology of puberty timing between sexes and with health-related outcomes. Nat Commun. 2015;6(1):8842. 26548314 10.1038/ncomms9842 PMC4667609

[36] Ahlgren M, Melbye M, Wohlfahrt J, Sørensen TI Growth patterns and the risk of breast cancer in women. N Engl J Med. 2004;351(16):1619–1626. 15483280 10.1056/NEJMoa040576

[37] Bhaskaran K, Douglas I, Forbes H, dos-Santos-Silva I, Leon DA, Smeeth L Body-mass index and risk of 22 specific cancers: a population-based cohort study of 5.24 million UK adults. Lancet. 2014;384(9945):755–765. 25129328 10.1016/S0140-6736(14)60892-8 PMC4151483

[38] Day FR, Thompson DJ, Helgason H, Chasman DI, Finucane H, Sulem P, Ruth KS, Whalen S, Sarkar AK, Albrecht E, Altmaier E, Amini M, Barbieri CM, Boutin T, Campbell A, Demerath E, Giri A, He C, Hottenga JJ, Karlsson R, Kolcic I, Loh PR, Lunetta KL, Mangino M, Marco B, McMahon G, Medland SE, Nolte IM, Noordam R, Nutile T, Paternoster L, Perjakova N, Porcu E, Rose LM, Schraut KE, Segrè AV, Smith AV, Stolk L, Teumer A, Andrulis IL, Bandinelli S, Beckmann MW, Benitez J, Bergmann S, Bochud M, Boerwinkle E, Bojesen SE, Bolla MK, Brand JS, Brauch H, Brenner H, Broer L, Brüning T, Buring JE, Campbell H, Catamo E, Chanock S, Chenevix-Trench G, Corre T, Couch FJ, Cousminer DL, Cox A, Crisponi L, Czene K, Davey Smith G, de Geus EJ, de Mutsert R, De Vivo I, Dennis J, Devilee P, Dos-Santos-Silva I, Dunning AM, Eriksson JG, Fasching PA, Fernández-Rhodes L, Ferrucci L, Flesch-Janys D, Franke L, Gabrielson M, Gandin I, Giles GG, Grallert H, Gudbjartsson DF, Guénel P, Hall P, Hallberg E, Hamann U, Harris TB, Hartman CA, Heiss G, Hooning MJ, Hopper JL, Hu F, Hunter DJ, Ikram MA, Im HK, Järvelin MR, Joshi PK, Karasik D, Kellis M, Kutalik Z, LaChance G, Lambrechts D, Langenberg C, Launer LJ, Laven JSE, Lenarduzzi S, Li J, Lind PA, Lindstrom S, Liu Y, Luan J, Mägi R, Mannermaa A, Mbarek H, McCarthy MI, Meisinger C, Meitinger T, Menni C, Metspalu A, Michailidou K, Milani L, Milne RL, Montgomery GW, Mulligan AM, Nalls MA, Navarro P, Nevanlinna H, Nyholt DR, Oldehinkel AJ, O’Mara TA, Padmanabhan S, Palotie A, Pedersen N, Peters A, Peto J, Pharoah PDP, Pouta A, Radice P, Rahman I, Ring SM, Robino A, Rosendaal FR, Rudan I, Rueedi R, Ruggiero D, Sala CF, Schmidt MK, Scott RA, Shah M, Sorice R, Southey MC, Sovio U, Stampfer M, Steri M, Strauch K, Tanaka T, Tikkanen E, Timpson NJ, Traglia M, Truong T, Tyrer JP, Uitterlinden AG, Edwards DR, Vitart V, Völker U, Vollenweider P, Wang Q, Widen E, van Dijk KW, Willemsen G, Winqvist R, Wolffenbuttel BHR, Zhao JH, Zoledziewska M, Zygmunt M, Alizadeh BZ, Boomsma DI, Ciullo M, Cucca F, Esko T, Franceschini N, Gieger C, Gudnason V, Hayward C, Kraft P, Lawlor DA, Magnusson PK, Martin NG, Mook-Kanamori DO, Nohr EA, Polasek O, Porteous D, Price AL, Ridker PM, Snieder H, Spector TD, Stöckl D, Toniolo D, Ulivi S, Visser JA, Völzke H, Wareham NJ, Wilson JF, Spurdle AB, Thorsteindottir U, Pollard KS, Easton DF, Tung JY, Chang-Claude J, Hinds D, Murray A, Murabito JM, Stefansson K, Ong KK, Perry JR; LifeLines Cohort Study; InterAct Consortium; kConFab/AOCS Investigators; Endometrial Cancer Association Consortium; Ovarian Cancer Association Consortium; PRACTICAL consortium. Genomic analyses identify hundreds of variants associated with age at menarche and support a role for puberty timing in cancer risk. Nat Genet. 2017;49(6):834–841. 28436984 10.1038/ng.3841 PMC5841952

[39] Parker SE, Troisi R, Wise LA, Palmer JR, Titus-Ernstoff L, Strohsnitter WC, Hatch EE Menarche, menopause, years of menstruation, and the incidence of osteoporosis: the influence of prenatal exposure to diethylstilbestrol. J Clin Endocrinol Metab. 2014;99(2):594–601. 24248183 10.1210/jc.2013-2954 PMC3913806

[40] Palmert MR, Dunkel L Delayed puberty. N Engl J Med. 2012;366(5):443–453. 22296078 10.1056/NEJMcp1109290

[41] Albanese A, Stanhope R Predictive factors in the determination of final height in boys with constitutional delay of growth and puberty. J Pediatr. 1995;126(4):545–550. 7699531 10.1016/s0022-3476(95)70347-0

[42] Gilsanz V, Chalfant J, Kalkwarf H, Zemel B, Lappe J, Oberfield S, Shepherd J, Wren T, Winer K Age at onset of puberty predicts bone mass in young adulthood. J Pediatr. 2011;158(1):100–105.e1–2. 20797727 10.1016/j.jpeds.2010.06.054 PMC4767165

[43] Sedlmeyer IL, Palmert MR Delayed puberty: analysis of a large case series from an academic center. J Clin Endocrinol Metab. 2002;87(4):1613–1620. 11932291 10.1210/jcem.87.4.8395

[44] Lawaetz JG, Hagen CP, Mieritz MG, Blomberg Jensen M, Petersen JH, Juul A Evaluation of 451 Danish boys with delayed puberty: diagnostic use of a new puberty nomogram and effects of oral testosterone therapy. J Clin Endocrinol Metab. 2015;100(4):1376–1385. 25594861 10.1210/jc.2014-3631

[45] Morris DH, Jones ME, Schoemaker MJ, Ashworth A, Swerdlow AJ Familial concordance for age at menarche: analyses from the Breakthrough Generations Study. Paediatr Perinat Epidemiol. 2011;25(3):306–311. 21470270 10.1111/j.1365-3016.2010.01183.x

[46] Boehm U, Bouloux PM, Dattani MT, de Roux N, Dodé C, Dunkel L, Dwyer AA, Giacobini P, Hardelin JP, Juul A, Maghnie M, Pitteloud N, Prevot V, Raivio T, Tena-Sempere M, Quinton R, Young J European consensus statement on congenital hypogonadotropic hypogonadism—pathogenesis, diagnosis and treatment. Nat Rev Endocrinol. 2015;11(9):547–564. 26194704 10.1038/nrendo.2015.112

[47] Franco B, Guioli S, Pragliola A, Incerti B, Bardoni B, Tonlorenzi R, Carrozzo R, Maestrini E, Pieretti M, Taillon-Miller P, Brown CJ, Willard HF, Lawrence C, Graziella Persico M, Camerino G, Ballabio A A gene deleted in Kallmann’s syndrome shares homology with neural cell adhesion and axonal path-finding molecules. Nature. 1991;353(6344):529–536. 1922361 10.1038/353529a0

[48] Cariboni A, Maggi R Kallmann’s syndrome, a neuronal migration defect. Cell Mol Life Sci. 2006;63(21):2512–2526. 16952059 10.1007/s00018-005-5604-3 PMC11136178

[49] Cadman SM, Kim SH, Hu Y, González-Martínez D, Bouloux PM Molecular pathogenesis of Kallmann’s syndrome. Horm Res. 2007;67(5):231–242. 17191030 10.1159/000098156

[50] Silveira LF, Latronico AC Approach to the patient with hypogonadotropic hypogonadism. J Clin Endocrinol Metab. 2013;98(5):1781–1788. 23650335 10.1210/jc.2012-3550

[51] Silveira LF, Trarbach EB, Latronico AC Genetics basis for GnRH-dependent pubertal disorders in humans. Mol Cell Endocrinol. 2010;324(1–2):30–38. 20188792 10.1016/j.mce.2010.02.023

[52] Hutchins BI, Kotan LD, Taylor-Burds C, Ozkan Y, Cheng PJ, Gurbuz F, Tiong JD, Mengen E, Yuksel B, Topaloglu AK, Wray S CCDC141 mutation identified in anosmic hypogonadotropic hypogonadism (Kallmann syndrome) alters GnRH neuronal migration. Endocrinology. 2016;157(5):1956–1966. 27014940 10.1210/en.2015-1846 PMC4870868

[53] Sarfati J, Bouvattier C, Bry-Gauillard H, Cartes A, Bouligand J, Young J Kallmann syndrome with *FGFR1* and *KAL1* mutations detected during fetal life. Orphanet J Rare Dis. 2015;10(1):71. 26051373 10.1186/s13023-015-0287-9 PMC4469106

[54] Sarfati J, Guiochon-Mantel A, Rondard P, Arnulf I, Garcia-Piñero A, Wolczynski S, Brailly-Tabard S, Bidet M, Ramos-Arroyo M, Mathieu M, Lienhardt-Roussie A, Morgan G, Turki Z, Bremont C, Lespinasse J, Du Boullay H, Chabbert-Buffet N, Jacquemont S, Reach G, De Talence N, Tonella P, Conrad B, Despert F, Delobel B, Brue T, Bouvattier C, Cabrol S, Pugeat M, Murat A, Bouchard P, Hardelin JP, Dodé C, Young J A comparative phenotypic study of Kallmann syndrome patients carrying monoallelic and biallelic mutations in the prokineticin 2 or prokineticin receptor 2 genes. J Clin Endocrinol Metab. 2010;95(2):659–669. 20022991 10.1210/jc.2009-0843

[55] Raivio T, Falardeau J, Dwyer A, Quinton R, Hayes FJ, Hughes VA, Cole LW, Pearce SH, Lee H, Boepple P, Crowley WF Jr, Pitteloud N Reversal of idiopathic hypogonadotropic hypogonadism. N Engl J Med. 2007;357(9):863–873. 17761590 10.1056/NEJMoa066494

[56] Pitteloud N, Meysing A, Quinton R, Acierno JS Jr, Dwyer AA, Plummer L, Fliers E, Boepple P, Hayes F, Seminara S, Hughes VA, Ma J, Bouloux P, Mohammadi M, Crowley WF Jr Mutations in fibroblast growth factor receptor 1 cause Kallmann syndrome with a wide spectrum of reproductive phenotypes. Mol Cell Endocrinol. 2006;254-255:60–69. 16764984 10.1016/j.mce.2006.04.021

[57] Dodé C, Levilliers J, Dupont JM, De Paepe A, Le Dû N, Soussi-Yanicostas N, Coimbra RS, Delmaghani S, Compain-Nouaille S, Baverel F, Pêcheux C, Le Tessier D, Cruaud C, Delpech M, Speleman F, Vermeulen S, Amalfitano A, Bachelot Y, Bouchard P, Cabrol S, Carel JC, Delemarre-van de Waal H, Goulet-Salmon B, Kottler ML, Richard O, Sanchez-Franco F, Saura R, Young J, Petit C, Hardelin JP Loss-of-function mutations in *FGFR1* cause autosomal dominant Kallmann syndrome. Nat Genet. 2003;33(4):463–465. 12627230 10.1038/ng1122

[58] Pitteloud N, Hayes FJ, Boepple PA, DeCruz S, Seminara SB, MacLaughlin DT, Crowley WF Jr The role of prior pubertal development, biochemical markers of testicular maturation, and genetics in elucidating the phenotypic heterogeneity of idiopathic hypogonadotropic hypogonadism. J Clin Endocrinol Metab. 2002;87(1):152–160. 11788640 10.1210/jcem.87.1.8131

[59] Hadziselimovic F On the descent of the epididymo-testicular unit, cryptorchidism, and prevention of infertility. Basic Clin Androl. 2017;27(1):21. 29163975 10.1186/s12610-017-0065-8 PMC5686796

[60] Kuiri-Hänninen T, Haanpää M, Turpeinen U, Hämäläinen E, Dunkel L, Sankilampi U Transient postnatal secretion of androgen hormones is associated with acne and sebaceous gland hypertrophy in early infancy. J Clin Endocrinol Metab. 2013;98(1):199–206. 23144469 10.1210/jc.2012-2680

[61] Kuiri-Hänninen T, Haanpää M, Turpeinen U, Hämäläinen E, Seuri R, Tyrväinen E, Sankilampi U, Dunkel L Postnatal ovarian activation has effects in estrogen target tissues in infant girls. J Clin Endocrinol Metab. 2013;98(12):4709–4716. 24217908 10.1210/jc.2013-1677

[62] Kuiri-Hänninen T, Kallio S, Seuri R, Tyrväinen E, Liakka A, Tapanainen J, Sankilampi U, Dunkel L Postnatal developmental changes in the pituitary-ovarian axis in preterm and term infant girls. J Clin Endocrinol Metab. 2011;96(11):3432–3439. 21900380 10.1210/jc.2011-1502

[63] Kuiri-Hänninen T, Seuri R, Tyrväinen E, Turpeinen U, Hämäläinen E, Stenman UH, Dunkel L, Sankilampi U Increased activity of the hypothalamic-pituitary-testicular axis in infancy results in increased androgen action in premature boys. J Clin Endocrinol Metab. 2011;96(1):98–105. 20881260 10.1210/jc.2010-1359

[64] Grinspon RP, Loreti N, Braslavsky D, Valeri C, Schteingart H, Ballerini MG, Bedecarrás P, Ambao V, Gottlieb S, Ropelato MG, Bergadá I, Campo SM, Rey RA Spreading the clinical window for diagnosing fetal-onset hypogonadism in boys. Front Endocrinol (Lausanne). 2014;5:51. 24847309 10.3389/fendo.2014.00051 PMC4019849

[65] Grumbach MM A window of opportunity: the diagnosis of gonadotropin deficiency in the male infant. J Clin Endocrinol Metab. 2005;90(5):3122–3127. 15728198 10.1210/jc.2004-2465

[66] Kuiri-Hänninen T, Sankilampi U, Dunkel L Activation of the hypothalamic-pituitary-gonadal axis in infancy: minipuberty. Horm Res Paediatr. 2014;82(2):73–80. 25012863 10.1159/000362414

[67] Dunkel L, Alfthan H, Stenman UH, Tapanainen P, Perheentupa J Pulsatile secretion of LH and FSH in prepubertal and early pubertal boys revealed by ultrasensitive time-resolved immunofluorometric assays. Pediatr Res. 1990;27(3):215–219. 2108425 10.1203/00006450-199003000-00003

[68] Albertsson-Wikland K, Rosberg S, Lannering B, Dunkel L, Selstam G, Norjavaara E Twenty-four-hour profiles of luteinizing hormone, follicle-stimulating hormone, testosterone, and estradiol levels: a semilongitudinal study throughout puberty in healthy boys. J Clin Endocrinol Metab. 1997;82(2):541–549. 9024251 10.1210/jcem.82.2.3778

[69] Dunkel L, Perheentupa J, Virtanen M, Mäenpää J Gonadotropin-releasing hormone test and human chorionic gonadotropin test in the diagnosis of gonadotropin deficiency in prepubertal boys. J Pediatr. 1985;107(3):388–392. 3928857 10.1016/s0022-3476(85)80512-6

[70] Dunkel L, Perheentupa J, Virtanen M, Mäenpää J GnRH and HCG tests are both necessary in differential diagnosis of male delayed puberty. Am J Dis Child. 1985;139(5):494–498. 3920897 10.1001/archpedi.1985.02140070068036

[71] Dunkel L, Perheentupa J, Sorva R Single versus repeated dose human chorionic gonadotropin stimulation in the differential diagnosis of hypogonadotropic hypogonadism. J Clin Endocrinol Metab. 1985;60(2):333–337. 3965492 10.1210/jcem-60-2-333

[72] Tata B, Huijbregts L, Jacquier S, Csaba Z, Genin E, Meyer V, Leka S, Dupont J, Charles P, Chevenne D, Carel JC, Léger J, de Roux N Haploinsufficiency of *Dmxl2*, encoding a synaptic protein, causes infertility associated with a loss of GnRH neurons in mouse. PLoS Biol. 2014;12(9):e1001952. 25248098 10.1371/journal.pbio.1001952 PMC4172557

[73] Jongmans MC, van Ravenswaaij-Arts CM, Pitteloud N, Ogata T, Sato N, Claahsen-van der Grinten HL, van der Donk K, Seminara S, Bergman JE, Brunner HG, Crowley WF Jr, Hoefsloot LH *CHD7* mutations in patients initially diagnosed with Kallmann syndrome—the clinical overlap with CHARGE syndrome. Clin Genet. 2009;75(1):65–71. 19021638 10.1111/j.1399-0004.2008.01107.x PMC2854009

[74] Legendre M, Gonzales M, Goudefroye G, Bilan F, Parisot P, Perez MJ, Bonnière M, Bessières B, Martinovic J, Delezoide AL, Jossic F, Fallet-Bianco C, Bucourt M, Tantau J, Loget P, Loeuillet L, Laurent N, Leroy B, Salhi H, Bigi N, Rouleau C, Guimiot F, Quélin C, Bazin A, Alby C, Ichkou A, Gesny R, Kitzis A, Ville Y, Lyonnet S, Razavi F, Gilbert-Dussardier B, Vekemans M, Attié-Bitach T Antenatal spectrum of CHARGE syndrome in 40 fetuses with *CHD7* mutations. J Med Genet. 2012;49(11):698–707. 23024289 10.1136/jmedgenet-2012-100926

[75] Balasubramanian R, Choi JH, Francescatto L, Willer J, Horton ER, Asimacopoulos EP, Stankovic KM, Plummer L, Buck CL, Quinton R, Nebesio TD, Mericq V, Merino PM, Meyer BF, Monies D, Gusella JF, Al Tassan N, Katsanis N, Crowley WF Jr Functionally compromised *CHD7* alleles in patients with isolated GnRH deficiency. Proc Natl Acad Sci USA. 2014;111(50):17953–17958. 25472840 10.1073/pnas.1417438111 PMC4273325

[76] Bergman JE, de Ronde W, Jongmans MC, Wolffenbuttel BH, Drop SL, Hermus A, Bocca G, Hoefsloot LH, van Ravenswaaij-Arts CM The results of *CHD7* analysis in clinically well-characterized patients with Kallmann syndrome. J Clin Endocrinol Metab. 2012;97(5):E858–E862. 22399515 10.1210/jc.2011-2652

[77] de Geus CM, Free RH, Verbist BM, Sival DA, Blake KD, Meiners LC, van Ravenswaaij-Arts CM Guidelines in CHARGE syndrome and the missing link: cranial imaging. Am J Med Genet C Semin Med Genet. 2017;175(4):450–464. 29168326 10.1002/ajmg.c.31593 PMC5765497

[78] Tan TS, Patel L, Gopal-Kothandapani JS, Ehtisham S, Ikazoboh EC, Hayward R, Aquilina K, Skae M, Thorp N, Pizer B, Didi M, Mallucci C, Blair JC, Gaze MN, Kamaly-Asl I, Spoudeas H, Clayton PE The neuroendocrine sequelae of paediatric craniopharyngioma: a 40-year meta-data analysis of 185 cases from three UK centres. Eur J Endocrinol. 2017;176(3):359–369. 28073908 10.1530/EJE-16-0812

[79] Karavitaki N, Brufani C, Warner JT, Adams CB, Richards P, Ansorge O, Shine B, Turner HE, Wass JA Craniopharyngiomas in children and adults: systematic analysis of 121 cases with long-term follow-up. Clin Endocrinol (Oxf). 2005;62(4):397–409. 15807869 10.1111/j.1365-2265.2005.02231.x

[80] Montefusco L, Harari S, Elia D, Rossi A, Specchia C, Torre O, Adda G, Arosio M Endocrine and metabolic assessment in adults with Langerhans cell histiocytosis. Eur J Intern Med. 2018;51:61–67. 29198444 10.1016/j.ejim.2017.11.011

[81] Ng Wing Tin S, Martin-Duverneuil N, Idbaih A, Garel C, Ribeiro M, Parker JL, Defachelles AS, Lambilliotte A, Barkaoui M, Munzer M, Gardembas M, Sibilia J, Lutz P, Fior R, Polak M, Robert A, Aumaitre O, Plantaz D, Armari-Alla C, Genereau T, Berard PM, Talom GN, Pennaforte JL, Le Pointe HD, Barthez MA, Couillault G, Haroche J, Mokhtari K, Donadieu J, Hoang-Xuan K; French LCH study group. Efficacy of vinblastine in central nervous system Langerhans cell histiocytosis: a nationwide retrospective study. Orphanet J Rare Dis. 2011;6(1):83. 22151964 10.1186/1750-1172-6-83 PMC3287163

[82] Shibamoto Y Management of central nervous system germinoma: proposal for a modern strategy. Prog Neurol Surg. 2009;23:119–129. 19329866 10.1159/000210058

[83] Haas RJ, Janka G, Gaedicke G, Kohne E, Netzel B Therapy of acute lymphocytic leukemia in childhood with intermediate dose methotrexate and CNS irradiation. A report of the ALL 77-02 study group. Blut. 1983;47(6):321–331. 6580929 10.1007/BF00320346

[84] Wallace WH Oncofertility and preservation of reproductive capacity in children and young adults. Cancer. 2011;117(10 Suppl):2301–2310. 21523750 10.1002/cncr.26045

[85] Sklar CA, Antal Z, Chemaitilly W, Cohen LE, Follin C, Meacham LR, Murad MH Hypothalamic–pituitary and growth disorders in survivors of childhood cancer: an Endocrine Society clinical practice guideline. J Clin Endocrinol Metab. 2018;103(8):2761–2784. 29982476 10.1210/jc.2018-01175

[86] Fang Q, George AS, Brinkmeier ML, Mortensen AH, Gergics P, Cheung LY, Daly AZ, Ajmal A, Pérez Millán MI, Ozel AB, Kitzman JO, Mills RE, Li JZ, Camper SA Genetics of combined pituitary hormone deficiency: roadmap into the genome era. Endocr Rev. 2016;37(6):636–675. 27828722 10.1210/er.2016-1101 PMC5155665

[87] Parks JS, Brown MR, Hurley DL, Phelps CJ, Wajnrajch MP Heritable disorders of pituitary development. J Clin Endocrinol Metab. 1999;84(12):4362–4370. 10599689 10.1210/jcem.84.12.6209

[88] Achermann JC, Gu WX, Kotlar TJ, Meeks JJ, Sabacan LP, Seminara SB, Habiby RL, Hindmarsh PC, Bick DP, Sherins RJ, Crowley WF Jr, Layman LC, Jameson JL Mutational analysis of *DAX1* in patients with hypogonadotropic hypogonadism or pubertal delay. J Clin Endocrinol Metab. 1999;84(12):4497–4500. 10599708 10.1210/jcem.84.12.6269

[89] Farooqi IS, Wangensteen T, Collins S, Kimber W, Matarese G, Keogh JM, Lank E, Bottomley B, Lopez-Fernandez J, Ferraz-Amaro I, Dattani MT, Ercan O, Myhre AG, Retterstol L, Stanhope R, Edge JA, McKenzie S, Lessan N, Ghodsi M, De Rosa V, Perna F, Fontana S, Barroso I, Undlien DE, O’Rahilly S Clinical and molecular genetic spectrum of congenital deficiency of the leptin receptor. N Engl J Med. 2007;356(3):237–247. 17229951 10.1056/NEJMoa063988 PMC2670197

[90] Savage MO, Beattie RM, Camacho-Hübner C, Walker-Smith JA, Sanderson IR Growth in Crohn’s disease. Acta Paediatr Suppl. 1999;88(428):89–92. 10.1111/j.1651-2227.1999.tb14360.x 10102061

[91] Saari A, Harju S, Mäkitie O, Saha MT, Dunkel L, Sankilampi U Systematic growth monitoring for the early detection of celiac disease in children. JAMA Pediatr. 2015;169(3):e1525. 25730696 10.1001/jamapediatrics.2015.25

[92] Claris-Appiani A, Bianchi ML, Bini P, Ballabio G, Caraceni MP, Funari C, Terzi F, Romeo L, Rusconi R Growth in young children with chronic renal failure. Pediatr Nephrol. 1989;3(3):301–304. 2702111 10.1007/BF00858536

[93] Johannesson M, Gottlieb C, Hjelte L Delayed puberty in girls with cystic fibrosis despite good clinical status. Pediatrics. 1997;99(1):29–34. 8989333 10.1542/peds.99.1.29

[94] Winters SJ, ed. Male Hypogonadism: Basic, Clinical, and Therapeutic Principles. Totowa, NJ: Humana Press; 2004.

[95] Haffner D, Zivicnjak M Pubertal development in children with chronic kidney disease. Pediatr Nephrol. 2017;32(6):949–964. 27464647 10.1007/s00467-016-3432-3

[96] Pozo J, Argente J Delayed puberty in chronic illness. Best Pract Res Clin Endocrinol Metab. 2002;16(1):73–90. 11987900 10.1053/beem.2002.0182

[97] Lee CC, Chen CM, Lee ST, Wei KC, Pai PC, Toh CH, Chuang CC Prediction of long-term post-operative testosterone replacement requirement based on the pre-operative tumor volume and testosterone level in pituitary macroadenoma. Sci Rep. 2015;5(1):16194. 26537232 10.1038/srep16194 PMC5155724

[98] Miller KK Endocrine effects of anorexia nervosa. Endocrinol Metab Clin North Am. 2013;42(3):515–528. 24011884 10.1016/j.ecl.2013.05.007 PMC3769686

[99] Frisch RE, McArthur JW Menstrual cycles: fatness as a determinant of minimum weight for height necessary for their maintenance or onset. Science. 1974;185(4155):949–951. 4469672 10.1126/science.185.4155.949

[100] Munoz MT, Argente J Anorexia nervosa in female adolescents: endocrine and bone mineral density disturbances. Eur J Endocrinol. 2002;147(3):275–286. 12213663 10.1530/eje.0.1470275

[101] Mantzoros C, Flier JS, Lesem MD, Brewerton TD, Jimerson DC Cerebrospinal fluid leptin in anorexia nervosa: correlation with nutritional status and potential role in resistance to weight gain. J Clin Endocrinol Metab. 1997;82(6):1845–1851. 9177394 10.1210/jcem.82.6.4006

[102] Hebebrand J, Muller TD, Holtkamp K, Herpertz-Dahlmann B The role of leptin in anorexia nervosa: clinical implications. Mol Psychiatry. 2007;12(1):23–35. 17060920 10.1038/sj.mp.4001909

[103] Welt CK, Chan JL, Bullen J, Murphy R, Smith P, DePaoli AM, Karalis A, Mantzoros CS Recombinant human leptin in women with hypothalamic amenorrhea. N Engl J Med. 2004;351(10):987–997. 15342807 10.1056/NEJMoa040388

[104] Watson HJ, Bulik CM Update on the treatment of anorexia nervosa: review of clinical trials, practice guidelines and emerging interventions. Psychol Med. 2013;43(12):2477–2500. 23217606 10.1017/S0033291712002620

[105] Roberts AC, McClure RD, Weiner RI, Brooks GA Overtraining affects male reproductive status. Fertil Steril. 1993;60(4):686–692. 8405526 10.1016/s0015-0282(16)56223-2

[106] Caronia LM, Martin C, Welt CK, Sykiotis GP, Quinton R, Thambundit A, Avbelj M, Dhruvakumar S, Plummer L, Hughes VA, Seminara SB, Boepple PA, Sidis Y, Crowley WF Jr, Martin KA, Hall JE, Pitteloud N A genetic basis for functional hypothalamic amenorrhea. N Engl J Med. 2011;364(3):215–225. 21247312 10.1056/NEJMoa0911064 PMC3045842

[107] Fechner PY, Davenport ML, Qualy RL, Ross JL, Gunther DF, Eugster EA, Huseman C, Zagar AJ, Quigley CA; Toddler Turner Study Group. Differences in follicle-stimulating hormone secretion between 45,X monosomy Turner syndrome and 45,X/46,XX mosaicism are evident at an early age. J Clin Endocrinol Metab. 2006;91(12):4896–4902. 16968797 10.1210/jc.2006-1157

[108] Bondy CA; Turner Syndrome Study Group. Care of girls and women with Turner syndrome: a guideline of the Turner Syndrome Study Group. J Clin Endocrinol Metab. 2007;92(1):10–25. 17047017 10.1210/jc.2006-1374

[109] Saenger P, Wikland KA, Conway GS, Davenport M, Gravholt CH, Hintz R, Hovatta O, Hultcrantz M, Landin-Wilhelmsen K, Lin A, Lippe B, Pasquino AM, Ranke MB, Rosenfeld R, Silberbach M; Fifth International Symposium on Turner Syndrome. Recommendations for the diagnosis and management of Turner syndrome. J Clin Endocrinol Metab. 2001;86(7):3061–3069. 11443168 10.1210/jcem.86.7.7683

[110] Improda N, Rezzuto M, Alfano S, Parenti G, Vajro P, Pignata C, Salerno M Precocious puberty in Turner syndrome: report of a case and review of the literature. Ital J Pediatr. 2012;38(1):54. 23075274 10.1186/1824-7288-38-54 PMC3481396

[111] Cox L, Liu JH Primary ovarian insufficiency: an update. Int J Womens Health. 2014;6:235–243. 24591848 10.2147/IJWH.S37636 PMC3934663

[112] Aittomäki K, Lucena JL, Pakarinen P, Sistonen P, Tapanainen J, Gromoll J, Kaskikari R, Sankila EM, Lehväslaiho H, Engel AR, Nieschlag E, Huhtaniemi I, de la Chapelle A Mutation in the follicle-stimulating hormone receptor gene causes hereditary hypergonadotropic ovarian failure. Cell. 1995;82(6):959–968. 7553856 10.1016/0092-8674(95)90275-9

[113] Juul A, Aksglaede L, Bay K, Grigor KM, Skakkebaek NE Klinefelter syndrome: the forgotten syndrome: basic and clinical questions posed to an international group of scientists. Acta Paediatr. 2011;100(6):791–792. 10.1111/j.1651-2227.2011.02283.x 21521364

[114] Rives N, Milazzo JP, Perdrix A, Castanet M, Joly-Hélas G, Sibert L, Bironneau A, Way A, Macé B The feasibility of fertility preservation in adolescents with Klinefelter syndrome. Hum Reprod. 2013;28(6):1468–1479. 23539613 10.1093/humrep/det084

[115] Ahmed SF, Achermann JC, Arlt W, Balen A, Conway G, Edwards Z, Elford S, Hughes IA, Izatt L, Krone N, Miles H, O’Toole S, Perry L, Sanders C, Simmonds M, Watt A, Willis D Society for Endocrinology UK guidance on the initial evaluation of an infant or an adolescent with a suspected disorder of sex development (revised 2015). Clin Endocrinol (Oxf). 2016;84(5):771–788. 26270788 10.1111/cen.12857 PMC4855619

[116] Crinò A, Schiaffini R, Ciampalini P, Spera S, Beccaria L, Benzi F, Bosio L, Corrias A, Gargantini L, Salvatoni A, Tonini G, Trifirò G, Livieri C; Genetic Obesity Study Group of Italian Society of Pediatric Endocrinology and Diabetology (SIEDP). Hypogonadism and pubertal development in Prader-Willi syndrome. Eur J Pediatr. 2003;162(5):327–333. 12692714 10.1007/s00431-002-1132-4

[117] Lauper JM, Krause A, Vaughan TL, Monnat RJ Jr Spectrum and risk of neoplasia in Werner syndrome: a systematic review. PLoS One. 2013;8(4):e59709. 23573208 10.1371/journal.pone.0059709 PMC3613408

[118] Lauper JM, Monnat RJ Jr Diabetes mellitus and cancer in Werner syndrome. Acta Diabetol. 2014;51(1):159–161. 23386316 10.1007/s00592-013-0456-z PMC3669637

[119] Astuti D, Sabir A, Fulton P, Zatyka M, Williams D, Hardy C, Milan G, Favaretto F, Yu-Wai-Man P, Rohayem J, López de Heredia M, Hershey T, Tranebjaerg L, Chen JH, Chaussenot A, Nunes V, Marshall B, McAfferty S, Tillmann V, Maffei P, Paquis-Flucklinger V, Geberhiwot T, Mlynarski W, Parkinson K, Picard V, Bueno GE, Dias R, Arnold A, Richens C, Paisey R, Urano F, Semple R, Sinnott R, Barrett TG Monogenic diabetes syndromes: locus-specific databases for Alström, Wolfram, and thiamine-responsive megaloblastic anemia. Hum Mutat. 2017;38(7):764–777. 28432734 10.1002/humu.23233 PMC5535005

[120] Mendez HM, Opitz JM Noonan syndrome: a review. Am J Med Genet. 1985;21(3):493–506. 3895929 10.1002/ajmg.1320210312

[121] Narayan P Genetic Models for the study of luteinizing hormone receptor function. Front Endocrinol (Lausanne). 2015;6:152. 26483755 10.3389/fendo.2015.00152 PMC4586495

[122] Bramble MS, Goldstein EH, Lipson A, Ngun T, Eskin A, Gosschalk JE, Roach L, Vashist N, Barseghyan H, Lee E, Arboleda VA, Vaiman D, Yuksel Z, Fellous M, Vilain E A novel follicle-stimulating hormone receptor mutation causing primary ovarian failure: a fertility application of whole exome sequencing. Hum Reprod. 2016;31(4):905–914. 26911863 10.1093/humrep/dew025 PMC5007606

[123] Jankowska K Premature ovarian failure. Przegl Menopauz. 2017;16(2):51–56. 10.5114/pm.2017.68592 PMC5509972 28721130

[124] Tapanainen JS, Aittomäki K, Min J, Vaskivuo T, Huhtaniemi IT Men homozygous for an inactivating mutation of the follicle-stimulating hormone (FSH) receptor gene present variable suppression of spermatogenesis and fertility. Nat Genet. 1997;15(2):205–206. 9020851 10.1038/ng0297-205

[125] Abitbol L, Zborovski S, Palmert MR Evaluation of delayed puberty: what diagnostic tests should be performed in the seemingly otherwise well adolescent? Arch Dis Child. 2016;101(8):767–771. 27190100 10.1136/archdischild-2015-310375

[126] Doty RL Measurement of chemosensory function. World J Otorhinolaryngol Head Neck Surg. 2018;4(1):11–28. 30035257 10.1016/j.wjorl.2018.03.001 PMC6051764

[127] Wehkalampi K, Vangonen K, Laine T, Dunkel L Progressive reduction of relative height in childhood predicts adult stature below target height in boys with constitutional delay of growth and puberty. Horm Res. 2007;68(2):99–104. 17377395 10.1159/000101011

[128] Crowne EC, Shalet SM, Wallace WH, Eminson DM, Price DA Final height in boys with untreated constitutional delay in growth and puberty. Arch Dis Child. 1990;65(10):1109–1112. 2248500 10.1136/adc.65.10.1109 PMC1792322

[129] Crowne EC, Shalet SM, Wallace WH, Eminson DM, Price DA Final height in girls with untreated constitutional delay in growth and puberty. Eur J Pediatr. 1991;150(10):708–712. 1915481 10.1007/BF01958760

[130] LaFranchi S, Hanna CE, Mandel SH Constitutional delay of growth: expected versus final adult height. Pediatrics. 1991;87(1):82–87. 1984625

[131] Albanese A, Stanhope R Does constitutional delayed puberty cause segmental disproportion and short stature? Eur J Pediatr. 1993;152(4):293–296. 8482274 10.1007/BF01956736

[132] Arrigo T, Cisternino M, Luca De F, Saggese G, Messina MF, Pasquino AM, De Sanctis V Final height outcome in both untreated and testosterone-treated boys with constitutional delay of growth and puberty. J Pediatr Endocrinol Metab. 1996;9(5):511–517. 8961126 10.1515/jpem.1996.9.5.511

[133] Brämswig JH, Fasse M, Holthoff ML, von Lengerke HJ, von Petrykowski W, Schellong G Adult height in boys and girls with untreated short stature and constitutional delay of growth and puberty: accuracy of five different methods of height prediction. J Pediatr. 1990;117(6):886–891. 2246686 10.1016/s0022-3476(05)80127-1

[134] Cools BL, Rooman R, Op De Beeck L, Du Caju MV Boys with a simple delayed puberty reach their target height. Horm Res. 2008;70(4):209–214. 18772593 10.1159/000137663

[135] Rensonnet C, Kanen F, Coremans C, Ernould C, Albert A, Bourguignon JP Pubertal growth as a determinant of adult height in boys with constitutional delay of growth and puberty. Horm Res. 1999;51(5):223–229. 10559666 10.1159/000023375

[136] Sperlich M, Butenandt O, Schwarz HP Final height and predicted height in boys with untreated constitutional growth delay. Eur J Pediatr. 1995;154(8):627–632. 7588962 10.1007/BF02079065

[137] Volta C, Ghizzoni L, Buono T, Ferrari F, Virdis R, Bernasconi S Final height in a group of untreated children with constitutional growth delay. Helv Paediatr Acta. 1988;43(3):171–176. 3220788

[138] Varimo T, Miettinen PJ, Känsäkoski J, Raivio T, Hero M Congenital hypogonadotropic hypogonadism, functional hypogonadotropism or constitutional delay of growth and puberty? An analysis of a large patient series from a single tertiary center. Hum Reprod. 2017;32(1):147–153. 27927844 10.1093/humrep/dew294

[139] Wu FC, Brown DC, Butler GE, Stirling HF, Kelnar CJ Early morning plasma testosterone is an accurate predictor of imminent pubertal development in prepubertal boys. J Clin Endocrinol Metab. 1993;76(1):26–31. 8421096 10.1210/jcem.76.1.8421096

[140] Harrington J, Palmert MR Clinical review: distinguishing constitutional delay of growth and puberty from isolated hypogonadotropic hypogonadism: critical appraisal of available diagnostic tests. J Clin Endocrinol Metab. 2012;97(9):3056–3067. 22723321 10.1210/jc.2012-1598

[141] Demir A, Voutilainen R, Juul A, Dunkel L, Alfthan H, Skakkebaek NE, Stenman UH Increase in first morning voided urinary luteinizing hormone levels precedes the physical onset of puberty. J Clin Endocrinol Metab. 1996;81(8):2963–2967. 8768859 10.1210/jcem.81.8.8768859

[142] Coutant R, Biette-Demeneix E, Bouvattier C, Bouhours-Nouet N, Gatelais F, Dufresne S, Rouleau S, Lahlou N Baseline inhibin B and anti-Mullerian hormone measurements for diagnosis of hypogonadotropic hypogonadism (HH) in boys with delayed puberty. J Clin Endocrinol Metab. 2010;95(12):5225–5232. 20826577 10.1210/jc.2010-1535

[143] Adan L, Lechevalier P, Couto-Silva AC, Boissan M, Trivin C, Brailly-Tabard S, Brauner R Plasma inhibin B and antimüllerian hormone concentrations in boys: discriminating between congenital hypogonadotropic hypogonadism and constitutional pubertal delay. Med Sci Monit. 2010;16(11):CR511–CR517. 20980953

[144] Binder G, Schweizer R, Haber P, Blumenstock G, Braun R. Accuracy of endocrine tests for detecting hypogonadotropic hypogonadism in girls. J Pediatr. 2015;167(3):674–678.e1. 26095287 10.1016/j.jpeds.2015.05.039

[145] Bondy CA, Matura LA, Wooten N, Troendle J, Zinn AR, Bakalov VK The physical phenotype of girls and women with Turner syndrome is not X-imprinted. Hum Genet. 2007;121(3-4):469–474. 17242899 10.1007/s00439-007-0324-4

[146] Aksglaede L, Petersen JH, Main KM, Skakkebaek NE, Juul A High normal testosterone levels in infants with non-mosaic Klinefelter’s syndrome. Eur J Endocrinol. 2007 Sep;157(3):345–350. 17766718 10.1530/EJE-07-0310

[147] Wikström AM, Bay K, Hero M, Andersson AM, Dunkel L Serum insulin-like factor 3 levels during puberty in healthy boys and boys with Klinefelter syndrome. J Clin Endocrinol Metab. 2006;91(11):4705–4708. 16926256 10.1210/jc.2006-0669

[148] Wikström AM, Dunkel L Testicular function in Klinefelter syndrome. Horm Res. 2008;69(6):317–326. 18504390 10.1159/000117387

[149] Wikström AM, Dunkel L Klinefelter syndrome. Best Pract Res Clin Endocrinol Metab. 2011;25(2):239–250. 21397196 10.1016/j.beem.2010.09.006

[150] Wikström AM, Dunkel L, Wickman S, Norjavaara E, Ankarberg-Lindgren C, Raivio T Are adolescent boys with Klinefelter syndrome androgen deficient? A longitudinal study of Finnish 47,XXY boys. Pediatr Res. 2006;59(6):854–859. 16641204 10.1203/01.pdr.0000219386.31398.c3

[151] Bergadá I, Milani C, Bedecarrás P, Andreone L, Ropelato MG, Gottlieb S, Bergadá C, Campo S, Rey RA Time course of the serum gonadotropin surge, inhibins, and anti-Müllerian hormone in normal newborn males during the first month of life. J Clin Endocrinol Metab. 2006;91(10):4092–4098. 16849404 10.1210/jc.2006-1079

[152] Andersson AM, Müller J, Skakkebaek NE Different roles of prepubertal and postpubertal germ cells and Sertoli cells in the regulation of serum inhibin B levels. J Clin Endocrinol Metab. 1998;83(12):4451–4458. 9851793 10.1210/jcem.83.12.5360

[153] Bergadá I, Rojas G, Ropelato G, Ayuso S, Bergadá C, Campo S Sexual dimorphism in circulating monomeric and dimeric inhibins in normal boys and girls from birth to puberty. Clin Endocrinol (Oxf). 1999;51(4):455–460. 10583312 10.1046/j.1365-2265.1999.00814.x

[154] Ong KK, Elks CE, Li S, Zhao JH, Luan J, Andersen LB, Bingham SA, Brage S, Smith GD, Ekelund U, Gillson CJ, Glaser B, Golding J, Hardy R, Khaw KT, Kuh D, Luben R, Marcus M, McGeehin MA, Ness AR, Northstone K, Ring SM, Rubin C, Sims MA, Song K, Strachan DP, Vollenweider P, Waeber G, Waterworth DM, Wong A, Deloukas P, Barroso I, Mooser V, Loos RJ, Wareham NJ Genetic variation in *LIN28B* is associated with the timing of puberty. Nat Genet. 2009;41(6):729–733. 19448623 10.1038/ng.382 PMC3000552

[155] Tommiska J, Wehkalampi K, Vaaralahti K, Laitinen EM, Raivio T, Dunkel L *LIN28B* in constitutional delay of growth and puberty. J Clin Endocrinol Metab. 2010;95(6):3063–3066. 20350940 10.1210/jc.2009-2344

[156] Silveira-Neto AP, Leal LF, Emerman AB, Henderson KD, Piskounova E, Henderson BE, Gregory RI, Silveira LF, Hirschhorn JN, Nguyen TT, Beneduzzi D, Tusset C, Reis AC, Brito VN, Mendonca BB, Palmert MR, Antonini SR, Latronico AC Absence of functional *LIN28B* mutations in a large cohort of patients with idiopathic central precocious puberty. Horm Res Paediatr. 2012;78(3):144–150. 22964795 10.1159/000342212 PMC3526815

[157] Elks CE, Perry JR, Sulem P, Chasman DI, Franceschini N, He C, Lunetta KL, Visser JA, Byrne EM, Cousminer DL, Gudbjartsson DF, Esko T, Feenstra B, Hottenga JJ, Koller DL, Kutalik Z, Lin P, Mangino M, Marongiu M, McArdle PF, Smith AV, Stolk L, van Wingerden SH, Zhao JH, Albrecht E, Corre T, Ingelsson E, Hayward C, Magnusson PK, Smith EN, Ulivi S, Warrington NM, Zgaga L, Alavere H, Amin N, Aspelund T, Bandinelli S, Barroso I, Berenson GS, Bergmann S, Blackburn H, Boerwinkle E, Buring JE, Busonero F, Campbell H, Chanock SJ, Chen W, Cornelis MC, Couper D, Coviello AD, d’Adamo P, de Faire U, de Geus EJ, Deloukas P, Döring A, Smith GD, Easton DF, Eiriksdottir G, Emilsson V, Eriksson J, Ferrucci L, Folsom AR, Foroud T, Garcia M, Gasparini P, Geller F, Gieger C, Gudnason V, Hall P, Hankinson SE, Ferreli L, Heath AC, Hernandez DG, Hofman A, Hu FB, Illig T, Järvelin MR, Johnson AD, Karasik D, Khaw KT, Kiel DP, Kilpeläinen TO, Kolcic I, Kraft P, Launer LJ, Laven JS, Li S, Liu J, Levy D, Martin NG, McArdle WL, Melbye M, Mooser V, Murray JC, Murray SS, Nalls MA, Navarro P, Nelis M, Ness AR, Northstone K, Oostra BA, Peacock M, Palmer LJ, Palotie A, Paré G, Parker AN, Pedersen NL, Peltonen L, Pennell CE, Pharoah P, Polasek O, Plump AS, Pouta A, Porcu E, Rafnar T, Rice JP, Ring SM, Rivadeneira F, Rudan I, Sala C, Salomaa V, Sanna S, Schlessinger D, Schork NJ, Scuteri A, Segrè AV, Shuldiner AR, Soranzo N, Sovio U, Srinivasan SR, Strachan DP, Tammesoo ML, Tikkanen E, Toniolo D, Tsui K, Tryggvadottir L, Tyrer J, Uda M, van Dam RM, van Meurs JB, Vollenweider P, Waeber G, Wareham NJ, Waterworth DM, Weedon MN, Wichmann HE, Willemsen G, Wilson JF, Wright AF, Young L, Zhai G, Zhuang WV, Bierut LJ, Boomsma DI, Boyd HA, Crisponi L, Demerath EW, van Duijn CM, Econs MJ, Harris TB, Hunter DJ, Loos RJ, Metspalu A, Montgomery GW, Ridker PM, Spector TD, Streeten EA, Stefansson K, Thorsteinsdottir U, Uitterlinden AG, Widen E, Murabito JM, Ong KK, Murray A; GIANT Consortium. Thirty new loci for age at menarche identified by a meta-analysis of genome-wide association studies. Nat Genet. 2010;42(12):1077–1085. 21102462 10.1038/ng.714 PMC3140055

[158] Perry JR, Day F, Elks CE, Sulem P, Thompson DJ, Ferreira T, He C, Chasman DI, Esko T, Thorleifsson G, Albrecht E, Ang WQ, Corre T, Cousminer DL, Feenstra B, Franceschini N, Ganna A, Johnson AD, Kjellqvist S, Lunetta KL, McMahon G, Nolte IM, Paternoster L, Porcu E, Smith AV, Stolk L, Teumer A, Tšernikova N, Tikkanen E, Ulivi S, Wagner EK, Amin N, Bierut LJ, Byrne EM, Hottenga JJ, Koller DL, Mangino M, Pers TH, Yerges-Armstrong LM, Zhao JH, Andrulis IL, Anton-Culver H, Atsma F, Bandinelli S, Beckmann MW, Benitez J, Blomqvist C, Bojesen SE, Bolla MK, Bonanni B, Brauch H, Brenner H, Buring JE, Chang-Claude J, Chanock S, Chen J, Chenevix-Trench G, Collée JM, Couch FJ, Couper D, Coveillo AD, Cox A, Czene K, D’adamo AP, Smith GD, De Vivo I, Demerath EW, Dennis J, Devilee P, Dieffenbach AK, Dunning AM, Eiriksdottir G, Eriksson JG, Fasching PA, Ferrucci L, Flesch-Janys D, Flyger H, Foroud T, Franke L, Garcia ME, García-Closas M, Geller F, de Geus EE, Giles GG, Gudbjartsson DF, Gudnason V, Guénel P, Guo S, Hall P, Hamann U, Haring R, Hartman CA, Heath AC, Hofman A, Hooning MJ, Hopper JL, Hu FB, Hunter DJ, Karasik D, Kiel DP, Knight JA, Kosma VM, Kutalik Z, Lai S, Lambrechts D, Lindblom A, Mägi R, Magnusson PK, Mannermaa A, Martin NG, Masson G, McArdle PF, McArdle WL, Melbye M, Michailidou K, Mihailov E, Milani L, Milne RL, Nevanlinna H, Neven P, Nohr EA, Oldehinkel AJ, Oostra BA, Palotie A, Peacock M, Pedersen NL, Peterlongo P, Peto J, Pharoah PD, Postma DS, Pouta A, Pylkäs K, Radice P, Ring S, Rivadeneira F, Robino A, Rose LM, Rudolph A, Salomaa V, Sanna S, Schlessinger D, Schmidt MK, Southey MC, Sovio U, Stampfer MJ, Stöckl D, Storniolo AM, Timpson NJ, Tyrer J, Visser JA, Vollenweider P, Völzke H, Waeber G, Waldenberger M, Wallaschofski H, Wang Q, Willemsen G, Winqvist R, Wolffenbuttel BH, Wright MJ, Boomsma DI, Econs MJ, Khaw KT, Loos RJ, McCarthy MI, Montgomery GW, Rice JP, Streeten EA, Thorsteinsdottir U, van Duijn CM, Alizadeh BZ, Bergmann S, Boerwinkle E, Boyd HA, Crisponi L, Gasparini P, Gieger C, Harris TB, Ingelsson E, Järvelin MR, Kraft P, Lawlor D, Metspalu A, Pennell CE, Ridker PM, Snieder H, Sørensen TI, Spector TD, Strachan DP, Uitterlinden AG, Wareham NJ, Widen E, Zygmunt M, Murray A, Easton DF, Stefansson K, Murabito JM, Ong KK; Australian Ovarian Cancer Study; GENICA Network; kConFabLifeLines Cohort Study; InterAct Consortium; Early Growth Genetics (EGG) Consortium. Parent-of-origin-specific allelic associations among 106 genomic loci for age at menarche. Nature. 2014;514(7520):92–97. 25231870 10.1038/nature13545 PMC4185210

[159] Morrison KE, Rodgers AB, Morgan CP, Bale TL Epigenetic mechanisms in pubertal brain maturation. Neuroscience. 2014;264:17–24. 24239720 10.1016/j.neuroscience.2013.11.014 PMC3959229

[160] Yang C, Ye J, Li X, Gao X, Zhang K, Luo L, Ding J, Zhang Y, Li Y, Cao H, Ling Y, Zhang X, Liu Y, Fang F DNA methylation patterns in the hypothalamus of female pubertal goats. PLoS One. 2016;11(10):e0165327. 27788248 10.1371/journal.pone.0165327 PMC5082945

[161] Peters J The role of genomic imprinting in biology and disease: an expanding view. Nat Rev Genet. 2014;15(8):517–530. 24958438 10.1038/nrg3766

[162] Abreu AP, Dauber A, Macedo DB, Noel SD, Brito VN, Gill JC, Cukier P, Thompson IR, Navarro VM, Gagliardi PC, Rodrigues T, Kochi C, Longui CA, Beckers D, de Zegher F, Montenegro LR, Mendonca BB, Carroll RS, Hirschhorn JN, Latronico AC, Kaiser UB Central precocious puberty caused by mutations in the imprinted gene *MKRN3*. N Engl J Med. 2013;368(26):2467–2475. 23738509 10.1056/NEJMoa1302160 PMC3808195

[163] Dauber A, Cunha-Silva M, Macedo DB, Brito VN, Abreu AP, Roberts SA, Montenegro LR, Andrew M, Kirby A, Weirauch MT, Labilloy G, Bessa DS, Carroll RS, Jacobs DC, Chappell PE, Mendonca BB, Haig D, Kaiser UB, Latronico AC Paternally inherited *DLK1* deletion associated with familial central precocious puberty. J Clin Endocrinol Metab. 2017;102(5):1557–1567. 28324015 10.1210/jc.2016-3677 PMC5443333

[164] Hirsch HJ, Eldar-Geva T, Bennaroch F, Pollak Y, Gross-Tsur V Sexual dichotomy of gonadal function in Prader–Willi syndrome from early infancy through the fourth decade. Hum Reprod. 2015;30(11):2587–2596. 26345685 10.1093/humrep/dev213

[165] Butler MG Genomic imprinting disorders in humans: a mini-review. J Assist Reprod Genet. 2009;26(9-10):477–486. 19844787 10.1007/s10815-009-9353-3 PMC2788689

[166] Lee HS, Hwang JS Central precocious puberty in a girl with Prader-Willi syndrome. J Pediatr Endocrinol Metab. 2013;26(11–12):1201–1204. 23740678 10.1515/jpem-2013-0040

[167] Kanber D, Giltay J, Wieczorek D, Zogel C, Hochstenbach R, Caliebe A, Kuechler A, Horsthemke B, Buiting K A paternal deletion of *MKRN3*, *MAGEL2* and *NDN* does not result in Prader–Willi syndrome. Eur J Hum Genet. 2009;17(5):582–590. 19066619 10.1038/ejhg.2008.232 PMC2986273

[168] de Smith AJ, Purmann C, Walters RG, Ellis RJ, Holder SE, Van Haelst MM, Brady AF, Fairbrother UL, Dattani M, Keogh JM, Henning E, Yeo GS, O’Rahilly S, Froguel P, Farooqi IS, Blakemore AI A deletion of the HBII-85 class of small nucleolar RNAs (snoRNAs) is associated with hyperphagia, obesity and hypogonadism. Hum Mol Genet. 2009;18(17):3257–3265. 19498035 10.1093/hmg/ddp263 PMC2722987

[169] Messina A, Langlet F, Chachlaki K, Roa J, Rasika S, Jouy N, Gallet S, Gaytan F, Parkash J, Tena-Sempere M, Giacobini P, Prevot V A microRNA switch regulates the rise in hypothalamic GnRH production before puberty (published correction appears in *Nat Neurosci*. 2016;19(8):1115). Nat Neurosci. 2016;19(6):835–844. 27135215 10.1038/nn.4298

[170] Ahmed K, LaPierre MP, Gasser E, Denzler R, Yang Y, Rülicke T, Kero J, Latreille M, Stoffel M Loss of microRNA-7a2 induces hypogonadotropic hypogonadism and infertility. J Clin Invest. 2017;127(3):1061–1074. 28218624 10.1172/JCI90031 PMC5330717

[171] Mouritsen A, Aksglaede L, Sørensen K, Mogensen SS, Leffers H, Main KM, Frederiksen H, Andersson AM, Skakkebaek NE, Juul A Hypothesis: exposure to endocrine-disrupting chemicals may interfere with timing of puberty. Int J Androl. 2010;33(2):346–359. 20487042 10.1111/j.1365-2605.2010.01051.x

[172] Swan SH, Main KM, Liu F, Stewart SL, Kruse RL, Calafat AM, Mao CS, Redmon JB, Ternand CL, Sullivan S, Teague JL; Study for Future Families Research Team. Decrease in anogenital distance among male infants with prenatal phthalate exposure. Environ Health Perspect. 2005;113(8):1056–1061. 16079079 10.1289/ehp.8100 PMC1280349

[173] van den Driesche S, Macdonald J, Anderson RA, Johnston ZC, Chetty T, Smith LB, Mckinnell C, Dean A, Homer NZ, Jorgensen A, Camacho-Moll ME, Sharpe RM, Mitchell RT Prolonged exposure to acetaminophen reduces testosterone production by the human fetal testis in a xenograft model. Sci Transl Med. 2015;7(288):288ra80. 10.1126/scitranslmed.aaa4097 PMC5044981 25995226

[174] Li X, Sun Z, Manthari RK, Li M, Guo Q, Wang J Effect of gestational exposure to arsenic on puberty in offspring female mice. Chemosphere. 2018;202:119–126. 29567609 10.1016/j.chemosphere.2018.03.095

[175] Knorr D, Bidlingmaier F, Butenandt O, Fendel H Plasma testosterone in male puberty. I. Physiology of plasma testosterone. Acta Endocrinol (Copenh). 1974;75(1):181–194. 4363842 10.1530/acta.0.0750181

[176] Brambilla DJ, Matsumoto AM, Araujo AB, McKinlay JB The effect of diurnal variation on clinical measurement of serum testosterone and other sex hormone levels in men. J Clin Endocrinol Metab. 2009;94(3):907–913. 19088162 10.1210/jc.2008-1902 PMC2681273

[177] Wennink JM, Delemarre-van de Waal HA, Schoemaker R, Schoemaker H, Schoemaker J Luteinizing hormone and follicle stimulating hormone secretion patterns in girls throughout puberty measured using highly sensitive immunoradiometric assays. Clin Endocrinol (Oxf). 1990;33(3):333–344. 2123756 10.1111/j.1365-2265.1990.tb00498.x

[178] Semaan SJ, Dhamija S, Kim J, Ku EC, Kauffman AS Assessment of epigenetic contributions to sexually-dimorphic *Kiss1* expression in the anteroventral periventricular nucleus of mice. Endocrinology. 2012;153(4):1875–1886. 22374971 10.1210/en.2011-1975 PMC3320252

[179] Macedo DB, Cukier P, Mendonca BB, Latronico AC, Brito VN Advances in the etiology, diagnosis and treatment of central precocious puberty [in Portuguese]. Arq Bras Endocrinol Metabol. 2014;58(2):108–117. 24830587 10.1590/0004-2730000002931

[180] Corre C, Shinoda G, Zhu H, Cousminer DL, Crossman C, Bellissimo C, Goldenberg A, Daley GQ, Palmert MR Sex-specific regulation of weight and puberty by the *Lin28/let-7* axis. J Endocrinol. 2016;228(3):179–191. 26698568 10.1530/JOE-15-0360 PMC4772724

[181] He Q, Karlberg J BMI in childhood and its association with height gain, timing of puberty, and final height. Pediatr Res. 2001;49(2):244–251. 11158521 10.1203/00006450-200102000-00019

[182] Lee JM, Wasserman R, Kaciroti N, Gebremariam A, Steffes J, Dowshen S, Harris D, Serwint J, Abney D, Smitherman L, Reiter E, Herman-Giddens ME Timing of puberty in overweight versus obese boys. Pediatrics. 2016;137(2):e20150164. 26817933 10.1542/peds.2015-0164

[183] Ahmed ML, Ong KK, Morrell DJ, Cox L, Drayer N, Perry L, Preece MA, Dunger DB Longitudinal study of leptin concentrations during puberty: sex differences and relationship to changes in body composition. J Clin Endocrinol Metab. 1999;84(3):899–905. 10084568 10.1210/jcem.84.3.5559

[184] Farooqi IS Leptin and the onset of puberty: insights from rodent and human genetics. Semin Reprod Med. 2002;20(2):139–144. 12087499 10.1055/s-2002-32505

[185] Elias CF, Purohit D Leptin signaling and circuits in puberty and fertility. Cell Mol Life Sci. 2013;70(5):841–862. 22851226 10.1007/s00018-012-1095-1 PMC3568469

[186] Bellefontaine N, Chachlaki K, Parkash J, Vanacker C, Colledge W, d’Anglemont de Tassigny X, Garthwaite J, Bouret SG, Prevot V Leptin-dependent neuronal NO signaling in the preoptic hypothalamus facilitates reproduction. J Clin Invest. 2014;124(6):2550–2559. 24812663 10.1172/JCI65928 PMC4089460

[187] Plant TM Neuroendocrine control of the onset of puberty. Front Neuroendocrinol. 2015;38:73–88. 25913220 10.1016/j.yfrne.2015.04.002 PMC4457677

[188] Couce ME, Cottam D, Esplen J, Teijeiro R, Schauer P, Burguera B Potential role of hypothalamic ghrelin in the pathogenesis of human obesity. J Endocrinol Invest. 2006;29(7):599–605. 16957407 10.1007/BF03344158

[189] Pomerants T, Tillmann V, Jürimäe J, Jürimäe T Relationship between ghrelin and anthropometrical, body composition parameters and testosterone levels in boys at different stages of puberty. J Endocrinol Invest. 2006;29(11):962–967. 17259792 10.1007/BF03349208

[190] Pomerants T, Tillmann V, Karelson K, Jürimäe J, Jürimäe T Ghrelin response to acute aerobic exercise in boys at different stages of puberty. Horm Metab Res. 2006;38(11):752–757. 17111303 10.1055/s-2006-955087

[191] Lebrethon MC, Aganina A, Fournier M, Gérard A, Parent AS, Bourguignon JP Effects of in vivo and in vitro administration of ghrelin, leptin and neuropeptide mediators on pulsatile gonadotrophin-releasing hormone secretion from male rat hypothalamus before and after puberty. J Neuroendocrinol. 2007;19(3):181–188. 17280591 10.1111/j.1365-2826.2006.01518.x

[192] Aksglaede L, Juul A, Olsen LW, Sørensen TI Age at puberty and the emerging obesity epidemic. PLoS One. 2009;4(12):e8450. 20041184 10.1371/journal.pone.0008450 PMC2793517

[193] Albuisson J, Pêcheux C, Carel JC, Lacombe D, Leheup B, Lapuzina P, Bouchard P, Legius E, Matthijs G, Wasniewska M, Delpech M, Young J, Hardelin JP, Dodé C Kallmann syndrome: 14 novel mutations in *KAL1* and *FGFR1* (*KAL2*). Hum Mutat. 2005;25(1):98–99. 10.1002/humu.9298 15605412

[194] Beate K, Joseph N, Nicolas R, Wolfram K Genetics of isolated hypogonadotropic hypogonadism: role of GnRH receptor and other genes. Int J Endocrinol. 2012;2012:147893. 22229029 10.1155/2012/147893 PMC3249753

[195] Bhagavath B, Layman LC The genetics of hypogonadotropic hypogonadism. Semin Reprod Med. 2007;25(4):272–286. 17594608 10.1055/s-2007-980221

[196] Cerrato F, Shagoury J, Kralickova M, Dwyer A, Falardeau J, Ozata M, Van Vliet G, Bouloux P, Hall JE, Hayes FJ, Pitteloud N, Martin KA, Welt C, Seminara SB. Coding sequence analysis of *GNRHR* and *GPR54* in patients with congenital and adult-onset forms of hypogonadotropic hypogonadism. Eur J Endocrinol. 2006;155(Suppl 1):S3–S10. 17074994 10.1530/eje.1.02235

[197] Dodé C, Teixeira L, Levilliers J, Fouveaut C, Bouchard P, Kottler ML, Lespinasse J, Lienhardt-Roussie A, Mathieu M, Moerman A, Morgan G, Murat A, Toublanc JE, Wolczynski S, Delpech M, Petit C, Young J, Hardelin JP Kallmann syndrome: mutations in the genes encoding prokineticin-2 and prokineticin receptor-2. PLoS Genet. 2006;2(10):e175. 17054399 10.1371/journal.pgen.0020175 PMC1617130

[198] Guran T, Tolhurst G, Bereket A, Rocha N, Porter K, Turan S, Gribble FM, Kotan LD, Akcay T, Atay Z, Canan H, Serin A, O’Rahilly S, Reimann F, Semple RK, Topaloglu AK Hypogonadotropic hypogonadism due to a novel missense mutation in the first extracellular loop of the neurokinin B receptor. J Clin Endocrinol Metab. 2009;94(10):3633–3639. 19755480 10.1210/jc.2009-0551 PMC4306717

[199] Karges B, de Roux N Molecular genetics of isolated hypogonadotropic hypogonadism and Kallmann syndrome. Endocr Dev. 2005;8:67–80. 15722618 10.1159/000084094

[200] Pedersen-White JR, Chorich LP, Bick DP, Sherins RJ, Layman LC The prevalence of intragenic deletions in patients with idiopathic hypogonadotropic hypogonadism and Kallmann syndrome. Mol Hum Reprod. 2008;14(6):367–370. 18463157 10.1093/molehr/gan027 PMC2434956

[201] Pitteloud N, Quinton R, Pearce S, Raivio T, Acierno J, Dwyer A, Plummer L, Hughes V, Seminara S, Cheng YZ, Li WP, Maccoll G, Eliseenkova AV, Olsen SK, Ibrahimi OA, Hayes FJ, Boepple P, Hall JE, Bouloux P, Mohammadi M, Crowley W Digenic mutations account for variable phenotypes in idiopathic hypogonadotropic hypogonadism. J Clin Invest. 2007;117(2):457–463. 17235395 10.1172/JCI29884 PMC1765517

[202] Pitteloud N, Zhang C, Pignatelli D, Li JD, Raivio T, Cole LW, Plummer L, Jacobson-Dickman EE, Mellon PL, Zhou QY, Crowley WF Jr Loss-of-function mutation in the prokineticin 2 gene causes Kallmann syndrome and normosmic idiopathic hypogonadotropic hypogonadism. Proc Natl Acad Sci USA. 2007;104(44):17447–17452. 17959774 10.1073/pnas.0707173104 PMC2077276

[203] Topaloglu AK, Tello JA, Kotan LD, Ozbek MN, Yilmaz MB, Erdogan S, Gurbuz F, Temiz F, Millar RP, Yuksel B Inactivating *KISS1* mutation and hypogonadotropic hypogonadism. N Engl J Med. 2012;366(7):629–635. 22335740 10.1056/NEJMoa1111184

[204] Tornberg J, Sykiotis GP, Keefe K, Plummer L, Hoang X, Hall JE, Quinton R, Seminara SB, Hughes V, Van Vliet G, Van Uum S, Crowley WF, Habuchi H, Kimata K, Pitteloud N, Bülow HE *Heparan sulfate 6-O-sulfotransferase 1*, a gene involved in extracellular sugar modifications, is mutated in patients with idiopathic hypogonadotrophic hypogonadism. Proc Natl Acad Sci USA. 2011;108(28):11524–11529. 21700882 10.1073/pnas.1102284108 PMC3136273

[205] Villanueva C, de Roux N *FGFR1* mutations in Kallmann syndrome. Front Horm Res. 2010;39:51–61. 20389085 10.1159/000312693

[206] Macedo DB, Abreu AP, Reis AC, Montenegro LR, Dauber A, Beneduzzi D, Cukier P, Silveira LF, Teles MG, Carroll RS, Junior GG, Filho GG, Gucev Z, Arnhold IJ, de Castro M, Moreira AC, Martinelli CE Jr, Hirschhorn JN, Mendonca BB, Brito VN, Antonini SR, Kaiser UB, Latronico AC Central precocious puberty that appears to be sporadic caused by paternally inherited mutations in the imprinted gene makorin ring finger 3. J Clin Endocrinol Metab. 2014;99(6):E1097–E1103. 24628548 10.1210/jc.2013-3126 PMC4037732

[207] Silveira LG, Noel SD, Silveira-Neto AP, Abreu AP, Brito VN, Santos MG, Bianco SD, Kuohung W, Xu S, Gryngarten M, Escobar ME, Arnhold IJ, Mendonca BB, Kaiser UB, Latronico AC Mutations of the *KISS1* gene in disorders of puberty. J Clin Endocrinol Metab. 2010;95(5):2276–2280. 20237166 10.1210/jc.2009-2421 PMC2869552

[208] Teles MG, Bianco SD, Brito VN, Trarbach EB, Kuohung W, Xu S, Seminara SB, Mendonca BB, Kaiser UB, Latronico AC A *GPR54*-activating mutation in a patient with central precocious puberty. N Engl J Med. 2008;358(7):709–715. 18272894 10.1056/NEJMoa073443 PMC2859966

[209] Villanueva C, Jacobson-Dickman E, Xu C, Manouvrier S, Dwyer AA, Sykiotis GP, Beenken A, Liu Y, Tommiska J, Hu Y, Tiosano D, Gerard M, Leger J, Drouin-Garraud V, Lefebvre H, Polak M, Carel JC, Phan-Hug F, Hauschild M, Plummer L, Rey JP, Raivio T, Bouloux P, Sidis Y, Mohammadi M, de Roux N, Pitteloud N Congenital hypogonadotropic hypogonadism with split hand/foot malformation: a clinical entity with a high frequency of FGFR1 mutations. Genet Med. 2015;17(8):651–659. 25394172 10.1038/gim.2014.166 PMC4430466

[210] Emerick JE, Vogt KS Endocrine manifestations and management of Prader-Willi syndrome. Int J Pediatr Endocrinol. 2013;2013(1):14. 23962041 10.1186/1687-9856-2013-14 PMC3751775

[211] Forsythe E, Beales PL Bardet–Biedl syndrome. Eur J Hum Genet. 2013;21(1):8–13. 22713813 10.1038/ejhg.2012.115 PMC3522196

[212] Verloes A, Temple IK, Bonnet S, Bottani A Coloboma, mental retardation, hypogonadism, and obesity: critical review of the so-called Biemond syndrome type 2, updated nosology, and delineation of three “new” syndromes. Am J Med Genet. 1997;69(4):370–379. 9098485 10.1002/(sici)1096-8628(19970414)69:4<370::aid-ajmg7>3.0.co;2-p

[213] Dauber A, Hirschhorn JN, Picker J, Maher TA, Milunsky A Delayed puberty due to a novel mutation in *CHD7* causing CHARGE syndrome. Pediatrics. 2010;126(6):e1594–e1598. 21041284 10.1542/peds.2010-0164

[214] Loureiro M, Reis F, Robalo B, Pereira C, Sampaio L Adrenal hypoplasia congenita: a rare cause of primary adrenal insufficiency and hypogonadotropic hypogonadism. Pediatr Rep. 2015;7(3):5936. 26500747 10.4081/pr.2015.5936 PMC4594446

[215] Nagasaki K, Kubota T, Kobayashi H, Sawada H, Numakura C, Harada S, Takasawa K, Minamitani K, Ishii T, Okada S, Kamasaki H, Sugihara S, Adachi M, Tajima T Clinical characteristics of septo-optic dysplasia accompanied by congenital central hypothyroidism in Japan. Clin Pediatr Endocrinol. 2017;26(4):207–213. 29026269 10.1297/cpe.26.207 PMC5627221

[216] Szakszon K, Felszeghy E, Csízy I, Józsa T, Káposzta R, Balogh E, Oláh E, Balogh I, Berényi E, Knegt AC, Ilyés I Endocrine and anatomical findings in a case of solitary median maxillary central incisor syndrome. Eur J Med Genet. 2012;55(2):109–111. 22138217 10.1016/j.ejmg.2011.11.002

[217] Turner G, Lower KM, White SM, Delatycki M, Lampe AK, Wright M, Smith JC, Kerr B, Schelley S, Hoyme HE, De Vries BB, Kleefstra T, Grompe M, Cox B, Gecz J, Partington M The clinical picture of the Börjeson–Forssman–Lehmann syndrome in males and heterozygous females with *PHF6* mutations. Clin Genet. 2004;65(3):226–232. 14756673 10.1111/j.0009-9163.2004.00215.x

[218] Wortmann SB, Espeel M, Almeida L, Reimer A, Bosboom D, Roels F, de Brouwer AP, Wevers RA Inborn errors of metabolism in the biosynthesis and remodelling of phospholipids. J Inherit Metab Dis. 2015;38(1):99–110. 25178427 10.1007/s10545-014-9759-7

[219] Sidhoum VF, Chan YM, Lippincott MF, Balasubramanian R, Quinton R, Plummer L, Dwyer A, Pitteloud N, Hayes FJ, Hall JE, Martin KA, Boepple PA, Seminara SB Reversal and relapse of hypogonadotropic hypogonadism: resilience and fragility of the reproductive neuroendocrine system. J Clin Endocrinol Metab. 2014;99(3):861–870. 24423288 10.1210/jc.2013-2809 PMC3942233

[220] Cassatella D, Howard SR, Acierno JS, Acierno JS, Xu C, Papadakis GE, Santoni FA, Dwyer AA, Santini S, Sykiotis GP, Chambion C, Meylan J, Marino L, Favre L, Li J, Liu X, Zhang J, Bouloux PM, Geyter C, Paepe A, Dhillo WS, Ferrara JM, Hauschild M, Lang-Muritano M, Lemke JR, Flück C, Nemeth A, Phan-Hug F, Pignatelli D, Popovic V, Pekic S, Quinton R, Szinnai G, l′Allemand D, Konrad D, Sharif S, Iyidir ÖT, Stevenson BJ, Yang H, Dunkel L, Pitteloud N Congenital hypogonadotropic hypogonadism and constitutional delay of growth and puberty have distinct genetic architectures. Eur J Endocrinol. 2018 Apr;178(4):377–388. 29419413 10.1530/EJE-17-0568 PMC5863472

[221] Akutsu S, Takada M, Ohki-Hamazaki H, Murakami S, Arai Y Origin of luteinizing hormone-releasing hormone (LHRH) neurons in the chick embryo: effect of the olfactory placode ablation. Neurosci Lett. 1992;142(2):241–244. 1454222 10.1016/0304-3940(92)90382-h

[222] Cariboni A, Maggi R, Parnavelas JG From nose to fertility: the long migratory journey of gonadotropin-releasing hormone neurons. Trends Neurosci. 2007;30(12):638–644. 17981344 10.1016/j.tins.2007.09.002

[223] Wray S From nose to brain: development of gonadotrophin-releasing hormone-1 neurones. J Neuroendocrinol. 2010;22(7):743–753. 20646175 10.1111/j.1365-2826.2010.02034.x PMC2919238

[224] Kramer PR, Wray S Novel gene expressed in nasal region influences outgrowth of olfactory axons and migration of luteinizing hormone-releasing hormone (LHRH) neurons. Genes Dev. 2000;14(14):1824–1834. 10898796 PMC316793

[225] Gill JC, Tsai PS Expression of a dominant negative FGF receptor in developing GNRH1 neurons disrupts axon outgrowth and targeting to the median eminence. Biol Reprod. 2006;74(3):463–472. 16280414 10.1095/biolreprod.105.046904

[226] Tobet SA, Schwarting GA Minireview: recent progress in gonadotropin-releasing hormone neuronal migration. Endocrinology. 2006;147(3):1159–1165. 16373413 10.1210/en.2005-1275

[227] Cariboni A, Hickok J, Rakic S, Andrews W, Maggi R, Tischkau S, Parnavelas JG Neuropilins and their ligands are important in the migration of gonadotropin-releasing hormone neurons. J Neurosci. 2007;27(9):2387–2395. 17329436 10.1523/JNEUROSCI.5075-06.2007 PMC6673474

[228] Cariboni A, Rakic S, Liapi A, Maggi R, Goffinet A, Parnavelas JG Reelin provides an inhibitory signal in the migration of gonadotropin-releasing hormone neurons. Development. 2005;132(21):4709–4718. 16207762 10.1242/dev.02033

[229] Giacobini P, Giampietro C, Fioretto M, Maggi R, Cariboni A, Perroteau I, Fasolo A Hepatocyte growth factor/scatter factor facilitates migration of GN-11 immortalized LHRH neurons. Endocrinology. 2002;143(9):3306–3315. 12193542 10.1210/en.2002-220146

[230] Magni P, Dozio E, Ruscica M, Watanobe H, Cariboni A, Zaninetti R, Motta M, Maggi R Leukemia inhibitory factor induces the chemomigration of immortalized gonadotropin-releasing hormone neurons through the independent activation of the Janus kinase/signal transducer and activator of transcription 3, mitogen-activated protein kinase/extracellularly regulated kinase 1/2, and phosphatidylinositol 3-kinase/Akt signaling pathways. Mol Endocrinol. 2007;21(5):1163–1174. 17299136 10.1210/me.2006-0270

[231] Schwanzel-Fukuda M, Abraham S, Crossin KL, Edelman GM, Pfaff DW Immunocytochemical demonstration of neural cell adhesion molecule (NCAM) along the migration route of luteinizing hormone-releasing hormone (LHRH) neurons in mice. J Comp Neurol. 1992;321(1):1–18. 1613133 10.1002/cne.903210102

[232] Schwarting GA, Henion TR, Nugent JD, Caplan B, Tobet S Stromal cell-derived factor-1 (chemokine C-X-C motif ligand 12) and chemokine C-X-C motif receptor 4 are required for migration of gonadotropin-releasing hormone neurons to the forebrain. J Neurosci. 2006;26(25):6834–6840. 16793890 10.1523/JNEUROSCI.1728-06.2006 PMC6673820

[233] Herbison AE, Porteous R, Pape JR, Mora JM, Hurst PR Gonadotropin-releasing hormone neuron requirements for puberty, ovulation, and fertility. Endocrinology. 2008;149(2):597–604. 18006629 10.1210/en.2007-1139 PMC6101186

[234] Schwanzel-Fukuda M, Bick D, Pfaff DW Luteinizing hormone-releasing hormone (LHRH)-expressing cells do not migrate normally in an inherited hypogonadal (Kallmann) syndrome. Brain Res Mol Brain Res. 1989;6(4):311–326. 2687610 10.1016/0169-328x(89)90076-4

[235] Cariboni A, Pimpinelli F, Colamarino S, Zaninetti R, Piccolella M, Rumio C, Piva F, Rugarli EI, Maggi R The product of X-linked Kallmann’s syndrome gene (*KAL1*) affects the migratory activity of gonadotropin-releasing hormone (GnRH)-producing neurons. Hum Mol Genet. 2004;13(22):2781–2791. 15471890 10.1093/hmg/ddh309

[236] Yanicostas C, Herbomel E, Dipietromaria A, Soussi-Yanicostas N Anosmin-1a is required for fasciculation and terminal targeting of olfactory sensory neuron axons in the zebrafish olfactory system. Mol Cell Endocrinol. 2009;312(1–2):53–60. 19464344 10.1016/j.mce.2009.04.017

[237] Ojeda SR, Roth C, Mungenast A, Heger S, Mastronardi C, Parent AS, Lomniczi A, Jung H Neuroendocrine mechanisms controlling female puberty: new approaches, new concepts. Int J Androl. 2006;29(1):256–263, discussion 286–290. 16466547 10.1111/j.1365-2605.2005.00619.x

[238] Schauer C, Tong T, Petitjean H, Blum T, Peron S, Mai O, Schmitz F, Boehm U, Leinders-Zufall T Hypothalamic gonadotropin-releasing hormone (GnRH) receptor neurons fire in synchrony with the female reproductive cycle. J Neurophysiol. 2015;114(2):1008–1021. 26063780 10.1152/jn.00357.2015 PMC4725124

[239] Purnelle G, Gérard A, Czajkowski V, Bourguignon JP Pulsatile secretion of gonadotropin-releasing hormone by rat hypothalamic explants without cell bodies of GnRH neurons (published correction appears in *Neuroendocrinology.* 1998;**67**(1):57). Neuroendocrinology. 1997;66(5):305–312. 9387849 10.1159/000127253

[240] Colledge WH, Mei H, d’Anglemont de Tassigny X Mouse models to study the central regulation of puberty. Mol Cell Endocrinol. 2010;324(1-2):12–20. 20083157 10.1016/j.mce.2010.01.015

[241] Ojeda SR, Lomniczi A, Mastronardi C, Heger S, Roth C, Parent AS, Matagne V, Mungenast AE Minireview: the neuroendocrine regulation of puberty: is the time ripe for a systems biology approach? Endocrinology. 2006;147(3):1166–1174. 16373420 10.1210/en.2005-1136

[242] de Roux N, Genin E, Carel JC, Matsuda F, Chaussain JL, Milgrom E Hypogonadotropic hypogonadism due to loss of function of the KiSS1-derived peptide receptor GPR54. Proc Natl Acad Sci USA. 2003;100(19):10972–10976. 12944565 10.1073/pnas.1834399100 PMC196911

[243] Seminara SB, Messager S, Chatzidaki EE, Thresher RR, Acierno JS Jr, Shagoury JK, Bo-Abbas Y, Kuohung W, Schwinof KM, Hendrick AG, Zahn D, Dixon J, Kaiser UB, Slaugenhaupt SA, Gusella JF, O’Rahilly S, Carlton MB, Crowley WF Jr, Aparicio SA, Colledge WH The *GPR54* gene as a regulator of puberty. N Engl J Med. 2003;349(17):1614–1627. 14573733 10.1056/NEJMoa035322

[244] Poling MC, Kauffman AS Organizational and activational effects of sex steroids on kisspeptin neuron development. Front Neuroendocrinol. 2013;34(1):3–17. 22728025 10.1016/j.yfrne.2012.06.001 PMC3725275

[245] Tena-Sempere M Roles of kisspeptins in the control of hypothalamic-gonadotropic function: focus on sexual differentiation and puberty onset. Endocr Dev. 2010;17:52–62. 19955756 10.1159/000262528

[246] Lehman MN, Hileman SM, Goodman RL Neuroanatomy of the kisspeptin signaling system in mammals: comparative and developmental aspects. Adv Exp Med Biol. 2013;784:27–62. 23550001 10.1007/978-1-4614-6199-9_3 PMC4059209

[247] Navarro VM, Fernández-Fernández R, Castellano JM, Roa J, Mayen A, Barreiro ML, Gaytan F, Aguilar E, Pinilla L, Dieguez C, Tena-Sempere M Advanced vaginal opening and precocious activation of the reproductive axis by KiSS-1 peptide, the endogenous ligand of GPR54. J Physiol. 2004;561(Pt 2):379–386. 15486019 10.1113/jphysiol.2004.072298 PMC1665361

[248] Tena-Sempere M Kisspeptin signaling in the brain: recent developments and future challenges. Mol Cell Endocrinol. 2010;314(2):164–169. 19464345 10.1016/j.mce.2009.05.004

[249] Pinilla L, Aguilar E, Dieguez C, Millar RP, Tena-Sempere M Kisspeptins and reproduction: physiological roles and regulatory mechanisms. Physiol Rev. 2012;92(3):1235–1316. 22811428 10.1152/physrev.00037.2010

[250] Sandoval-Guzmán T, Rance NE Central injection of senktide, an NK_3_ receptor agonist, or neuropeptide Y inhibits LH secretion and induces different patterns of Fos expression in the rat hypothalamus. Brain Res. 2004;1026(2):307–312. 15488494 10.1016/j.brainres.2004.08.026

[251] Topaloglu AK, Reimann F, Guclu M, Yalin AS, Kotan LD, Porter KM, Serin A, Mungan NO, Cook JR, Imamoglu S, Akalin NS, Yuksel B, O’Rahilly S, Semple RK TAC3 and TACR3 mutations in familial hypogonadotropic hypogonadism reveal a key role for Neurokinin B in the central control of reproduction. Nat Genet. 2009;41(3):354–358. 19079066 10.1038/ng.306 PMC4312696

[252] Rance NE Menopause and the human hypothalamus: evidence for the role of kisspeptin/neurokinin B neurons in the regulation of estrogen negative feedback. Peptides. 2009;30(1):111–122. 18614256 10.1016/j.peptides.2008.05.016 PMC2632595

[253] Ramaswamy S, Seminara SB, Plant TM Evidence from the agonadal juvenile male rhesus monkey (*Macaca mulatta*) for the view that the action of neurokinin B to trigger gonadotropin-releasing hormone release is upstream from the kisspeptin receptor. Neuroendocrinology. 2011;94(3):237–245. 21832818 10.1159/000329045 PMC3238032

[254] Ducret E, Anderson GM, Herbison AE RFamide-related peptide-3, a mammalian gonadotropin-inhibitory hormone ortholog, regulates gonadotropin-releasing hormone neuron firing in the mouse. Endocrinology. 2009;150(6):2799–2804. 19131572 10.1210/en.2008-1623

[255] Poling MC, Quennell JH, Anderson GM, Kauffman AS Kisspeptin neurones do not directly signal to RFRP-3 neurones but RFRP-3 may directly modulate a subset of hypothalamic kisspeptin cells in mice. J Neuroendocrinol. 2013;25(10):876–886. 23927071 10.1111/jne.12084 PMC4022484

[256] Bourguignon JP, Gérard A, Purnelle G, Czajkowski V, Yamanaka C, Lemaître M, Rigo JM, Moonen G, Franchimont P Duality of glutamatergic and GABAergic control of pulsatile GnRH secretion by rat hypothalamic explants: II. Reduced NR2C- and GABAA-receptor-mediated inhibition at initiation of sexual maturation. J Neuroendocrinol. 1997;9(3):193–199. 9089470 10.1046/j.1365-2826.1997.00568.x

[257] Erecińska M, Silver IA Metabolism and role of glutamate in mammalian brain. Prog Neurobiol. 1990;35(4):245–296. 1980745 10.1016/0301-0082(90)90013-7

[258] Mitsushima D, Hei DL, Terasawa E gamma-Aminobutyric acid is an inhibitory neurotransmitter restricting the release of luteinizing hormone-releasing hormone before the onset of puberty. Proc Natl Acad Sci USA. 1994;91(1):395–399. 8278400 10.1073/pnas.91.1.395 PMC42954

[259] Ojeda SR, Lomniczi A, Sandau U Contribution of glial–neuronal interactions to the neuroendocrine control of female puberty. Eur J Neurosci. 2010;32(12):2003–2010. 21143655 10.1111/j.1460-9568.2010.07515.x PMC3058235

[260] Ojeda SR, Lomniczi A, Sandau US Glial-gonadotrophin hormone (GnRH) neurone interactions in the median eminence and the control of GnRH secretion. J Neuroendocrinol. 2008;20(6):732–742. 18601696 10.1111/j.1365-2826.2008.01712.x

[261] Ojeda SR, Prevot V, Heger S, Lomniczi A, Dziedzic B, Mungenast A Glia-to-neuron signaling and the neuroendocrine control of female puberty. Ann Med. 2003;35(4):244–255. 12846266 10.1080/07853890310005164

[262] Voigt P, Ma YJ, Gonzalez D, Fahrenbach WH, Wetsel WC, Berg-von der Emde K, Hill DF, Taylor KG, Costa ME, Seidah NG, Ojeda SR Neural and glial-mediated effects of growth factors acting via tyrosine kinase receptors on luteinizing hormone-releasing hormone neurons. Endocrinology. 1996;137(6):2593–2605. 8641214 10.1210/endo.137.6.8641214

[263] Prevot V, Rio C, Cho GJ, Lomniczi A, Heger S, Neville CM, Rosenthal NA, Ojeda SR, Corfas G Normal female sexual development requires neuregulin–erbB receptor signaling in hypothalamic astrocytes. J Neurosci. 2003;23(1):230–239. 12514220 10.1523/JNEUROSCI.23-01-00230.2003 PMC6742140

[264] Garcia-Segura LM, Melcangi RC Steroids and glial cell function. Glia. 2006;54(6):485–498. 16906540 10.1002/glia.20404

[265] Knobil E Remembrance: the discovery of the hypothalamic gonadotropin-releasing hormone pulse generator and of its physiological significance. Endocrinology. 1992;131(3):1005–1006. 1505445 10.1210/endo.131.3.1505445

[266] Plant TM, Barker-Gibb ML Neurobiological mechanisms of puberty in higher primates. Hum Reprod Update. 2004;10(1):67–77. 15005465 10.1093/humupd/dmh001

[267] Ojeda SR, Dubay C, Lomniczi A, Kaidar G, Matagne V, Sandau US, Dissen GA Gene networks and the neuroendocrine regulation of puberty. Mol Cell Endocrinol. 2010;324(1–2):3–11. 20005919 10.1016/j.mce.2009.12.003 PMC2888991

[268] Ojeda SR, Hill J, Hill DF, Costa ME, Tapia V, Cornea A, Ma YJ The Oct-2 POU domain gene in the neuroendocrine brain: a transcriptional regulator of mammalian puberty. Endocrinology. 1999;140(8):3774–3789. 10433239 10.1210/endo.140.8.6941

[269] Mastronardi C, Smiley GG, Raber J, Kusakabe T, Kawaguchi A, Matagne V, Dietzel A, Heger S, Mungenast AE, Cabrera R, Kimura S, Ojeda SR Deletion of the *Ttf1* gene in differentiated neurons disrupts female reproduction without impairing basal ganglia function. J Neurosci. 2006;26(51):13167–13179. 17182767 10.1523/JNEUROSCI.4238-06.2006 PMC6675010

[270] Lee BJ, Cho GJ, Norgren RB Jr, Junier MP, Hill DF, Tapia V, Costa ME, Ojeda SR TTF-1, a homeodomain gene required for diencephalic morphogenesis, is postnatally expressed in the neuroendocrine brain in a developmentally regulated and cell-specific fashion. Mol Cell Neurosci. 2001;17(1):107–126. 11161473 10.1006/mcne.2000.0933

[271] Heger S, Mastronardi C, Dissen GA, Lomniczi A, Cabrera R, Roth CL, Jung H, Galimi F, Sippell W, Ojeda SR Enhanced at puberty 1 (EAP1) is a new transcriptional regulator of the female neuroendocrine reproductive axis. J Clin Invest. 2007;117(8):2145–2154. 17627301 10.1172/JCI31752 PMC1906733

[272] Dissen GA, Lomniczi A, Heger S, Neff TL, Ojeda SR Hypothalamic EAP1 (enhanced at puberty 1) is required for menstrual cyclicity in nonhuman primates. Endocrinology. 2012;153(1):350–361. 22128022 10.1210/en.2011-1541 PMC3249687

[273] Li C, Li P Enhanced at puberty-1 (Eap1) expression critically regulates the onset of puberty independent of hypothalamic Kiss1 expression. Cell Physiol Biochem. 2017;43(4):1402–1412. 29017168 10.1159/000481872

[274] Lomniczi A, Garcia-Rudaz C, Ramakrishnan R, Wilmot B, Khouangsathiene S, Ferguson B, Dissen GA, Ojeda SR A single-nucleotide polymorphism in the *EAP1* gene is associated with amenorrhea/oligomenorrhea in nonhuman primates. Endocrinology. 2012;153(1):339–349. 22128021 10.1210/en.2011-1540 PMC3249686

[275] Xu J, Li P Expression of EAP1 and CUX1 in the hypothalamus of female rats and relationship with KISS1 and GnRH. Endocr J. 2016;63(8):681–690. 27250217 10.1507/endocrj.EJ16-0123

[276] Mueller JK, Koch I, Lomniczi A, Loche A, Rulfs T, Castellano JM, Kiess W, Ojeda S, Heger S Transcription of the human *EAP1* gene is regulated by upstream components of a puberty-controlling tumor suppressor gene network. Mol Cell Endocrinol. 2012;351(2):184–198. 22209758 10.1016/j.mce.2011.12.004 PMC3288847

[277] Lomniczi A, Ojeda SR The emerging role of epigenetics in the regulation of female puberty. Endocr Dev. 2016;29:1–16. 26680569 10.1159/000438840 PMC4955615

[278] Lomniczi A, Loche A, Castellano JM, Ronnekleiv OK, Bosch M, Kaidar G, Knoll JG, Wright H, Pfeifer GP, Ojeda SR Epigenetic control of female puberty. Nat Neurosci. 2013;16(3):281–289. 23354331 10.1038/nn.3319 PMC3581714

[279] Kurian JR, Olesen KM, Auger AP Sex differences in epigenetic regulation of the estrogen receptor-α promoter within the developing preoptic area. Endocrinology. 2010;151(5):2297–2305. 20237133 10.1210/en.2009-0649 PMC2869250

[280] Toro CA, Wright H, Aylwin CF, Ojeda SR, Lomniczi A Trithorax dependent changes in chromatin landscape at enhancer and promoter regions drive female puberty. Nat Commun. 2018;9(1):57. 29302059 10.1038/s41467-017-02512-1 PMC5754362

[281] Zhang X, Kim KM Multifactorial regulation of G protein-coupled receptor endocytosis. Biomol Ther (Seoul). 2017;25(1):26–43. 28035080 10.4062/biomolther.2016.186 PMC5207461

[282] Abreu AP, Macedo DB, Brito VN, Kaiser UB, Latronico AC A new pathway in the control of the initiation of puberty: the *MKRN3* gene. J Mol Endocrinol. 2015;54(3):R131–R139. 25957321 10.1530/JME-14-0315 PMC4573396

[283] Simon D, Ba I, Mekhail N, Ecosse E, Paulsen A, Zenaty D, Houang M, Jesuran Perelroizen M, de Filippo GP, Salerno M, Simonin G, Reynaud R, Carel JC, Léger J, de Roux N Mutations in the maternally imprinted gene *MKRN3* are common in familial central precocious puberty. Eur J Endocrinol. 2016;174(1):1–8. 26431553 10.1530/EJE-15-0488

[284] Hagen CP, Sørensen K, Mieritz MG, Johannsen TH, Almstrup K, Juul A Circulating MKRN3 levels decline prior to pubertal onset and through puberty: a longitudinal study of healthy girls. J Clin Endocrinol Metab. 2015;100(5):1920–1926. 25695892 10.1210/jc.2014-4462

[285] Busch AS, Hagen CP, Almstrup K, Juul A Circulating MKRN3 levels decline during puberty in healthy boys. J Clin Endocrinol Metab. 2016;101(6):2588–2593. 27057785 10.1210/jc.2016-1488

[286] Chevrier L, Guimiot F, de Roux N GnRH receptor mutations in isolated gonadotropic deficiency. Mol Cell Endocrinol. 2011;346(1–2):21–28. 21645587 10.1016/j.mce.2011.04.018

[287] Themmen AP, Huhtaniemi IT Mutations of gonadotropins and gonadotropin receptors: elucidating the physiology and pathophysiology of pituitary-gonadal function. Endocr Rev. 2000;21(5):551–583. 11041448 10.1210/edrv.21.5.0409

[288] Potorac I, Rivero-Müller A, Trehan A, Kiełbus M, Jozwiak K, Pralong F, Hafidi A, Thiry A, Ménagé JJ, Huhtaniemi I, Beckers A, Daly AF A vital region for human glycoprotein hormone trafficking revealed by an LHB mutation. J Endocrinol. 2016;231(3):197–207. 27656125 10.1530/JOE-16-0384

[289] Layman LC, Lee EJ, Peak DB, Namnoum AB, Vu KV, van Lingen BL, Gray MR, McDonough PG, Reindollar RH, Jameson JL Delayed puberty and hypogonadism caused by mutations in the follicle-stimulating hormone β-subunit gene. N Engl J Med. 1997;337(9):607–611. 9271483 10.1056/NEJM199708283370905

[290] Howard SR, Dunkel L The genetic basis of delayed puberty. Neuroendocrinology. Neuroendocrinology. 2018;106(3):283–291. 28926843 10.1159/000481569

[291] Sedlmeyer IL, Hirschhorn JN, Palmert MR Pedigree analysis of constitutional delay of growth and maturation: determination of familial aggregation and inheritance patterns. J Clin Endocrinol Metab. 2002;87(12):5581–5586. 12466356 10.1210/jc.2002-020862

[292] Wehkalampi K, Widén E, Laine T, Palotie A, Dunkel L Patterns of inheritance of constitutional delay of growth and puberty in families of adolescent girls and boys referred to specialist pediatric care. J Clin Endocrinol Metab. 2008;93(3):723–728. 18160460 10.1210/jc.2007-1786

[293] Cousminer DL, Leinonen JT, Sarin AP, Chheda H, Surakka I, Wehkalampi K, Ellonen P, Ripatti S, Dunkel L, Palotie A, Widén E Targeted resequencing of the pericentromere of chromosome 2 linked to constitutional delay of growth and puberty. PLoS One. 2015;10(6):e0128524. 26030606 10.1371/journal.pone.0128524 PMC4452275

[294] Wehkalampi K, Widén E, Laine T, Palotie A, Dunkel L Association of the timing of puberty with a chromosome 2 locus. J Clin Endocrinol Metab. 2008;93(12):4833–4839. 18812480 10.1210/jc.2008-0882 PMC2685475

[295] Xu C, Messina A, Somm E, Miraoui H, Kinnunen T, Acierno J Jr, Niederländer NJ, Bouilly J, Dwyer AA, Sidis Y, Cassatella D, Sykiotis GP, Quinton R, De Geyter C, Dirlewanger M, Schwitzgebel V, Cole TR, Toogood AA, Kirk JM, Plummer L, Albrecht U, Crowley WF Jr, Mohammadi M, Tena-Sempere M, Prevot V, Pitteloud N *KLB*, encoding β-Klotho, is mutated in patients with congenital hypogonadotropic hypogonadism. EMBO Mol Med. 2017;9(10):1379–1397. 28754744 10.15252/emmm.201607376 PMC5623842

[296] Zhu J, Choa RE, Guo MH, Plummer L, Buck C, Palmert MR, Hirschhorn JN, Seminara SB, Chan YM A shared genetic basis for self-limited delayed puberty and idiopathic hypogonadotropic hypogonadism. J Clin Endocrinol Metab. 2015;100(4):E646–E654. 25636053 10.1210/jc.2015-1080 PMC4399304

[297] Howard SR, Oleari R, Poliandri A, Chantzara V, Fantin A, Ruiz-Babot G, Metherell LA, Cabrera CP, Barnes MR, Wehkalampi K, Guasti L, Ruhrberg C, Cariboni A, Dunkel L *HS6ST1* insufficiency causes self-limited delayed puberty in contrast with other GnRH deficiency genes. J Clin Endocrinol Metab. 2018;103(9):3420–3429. 29931354 10.1210/jc.2018-00646 PMC6126894

[298] Parkash J, Messina A, Langlet F, Cimino I, Loyens A, Mazur D, Gallet S, Balland E, Malone SA, Pralong F, Cagnoni G, Schellino R, De Marchis S, Mazzone M, Pasterkamp RJ, Tamagnone L, Prevot V, Giacobini P Semaphorin7A regulates neuroglial plasticity in the adult hypothalamic median eminence. Nat Commun. 2015;6(1):6385. 25721933 10.1038/ncomms7385 PMC4351556

[299] Pielecka-Fortuna J, Chu Z, Moenter SM Kisspeptin acts directly and indirectly to increase gonadotropin-releasing hormone neuron activity and its effects are modulated by estradiol. Endocrinology. 2008;149(4):1979–1986. 18162521 10.1210/en.2007-1365 PMC2276721

[300] Howard SR, Guasti L, Ruiz-Babot G, Mancini A, David A, Storr HL, Metherell LA, Sternberg MJ, Cabrera CP, Warren HR, Barnes MR, Quinton R, de Roux N, Young J, Guiochon-Mantel A, Wehkalampi K, André V, Gothilf Y, Cariboni A, Dunkel L *IGSF10* mutations dysregulate gonadotropin-releasing hormone neuronal migration resulting in delayed puberty. EMBO Mol Med. 2016;8(6):626–642. 27137492 10.15252/emmm.201606250 PMC4888853

[301] Lin L, Conway GS, Hill NR, Dattani MT, Hindmarsh PC, Achermann JC A homozygous R262Q mutation in the gonadotropin-releasing hormone receptor presenting as constitutional delay of growth and puberty with subsequent borderline oligospermia. J Clin Endocrinol Metab. 2006;91(12):5117–5121. 16968799 10.1210/jc.2006-0807 PMC1865483

[302] Vaaralahti K, Wehkalampi K, Tommiska J, Laitinen EM, Dunkel L, Raivio T The role of gene defects underlying isolated hypogonadotropic hypogonadism in patients with constitutional delay of growth and puberty. Fertil Steril. 2011;95(8):2756–2758. 21292259 10.1016/j.fertnstert.2010.12.059

[303] Howard SR, Guasti L, Poliandri A, David A, Cabrera CP, Barnes MR, Wehkalampi K, O'Rahilly S, Aiken CE, Coll AP, Ma M, Rimmington D, Yeo GS, Dunkel L. Contributions of function-altering variants in genes implicated in pubertal timing and body mass for self-limited delayed puberty. J Clin Endocrinol Metab. 2018;103(2):649–659. 29161441 10.1210/jc.2017-02147 PMC5800831

[304] Yeo GS The role of the *FTO* (fat mass and obesity related) locus in regulating body size and composition. Mol Cell Endocrinol. 2014;397(1–2):34–41. 25224490 10.1016/j.mce.2014.09.012

[305] McMurray F, Church CD, Larder R, Nicholson G, Wells S, Teboul L, Tung YC, Rimmington D, Bosch F, Jimenez V, Yeo GS, O’Rahilly S, Ashcroft FM, Coll AP, Cox RD Adult onset global loss of the *Fto* gene alters body composition and metabolism in the mouse. PLoS Genet. 2013;9(1):e1003166. 23300482 10.1371/journal.pgen.1003166 PMC3536712

[306] Merkestein M, Laber S, McMurray F, Andrew D, Sachse G, Sanderson J, Li M, Usher S, Sellayah D, Ashcroft FM, Cox RD FTO influences adipogenesis by regulating mitotic clonal expansion. Nat Commun. 2015;6(1):6792. 25881961 10.1038/ncomms7792 PMC4410642

[307] Smemo S, Tena JJ, Kim KH, Gamazon ER, Sakabe NJ, Gómez-Marín C, Aneas I, Credidio FL, Sobreira DR, Wasserman NF, Lee JH, Puviindran V, Tam D, Shen M, Son JE, Vakili NA, Sung HK, Naranjo S, Acemel RD, Manzanares M, Nagy A, Cox NJ, Hui CC, Gomez-Skarmeta JL, Nóbrega MA Obesity-associated variants within FTO form long-range functional connections with *IRX3*. Nature. 2014;507(7492):371–375. 24646999 10.1038/nature13138 PMC4113484

[308] Fischer J, Koch L, Emmerling C, Vierkotten J, Peters T, Brüning JC, Rüther U Inactivation of the *Fto* gene protects from obesity. Nature. 2009;458(7240):894–898. 19234441 10.1038/nature07848

[309] Speakman JR The “fat mass and obesity related” (*FTO*) gene: mechanisms of impact on obesity and energy balance. Curr Obes Rep. 2015;4(1):73–91. 26627093 10.1007/s13679-015-0143-1

[310] Martínez de Morentin PB, Martinez-Sanchez N, Roa J, Ferno J, Nogueiras R, Tena-Sempere M, Dieguez C, Lopez M Hypothalamic mTOR: the rookie energy sensor. Curr Mol Med. 2014;14(1):3–21. 24236459 10.2174/1566524013666131118103706

[311] Manfredi-Lozano M, Roa J, Ruiz-Pino F, Piet R, Garcia-Galiano D, Pineda R, Zamora A, Leon S, Sanchez-Garrido MA, Romero-Ruiz A, Dieguez C, Vazquez MJ, Herbison AE, Pinilla L, Tena-Sempere M Defining a novel leptin-melanocortin-kisspeptin pathway involved in the metabolic control of puberty. Mol Metab. 2016;5(10):844–857. 27688998 10.1016/j.molmet.2016.08.003 PMC5034608

[312] Fernandez-Fernandez R, Martini AC, Navarro VM, Castellano JM, Dieguez C, Aguilar E, Pinilla L, Tena-Sempere M Novel signals for the integration of energy balance and reproduction. Mol Cell Endocrinol. 2006;254–255:127–132. 10.1016/j.mce.2006.04.026 16759792

[313] Pugliese-Pires PN, Fortin JP, Arthur T, Latronico AC, Mendonca BB, Villares SM, Arnhold IJ, Kopin AS, Jorge AA Novel inactivating mutations in the GH secretagogue receptor gene in patients with constitutional delay of growth and puberty. Eur J Endocrinol. 2011 Aug;165(2):233–241. 21646290 10.1530/EJE-11-0168

[314] Sun Y, Bak B, Schoenmakers N, van Trotsenburg AS, Oostdijk W, Voshol P, Cambridge E, White JK, le Tissier P, Gharavy SN, Martinez-Barbera JP, Stokvis-Brantsma WH, Vulsma T, Kempers MJ, Persani L, Campi I, Bonomi M, Beck-Peccoz P, Zhu H, Davis TM, Hokken-Koelega AC, Del Blanco DG, Rangasami JJ, Ruivenkamp CA, Laros JF, Kriek M, Kant SG, Bosch CA, Biermasz NR, Appelman-Dijkstra NM, Corssmit EP, Hovens GC, Pereira AM, den Dunnen JT, Wade MG, Breuning MH, Hennekam RC, Chatterjee K, Dattani MT, Wit JM, Bernard DJ Loss-of-function mutations in *IGSF1* cause an X-linked syndrome of central hypothyroidism and testicular enlargement. Nat Genet. 2012;44(12):1375–1381. 23143598 10.1038/ng.2453 PMC3511587

[315] Joustra SD, Wehkalampi K, Oostdijk W, Biermasz NR, Howard S, Silander TL, Bernard DJ, Wit JM, Dunkel L, Losekoot M *IGSF1* variants in boys with familial delayed puberty. Eur J Pediatr. 2015;174(5):687–692. 25354429 10.1007/s00431-014-2445-9

[316] Mancini A, Howard SR, Cabrera CP, Barnes MR, David A, Wehkalampi K, Heger S, Lomniczi A, Guasti L, Ojeda SR, Dunkel L EAP1 regulation of GnRH promoter activity is important for human pubertal timing. Hum Mol Genet. 2019;28(5):1357–1368. 30608578 10.1093/hmg/ddy451 PMC6452208

